# Long-read sequencing reveals the RNA isoform repertoire of neuropsychiatric risk genes in human brain

**DOI:** 10.1186/s13059-025-03724-1

**Published:** 2025-09-23

**Authors:** Ricardo De Paoli-Iseppi, Shweta S. Joshi, Josie Gleeson, Yair D. J. Prawer, Yupei You, Ria Agarwal, Anran Li, Anthea Hull, Eloise M. Whitehead, Yoonji Seo, Rhea Kujawa, Raphael Chang, Mriga Dutt, Catriona McLean, Benjamin L. Parker, Michael B. Clark

**Affiliations:** 1 Department of Anatomy and Physiology, The University of Melbourne, Parkville, VIC Australia; 2 Science for Life Laboratory, Department of Gene Technology, KTH Royal Institute of Technology, Stockholm, Sweden; 3 School of Mathematics and Statistics/Melbourne Integrative Genomics, The University of Melbourne, Parkville, VIC Australia; 4 The Walter and Eliza Hall Institute of Medical Research, Parkville, VIC Australia; 5 Department of Anatomical Pathology, Alfred Health, Melbourne, VIC Australia; 6 Victorian Brain Bank, The Florey, Parkville, VIC Australia

**Keywords:** RNA, Splicing, Neuropsychiatric, Brain, Long-read, Nanopore, Isoform

## Abstract

**Background:**

Neuropsychiatric disorders are highly complex conditions and the risk of developing a disorder has been tied to hundreds of genomic variants that alter the expression and/or RNA isoforms made by risk genes. However, how these genes contribute to disease risk and onset through altered expression and RNA splicing is not well understood.

**Results:**

Combining our new bioinformatic pipeline IsoLamp with nanopore long-read amplicon sequencing, we deeply profile the RNA isoform repertoire of 31 high-confidence neuropsychiatric disorder risk genes in Human brain. We show most risk genes are more complex than previously reported, identifying 363 novel isoforms and 28 novel exons, including isoforms which alter protein domains, and genes such as *ATG13* and *GATAD2A* where most expression was from previously undiscovered isoforms. The greatest isoform diversity is detected in the schizophrenia risk gene *ITIH4*. Mass spectrometry of brain protein isolates confirms translation of a novel exon skipping event in ITIH4, suggesting a new regulatory mechanism for this gene in the brain.

**Conclusions:**

Our results emphasize the widespread presence of previously undetected RNA and protein isoforms in the human brain and provide an effective approach to address this knowledge gap. Uncovering the isoform repertoire of candidate neuropsychiatric risk genes will underpin future analyses of the functional impact these isoforms have on neuropsychiatric disorders, enabling the translation of genomic findings into a pathophysiological understanding of disease.

**Supplementary Information:**

The online version contains supplementary material available at 10.1186/s13059-025-03724-1.

## Background

Over 90% of multi-exonic human genes undergo alternative splicing (AS), a process that enables genes to produce multiple mRNA products (RNA isoforms) [1]. Common AS events include exon skipping, intron retention, and alternative 5’ and 3’ exonic splice sites [2]. These mRNA alterations can impact the open reading frame (ORF) and/or alter post-transcription regulation of an RNA, increasing both transcriptomic and proteomic diversity [1, 3, 4]. AS has been established as an important regulator of organ development and physiological functions and is highly regulated under normal conditions [5, 6]. Conversely, aberrant RNA splicing has been linked to the development of cancer, autoimmune, and neurodevelopmental disorders [7–11]. AS plays an especially important role in the brain, which has a distinct splicing program, including the largest number of tissue-specific exons and frequent use of microexons [12]. Numerous studies have reported crucial roles for AS in brain development and dysregulation in disease [13, 14].

Neuropsychiatric or mental health disorders (MHDs) including schizophrenia (SZ), major depressive disorder (MDD), autism spectrum disorder (ASD), and bipolar disorder (BPD) can carry significant morbidity for affected individuals [15]. Comorbidities, delayed diagnoses, and stigma surrounding MHDs also present a significant challenge to individuals and their families [16]. However, treatment options remain limited or are not well tolerated or effective in some individuals, and the underlying aetiology of disease and risk remains poorly understood [17–19]. Recently, genome wide association studies (GWAS) have revealed hundreds of common single nucleotide polymorphisms (SNPs) that are associated with the risk of developing neuropsychiatric disease [20–25]. Confirmatory studies including transcriptome-wide association studies (TWAS), summary data–based Mendelian randomization (SMR) [26], multimarker analysis of genomic annotation (MAGMA) and variants (H-MAGMA [27], nMAGMA [28]), and functional genomics have helped to identify candidate risk genes at these loci and also showed a considerable number of risk loci are shared between disorders [29]. Understanding how risk variants affect risk genes is not straightforward; the vast majority of risk variants are found in non-coding parts of the genome and are expected to be regulatory, impacting gene expression levels or which RNA isoforms are produced. Risk variants may impact splicing factor binding leading to altered isoform splicing ratios [8]. For example, a risk variant block (rs1006737) within intron 3 of the SZ risk gene *CACNA1C* was linked to variable mRNA expression, while *GAD1* long and short isoform expression in the hippocampus was associated with the SZ and ASD risk variant (rs3749034) within the promoter [30, 31]. However, there is a current lack of understanding about how risk gene expression and splicing are altered by the risk variants, and therefore profiling both their expression and RNA isoforms is essential to link genetic changes to disease pathophysiology.

Current sequencing technologies including Illumina short-reads perform well at detecting novel AS. However, the lack of long-range exon connectivity information inherent in short-reads means these approaches are limited in their ability to identify and quantify full-length isoforms, and this issue is exacerbated in longer, more complex genes [32, 33]. In contrast, long-read technologies including Oxford Nanopore Technologies (ONT) and Pacific Biosciences (PacBio) can sequence entire isoforms in a single read enabling more accurate isoform profiling [7, 34, 35]. Such technologies now make it feasible to comprehensively examine gene isoform profiles. Initial investigations of *SNX19* and *CACNA1C* demonstrated the incomplete knowledge of isoform profiles in humans and the likely importance of novel gene isoforms in disease risk [36, 37].

In this study, we addressed the lack of knowledge surrounding MHD risk gene isoform expression using nanopore amplicon sequencing. We developed a new bioinformatic tool, IsoLamp, to identify known and novel RNA isoforms from long-read data. Analysis of the RNA splicing profiles of 31 candidate MHD risk genes identified 363 novel RNA isoforms and 28 novel exons. We identified several genes where most expression is from novel isoforms, including *ATG13* and *GATAD2A*, where the most highly expressed isoforms were novel. Our results show the transcript structure for most risk genes is more complex than current annotations, containing additional exon skipping events, retained introns, novel splice sites, and novel exons, including novel isoforms that alter the protein and potentially its function. This work lays the foundation for a better understanding of how risk gene isoforms may play a role in disease pathophysiology.

## Results

### Experimental overview

**Fig. 1:**
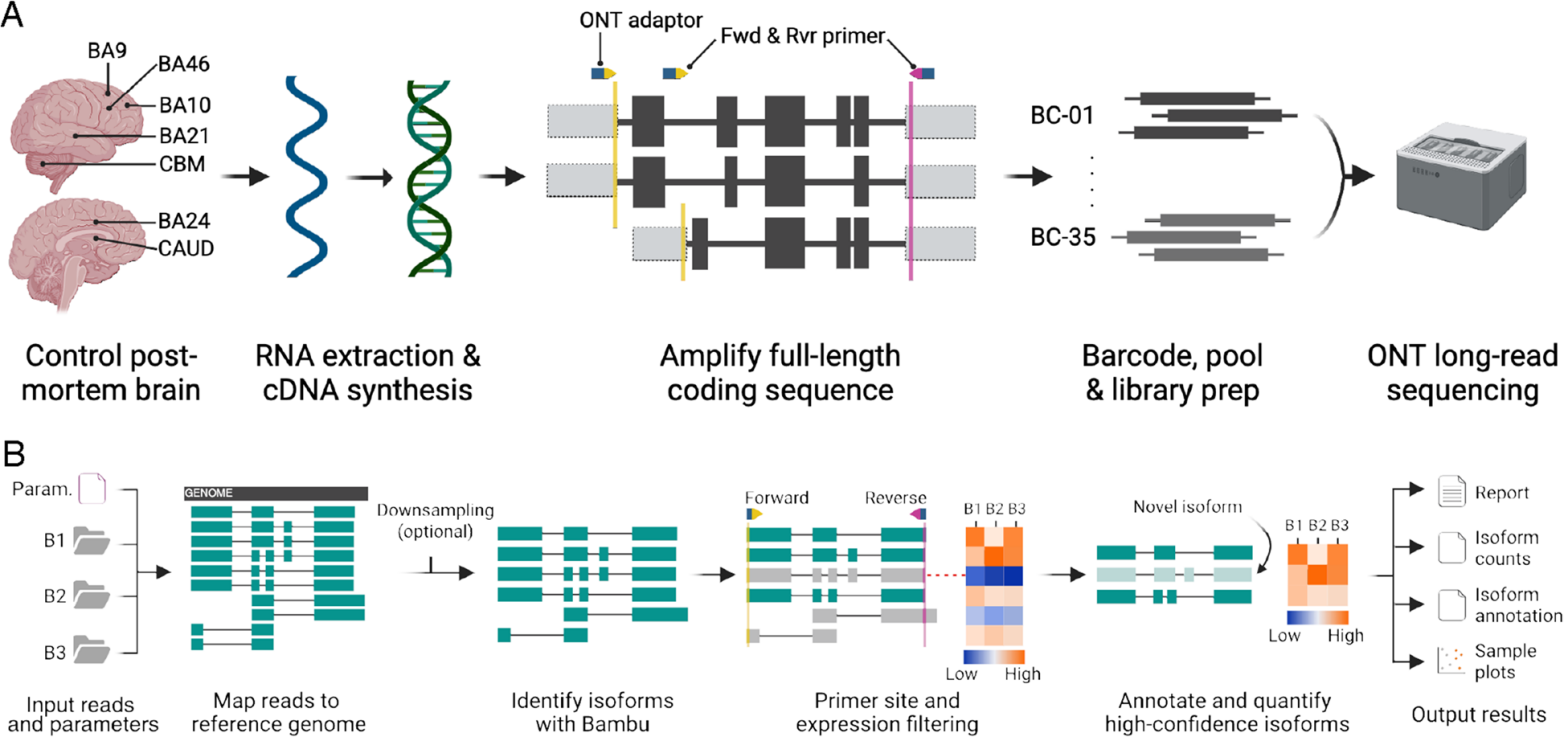
**A **RNA isoform sequencing of human post-mortem brain. RNA was isolated from frontal cortical regions, caudate (CAUD), and cerebellum (CBM) and converted to cDNA. The coding sequence (black boxes) was amplified using specific forward (Fwd, yellow arrows) and reverse (Rvr, pink arrow) primers generally designed in the 5’ and 3’ UTR regions (grey boxes) to capture as many isoforms as possible. An Oxford Nanopore Technologies (ONT) adaptor sequence (blue box) was incorporated into each primer for sample multiplexing. Samples were then barcoded and pooled to create a single library for long-read sequencing on a GridION. Key: Brodmann Area (BA), barcode (BC). **B** Isoform discovery with long-read amplicon sequencing (IsoLamp) workflow. A gene specific parameters file (containing chromosome and primer coordinates) was used to align long-reads from each sample (B1-3) against the reference genome (black box) using Minimap2. Known and novel RNA isoforms were identified using Bambu. Identified isoforms are then filtered (grey isoforms) to remove: (1) those not overlapping forward (yellow line) and reverse (pink line) primer positions, ensuring full-length isoform discovery; (2) Lowly expressed isoforms (blue on heatmap, indicated by dashed red line), which do not meet an expression threshold in a specified proportion of samples (both settings user-defined). Filtered known and novel isoforms are then annotated, quantified, and IsoLamp results files generated

To identify the RNA isoforms expressed from candidate MHD risk genes, we aimed to perform long-read amplicon sequencing, which provides a highly sensitive means for comprehensive isoform discovery and relative quantification (Fig. 1A) [36]. We selected seven regions of post-mortem human brain from five control individuals, encompassing both transcriptionally divergent regions as well as those highly implicated in MHDs (Additional file 1: Table S1). Amplicons were designed to cover the full coding region of target genes and, where possible, run from the first to the last exon. Multiple sets of primers were used for genes with alternative transcriptional initiation and termination exons and/or alternative coding sequence initiation and termination sites to profile as many potential alternative isoforms as possible.

### IsoLamp: a tool for RNA isoform discovery from long-read amplicon sequencing

While there are several long-read isoform discovery and quantification tools, these are not generally optimized for amplicon sequencing of single genes at high depth. Therefore, we created ISOform discovery with Long-read AMPlicon sequencing** (**IsoLamp), a custom pipeline designed for isoform profiling from amplicon sequencing (Fig. 1B) [38]. In contrast to previous tools [36], IsoLamp provides flexible filtering options and a simpler, unified output of isoforms; it can be applied to any gene and is easy to install and run.

**Fig. 2:**
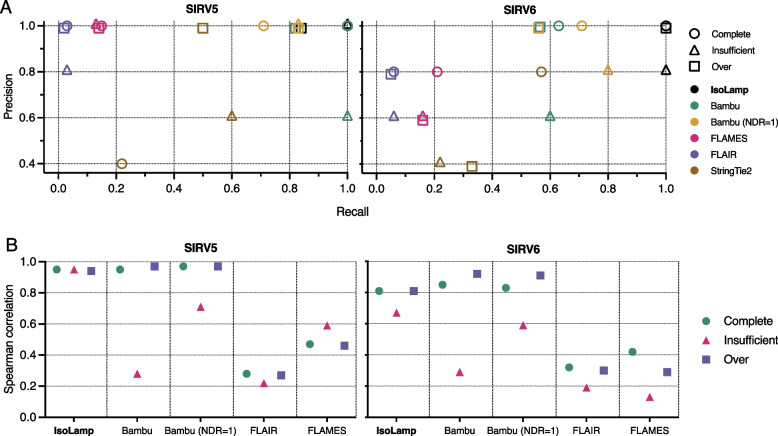
Benchmarking IsoLamp using spike-in SIRVs. **A** Precision recall of each tested pipeline with the Complete (*N* = 69), Insufficient (*N* = 43) or Over-annotated (*N* = 100) SIRV references. IsoLamp (black) returned high-quality isoforms from amplicon data of both SIRV5 and 6. **B** SIRV5 and 6 Spearman correlations between known and observed expression values for each bioinformatic tool using the SIRV complete (green), insufficient (pink) and over (blue) reference annotations

We optimized the performance of IsoLamp using synthetic Spike-in RNA variants (SIRVs) that provide a known ground truth for isoform exonic structures and abundances and then benchmarked it against other tools. We performed long-read amplicon sequencing on SIRV5 and SIRV6, targeting five isoforms per synthetic gene, as these SIRVs allowed targeting of the largest number of isoforms with a single primer pair and so best recapitulated human genes. The SIRV dataset comprised nine replicates from each of the three SIRV mixes (E0, E1, E2) for each gene (Additional file 2: Fig. S1). 99% of reads mapped to the SIRV genome with minimap2 [39], confirming on-target amplification. We benchmarked the performance of IsoLamp with Bambu [40], FLAIR [41], FLAMES [42], and Stringtie2 (-L) [43]. We assessed the precision, recall, and quantitative accuracy of the five tools using three different SIRV reference annotations provided by Lexogen (Fig. 2, Additional file 1: Table S2, Methods): (1) Complete—contains all SIRV isoforms (SIRV_C, N = 69); (2) Insufficient—missing 26 SIRV isoforms that are present in the mixes (SIRV_I, *N* = 43); and (3) Over—contains an additional 31 isoforms that are not present in the SIRV mixes (SIRV_O, *N* = 100).

Our benchmarking results demonstrated IsoLamp had the highest precision and recall values, consistently outperforming other isoform discovery tools by correctly identifying true isoforms and minimizing false positives (Fig. 2A, Additional file 1: Table S2). This included maintaining high performance with the more challenging, but also more realistic, Insufficient and Over-annotation references. IsoLamp expression quantification was also consistently accurate and maintained performance irrespective of the annotation provided (Fig. 2B).

Bambu, which is also utilized within the IsoLamp pipeline, is the next best performing tool, although it identified more false positives and had poorer recall and quantification results using the Insufficient annotation (Fig. 2). IsoLamp utilizes Bambu parameters optimized for amplicon sequencing, including a novel discovery rate (NDR) of 1. Adjusting the Bambu “NDR” to 1 improved its recall but did not improve precision (Fig. 2, Additional file 1: Table S2). These results demonstrate how IsoLamp outperforms tools designed for whole-transcriptome analysis, including when Bambu is provided with optimized isoform discovery parameters for amplicon sequencing.

FLAIR had the highest number of isoforms of all tools tested identifying 261, 181, and 278 novel transcripts in the Complete, Insufficient, and Over-annotated reference-based analyses, respectively. This high level of false-positive novel transcripts led to inaccuracies in transcript abundance assignments, resulting in low correlations compared to other tools (Fig. 2). FLAMES exhibited 100% recall for SIRV5 across all annotations; however, its performance with SIRV6 was suboptimal, indicating a higher degree of variability in the FLAMES isoform discovery pipeline. FLAMES also performed poorly for isoform quantification. Lastly, while Stringtie2 did not introduce large numbers of false positives, it had the highest number of false negatives, including when provided with the Complete annotation (Fig. 2, Additional file 1: Table S2).

IsoLamp employs an expression-based filter to remove isoforms that do not meet a user-defined expression threshold in a specified proportion of samples (Fig. 1B, see Methods). The default values of this filter were optimized based on the removal of known false positives from the SIRV benchmarking data (SIRV_O) described above and enable IsoLamp to remove possible false positive isoforms in amplicon data. Application of this filter to Bambu, FLAIR, and FLAMES substantially reduced false positive novel isoforms and enhanced overall precision (Additional file 2: Fig. S2, Additional file 1: Table S2); however, IsoLamp was still the top performing tool. Beyond synthetic benchmarking data, reference annotations are typically a combination of insufficient and over annotations. In such scenarios, IsoLamp demonstrated the best overall performance (Fig. 2, Additional file 2: Fig. S2), signifying its superiority for amplicon-sequencing based isoform discovery and quantification from real biological data.

### Post-mortem human brain RNA quantity and quality

Total RNA for long-range amplicon sequencing was extracted from 7 brain regions from 5 healthy individuals (Ind01–05) and subject to sample QC (Additional file 2: Fig. S3A–D). RINe (mean = 7.4, range = 6–8.1) did not differ by brain region; however, Ind04 had significantly lower RINe scores (Additional file 2: Fig. S3B). No trend between the PMI (mean = 44.25 h) and RINe was observed (Additional file 2: Fig S3C). As expected, RINe appeared to worsen with decreasing brain tissue pH levels, though all samples had acceptable RINe and pH values (Additional file 2: Fig. S3D) [44]. Principal component analysis (PCA) showed separation of the cerebellum from cortical regions in PC1 and Ind04 (likely driven by lower sample pH and RINe) in PC2 (Additional file 2: Fig. S5AB). A relatively small proportion of variance (4.7%) was attributed to control donor age in PC6 (Additional file 2: Fig. S4C).

### Long read sequencing identifies 363 novel RNA isoforms

**Fig. 3:**
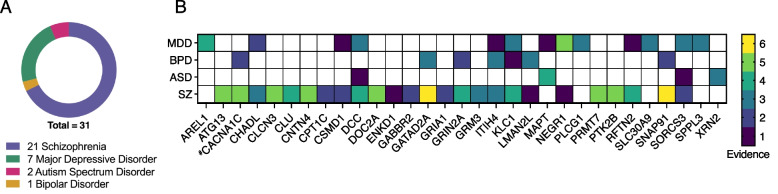
Selection of high-confidence candidate MHD risk genes for amplicon sequencing. **A** Risk genes included in this study classified by the disorder for which they have the highest evidence of association. **B** Sequenced genes and their evidence levels for each MHD. The evidence count was calculated as the sum of independent analysis types, for example: GWAS; MAGMA; TWAS; SMR; DNA methylation; fine mapping; protein–protein interaction; and targeted validation studies, that supported gene involvement in risk for a particular disorder. ^#^Re-sequencing of a gene from a previous study [36]

A total of 31 candidate risk genes were selected for amplicon sequencing based on the accumulated evidence for their involvement in neuropsychiatric disorder risk. A custom database of candidate risk genes and their evidence levels was created, and genes ranked (Additional file 2: Fig. S5, Methods). In a reflection of current GWAS cohort sizes, 21 of the selected genes had the highest evidence for involvement in risk for SZ, 7 for MDD, 2 for ASD, and 1 for BPD (Fig. 3A). Evidence from GWAS, TWAS and other studies show that some genes appear to be risk factors for multiple disorders including *KLC1* for SZ, MDD, and ASD (Fig. 3B).

**Fig. 4:**
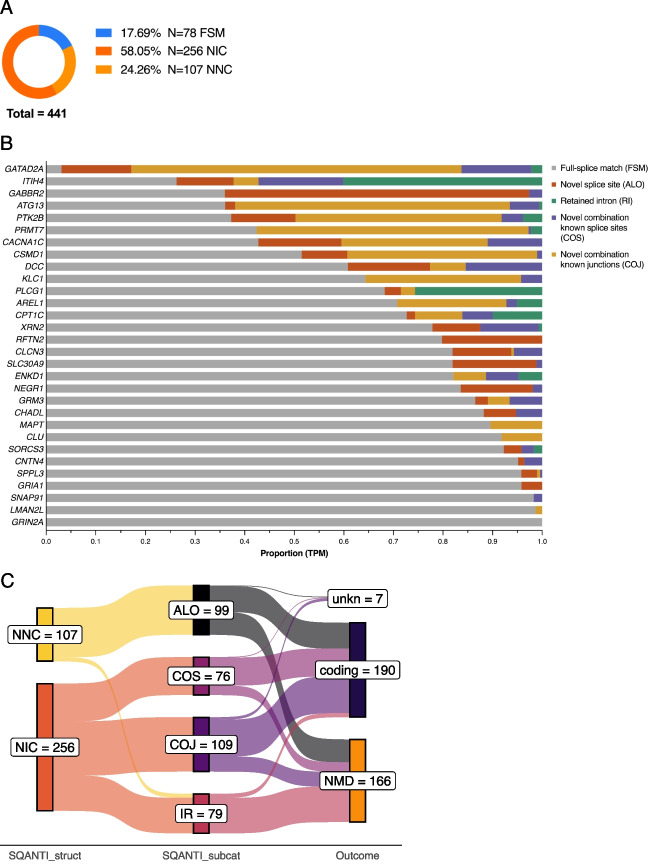
**A** The total number of known and novel isoforms identified across 31 risk genes. SQANTI structural categories are known/full splice match (FSM), novel in catalogue (NIC) and novel not in catalogue (NNC). **B** Proportion of reads (transcript per million, TPM) for each gene as classified by the SQANTI sub-category. **C** Count of predicted outcomes for novel isoform subcategories. Expasy [48] was used to examine the open reading frame (ORF) of novel isoforms (SQANTI structural category: novel not in catalogue (NNC) or novel in catalogue (NIC)) using the canonical start and stop as a reference. Predictions were categorized as coding if the ORF was retained, nonsense mediated decay (NMD) if a premature termination codon was present and not within 50 nt of the final exon junction, or unknown (unkn) if there was not enough information. Novel isoform SQANTI subcategories (subcat) are at least one novel splice site (ALO), intron retention (IR) and combination of known junctions (COJ) or splice sites (COS)

The full RNA isoform profile for each gene was sequenced using nanopore long-read amplicon sequencing. Mapping accuracy ranged from 93.7% (*CLCN3*) to 97.5% (*SORCS3*) (Additional file 2: Fig. S6AB). Each novel isoform and its predicted impact on known protein domains, the open reading frame (ORF) and associated instability index was recorded (Additional file 3) and visualized using IsoVis (Additional file 4) [45]. With no expression filter, IsoLamp identified 872 known and novel isoforms across all 31 neuropsychiatric disorder risk genes. To filter this List for high-confidence isoforms, we applied the IsoLamp expression filter, which resulted in 441 known and novel isoforms across all genes (Fig. 4A). Of these, SQANTI [46, 47] classified 78 as known (full splice match (FSM)), 256 as novel but using known splice sites or junctions (novel in catalogue (NIC)), and 107 as containing at least one novel splice site (novel not in catalogue (NNC)) (Fig. 4A).

The IsoLamp expression filter required identified isoforms to be present in 25% of samples. We confirmed IsoLamp was only identifying replicable isoforms by running it with the *samples_minimum* parameter set to the following: 1 (3% samples), 2 (6%), and 35 (100%) for selected genes. For *DCC*, we identified 3 isoforms that were only expressed in 1 sample, all of which were removed by our default filtering (Additional file 2: Fig. S7A). Similar outcomes were shown for *ATG13*, *GRIA1*, and *CLCN3* demonstrating that isoforms arising from a single sample, or with low sample support, were being effectively removed (Additional file 2: Fig. S7B–D).

We next asked what proportion of reads for each gene were assigned to novel isoforms (Fig. 4B). This ranged widely from approximately 96.9% for *GATAD2A* to 0% for *GRIN2A*, which was the only gene for which no novel RNA isoforms were detected. Approximately one quarter (7/31) of genes investigated had most of their gene expression assigned to novel isoforms, demonstrating how isoforms and their expression profiles for many genes are still poorly understood. As our amplicon sequencing does not encompass all variations in transcriptional initiation and termination sites, these results can be seen as a lower bound for the number of novel isoforms (Additional file 2: Fig. S8). Linear regression of gene isoform counts and novel isoform proportions did not reveal a significant relationship with amplicon length or canonical exon count, indicating that detection of novel isoforms is largely gene dependent (Additional file 2: Fig. S9A–D). To determine what was different about the splicing pattern of each novel isoform, we further sub-classified them using SQANTI, based on the use of a combination of known exon junctions (COJ) or splice sites (COS), retained intron (RI) or containing at least one novel splice site (ALO) (donor, acceptor or pair) [46, 47]. Overall, the most reads were assigned to “novel combination of known junctions,” where all individual exon combinations were known but the entire chain of exons was novel. The type and proportion of novel isoforms from each category was highly gene specific, demonstrating a wide variety of novel RNA types missing from current gene annotations.

The impact of each novel isoform on the encoded ORF was examined using Expasy [48] and recorded as retaining the canonical or other known reading frame (coding), Likely-NMD or unknown. Novel isoforms were classified as coding for 54.2%, 67.3%, and 75.4% for ALO, COJ, and COS subcategories, respectively. We identified 49 novel isoforms that contained retained introns, 39 (83%) of which were predicted to lead to NMD (Fig. 4C). Our results were also useful for several genes in identifying the probable isoforms represented by incomplete GENCODE transcripts. For clusterin (*CLU*), the novel Tx1 (COJ) extended ENST00000520796 to the canonical stop codon and suggested this isoform is moderately abundant (8.2% of TPM) across all brain tissues. The ASD risk gene microtubule associated protein tau (*MAPT*) novel Tx5 extended ENST00000703977 and further demonstrated that inclusion of canonical exon 7 (chr17:45,989,878–45,990,075) does not always exhibit coordinated splicing with canonical exon 5. This isoform had moderate expression comprising 3.2% of *MAPT* TPM.

Isoforms that contained “at least one novel splice site” (ALO) generally contained a novel deletion within a known exon or had novel donors and/or acceptors. All novel junctions in ALO isoforms were canonical GT-AG, GC-AG or AT-AC junction pairs, though often only the splice donor (GT) or acceptor (AG) was novel, for example a novel splice acceptor (+ 98 nt) for *CPT1C* exon 17 (Additional file 2: Fig. S10AB). We found that ~ 48% of ALO isoforms contained either a single novel splice donor or acceptor. Novel GC-AG pairing was detected in two SZ risk genes, within the 5’UTR of *GABBR2* and the donor site of a validated novel exon in *RFTN2*. These results show a clear advantage of using long-read sequencing to contextualize novel splice sites, which aids in predicting the outcome on the isoform and ORF.

To confirm that novel isoforms were not specific to putative risk genes, we also sequenced two genes, *PACS2* and *PREX1*, with little evidence for neuropsychiatric disorder risk (e.g., not found in our candidate risk gene list). In total, we identified 16 RNA isoforms, with 10 (~ 30% of overall TPM) and 2 (~ 44% overall TPM) novel isoforms for *PACS2* and *PREX1*, respectively (Additional file 4: Fig. 2AF–AG). These results demonstrate that identification of unannotated RNA isoforms is not limited to risk genes.

### Detection of highly expressed novel isoforms

A key question regarding novel isoforms is whether they are expressed at a high enough level to impact the biological function of a gene. This is a complex question, because a novel isoform could be low at the tissue level but highly abundant in a specific cell type, or multiple expressed novel isoforms can be significant cumulatively, especially if they all encode the same change to a protein. Therefore, we focused on genes with significant individual or cumulative expression of novel isoforms (analysis on all gene isoforms is available in Additional files 3 and 4).

**Fig. 5:**
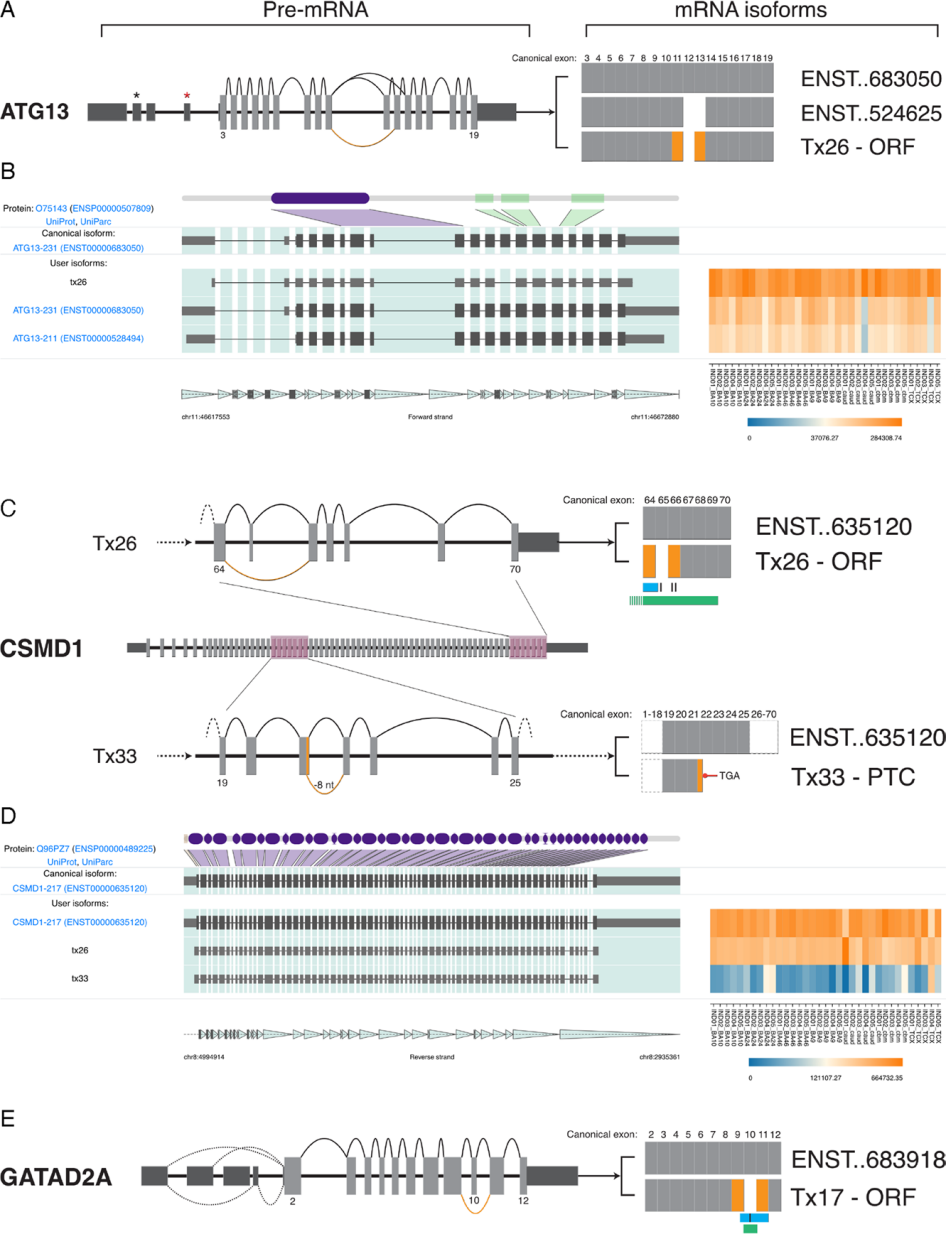
Highly abundant novel isoforms and the predicted mRNA outcome. **A**, **C**, **E** mRNA splice graphs. Dark and Light grey boxes indicate 5’ and 3’ UTR and coding exons respectively. Numbers indicate the coding exon of interest. Orange arcs (pre-mRNA) and boxes (mRNA) indicate novel splicing events. mRNA isoforms depict known isoforms (ENST) against novel (Tx) isoforms; the symbol “..” indicates abbreviated zeroes. **B**, **D** IsoVis visualization of isoform structures (center stack) and expression levels (heatmap). Canonical isoform shown at top of stack including exonic mapping of protein domains (purple) and disordered regions (green) **A** Splice graph of *ATG13* highlights the open reading frame (ORF) preserving skipping event of canonical exon 12. **B** High expression of *ATG13* novel transcript 26 (Tx26). **C** Splice graphs highlighting novel changes in *CSMD1* novel transcript 26 (Tx26) and 33 (Tx33) within highlighted pink regions. The ORF retaining skipping event of canonical exon 65 may disrupt a known glycosylation site (black bar), a sushi domain extending from exon 64 (blue) and part of an extracellular domain (green). Tx33 contains a novel splice donor (−8 nt) within exon 21 leading to a premature termination codon (PTC) in exon 22. Dashed Lines indicate continuation of the transcript to 5’ or 3’ coding exons. **D** Relatively high expression of *CSMD1* novel transcripts 26 and 33. **E** *GATAD2A* novel transcript Tx17 contained a novel, ORF retaining, skipping event of canonical exon 10 which contains a phosphorylation site (black bar), part of a polar biased region (blue), and overlaps a CpG island (< 300 bp, green). Dashed Lines indicate alternative splicing of 5’UTR exons

We identified 22 novel isoforms for the schizophrenia risk gene autophagy-related protein 13 (*ATG13*). Novel isoforms represented 64% of gene expression, compared to 36% for full-splice matches. The most abundant class of novel isoforms (15/22) were COJ, which Made up 55.4% of gene expression (Fig. 4B). *ATG13* had two alternative splicing hotspots: firstly within the 5’UTR and secondly around a predicted disordered region involving exons 12 and 13 in the canonical isoform. Across all brain regions, the most highly expressed isoform was the novel COJ transcript 26 (Tx26), which represented 23% of TPM, surpassing the canonical transcript ENST00000683050 (12.8%). Tx26 differs from the canonical transcript by skipping exon 12 (Fig. 5A, B). It contains the same CDS as ENST00000359513 but includes an additional exon in the 5’UTR (exon 3). Novel COJ transcripts 6 and 8 also had high TPMs and together accounted for 16.6% of expression. These isoforms were novel due to a combination of 5’UTR exons not previously seen within full-length GENCODE annotations.

The schizophrenia risk gene CUB and sushi multiple domains 1 (*CSMD1*) was the longest CDS we amplified at 10,838 nt, encompassing 70 coding exons. In total, 8/9 detected isoforms were classified as novel (Additional file 4: Fig. I). Following the canonical isoform (ENST00000635120, 51.5% of TPM), novel transcripts 26 (COJ) and 33 (ALO) accounted for 38.3% and 7.1% of isoform TPMs, respectively (Fig. 5C, D). Novel Tx26 skipped known exon 65, which encodes a sushi 28 extracellular domain and glycosylation site. The ORF of Tx26 retained the reading frame encoding a 3549 amino acid (aa) protein. Novel Tx33 contained a novel splice donor (GT, −8 nt) in canonical exon 21 and was predicted to encode a PTC in canonical exon 22. The full Tx33 mRNA also skipped canonical exon 65. *CSMD1* also provides a useful example of the benefit of long reads for profiling isoforms. GTEx isoform expression data (https://www.gtexportal.org/home/gene/CSMD1) for *CSMD1* in brain is almost exclusively assigned to isoforms with downstream transcriptional initiation sites (including the two-exon fragment ENST00000521646), despite splice junction level expression largely supporting expression from the canonical start site. This emphasizes the difficulty of assembling and quantification expression of full-length isoforms from long, complex genes, which can be achieved using long isoform spanning reads.

The chromatin remodelling subunit and shared SZ and BPD risk gene GATA zinc finger domain containing 2 A (*GATAD2A*) had one of the highest proportions of reads (96.9%) assigned to novel isoforms (Additional file 4: Fig. N). Many novel isoforms were predicted to be coding COJs (10/24), and these also accounted for the majority of novel expression (66.6%). Novel Tx17 had the highest expression level (22.7%) of any *GATAD2A* isoform and skipped canonical exon 10 which overlaps a CpG island (212 nt, 21.7% CpG) and contains a disordered, polar residue biased region and a phosphorylation site (Fig. 5E). Two additional novel isoforms (Txs 8 and 12), together accounting for 19.9% of expression, incorporated a known 89 nt 5’UTR exon (ENST00000494516) into full-length isoforms for the first time, clarifying the isoforms expressed from this gene. It is important to note that there are two known start sites for this gene supported by high levels of CAGE reads and Human mRNAs. The forward primer used in this study was located within the 5’UTR of ENST00000360315 and as such expression levels of alternatively spliced isoforms from ENST00000683918 are not included in this analysis (Additional file 1: Table S3).

Several additional genes had relatively high levels of at least one novel isoform including the SZ risk genes protein tyrosine kinase 2 beta (*PTK2B*) and protein arginine methyltransferase 7 (*PRMT7*). *PTK2B* novel transcripts 25 and 11 together accounted for ~ 42% of total expression, and both had variable splicing, including a novel donor (GT, + 141nt) of the 5’UTR exon 5 (ENST00000519650) (Additional file 4: Fig. Y). *PRMT7* novel transcript 18 (12.3% of TPM) skips canonical exon 4 which may lead to NMD or alternatively, use a supported (ENST00000686053) translation start site in the following exon (Additional file 4: Fig. X).

The full RNA isoform profile for the SZ risk gene calcium voltage-gated channel subunit alpha1 C (*CACNA1C*) has previously been reported and is repeated in this study [36]. In total, we identified 5 annotated and 22 novel isoforms. The most highly expressed novel isoform identified in our previous study, “novel 2199,” now known as ENST00000682835.1 was also identified in our samples and exhibited similar cerebellar enriched expression. Importantly, 10 novel isoforms replicated one of two alternative splicing events in a hotspot identified previously in canonical exon 7 [36]. This hotspot contains the canonical splice site and two alternative 3’SS acceptors over only 12 nucleotides (chr12: 2,493,190–2,493,201) (Additional file 2: Fig. S11).

### Novel isoforms alter predicted protein structures

Novel isoforms have the potential to affect either post-transcriptional regulation and/or protein sequence, structure, and function. We next investigated a selection of isoforms that would be predicted to lead to protein changes to understand their possible impact.

**Fig. 6:**
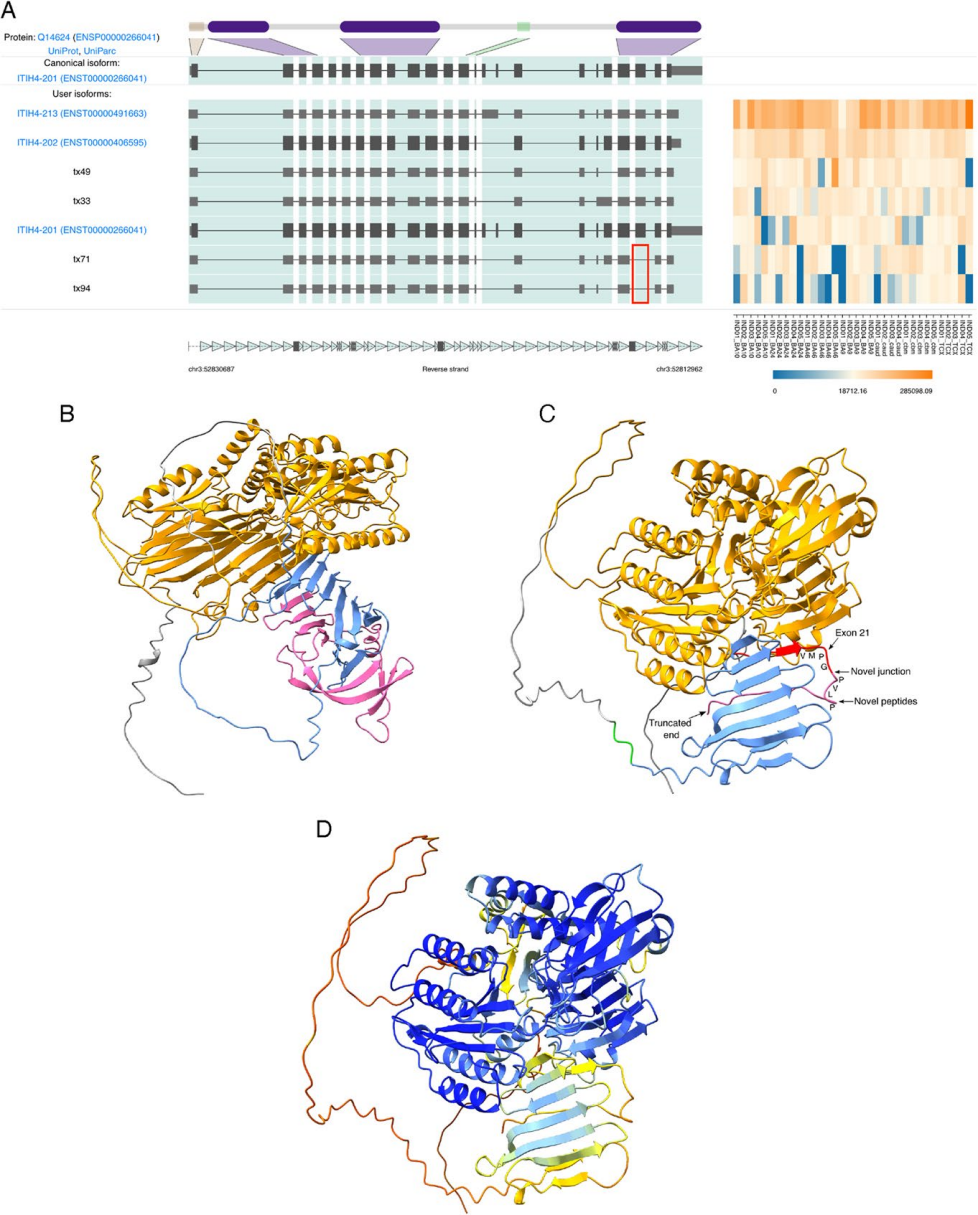
*ITIH4 *canonical and novel isoform protein structure predictions. **A** IsoVis stack of the top seven *ITIH4* isoforms sorted by expression. Several novel isoforms contained the novel exon 22 skipping event (red box) including Txs71 and 94. **B** Canonical isoform (ENST00000266041, UniProt:Q14624) structure prediction indicating 70 kDa (orange) and 35 kDa (blue and pink) chains. **C** Novel isoform (Tx71) structure prediction indicating a 70 kDa chain (orange), truncated 35 kDa chain (blue), O-glycosylation sites (green), novel splice junction peptide detected using mass spectrometry (red), and novel peptides (pink). Black arrow indicates termination < 50 nt from the final exon junction complex. **D** AlphaFold per-residue confidence scores (pLDDT) (0–100) for *ITIH4* novel transcript 71: very high (> 90, blue), confident (90–70, light-blue), low (70 > 50, yellow), and very low (< 50, orange)

Several novel isoforms (including 5 of the top 20 by expression, Additional file 4: Fig. R) predicted a novel exon 22 skipping event in the SZ and MDD risk gene *ITIH4*. Targeted mass spectrophotometry (MS) confirmed a novel junction between exons 21 and 23 (ETLFSVMPG//PVLPGGALGISSSIR) created due to skipping of exon 22 (Fig. 6A, Additional file 2: Fig. S12). This event was predicted to encode a PTC < 50 nt from the final exon junction, indicating it may not be directed to NMD. Protein structure prediction of the canonical (ENST00000266041.9) and a representative novel isoform (Tx71) indicated a loss of 106 aa (~ 44%) of the 35 kDa heavy chain domain but retention of three O-glycosylation sites (Thr:719, 720, 722) (Fig. 6B–D). Novel transcript 71 accounted for ~ 3.7% of *ITIH4* TPM, and this skipping event was found in an additional 24/68 (~ 35%) novel isoforms which together accounted for 23.4% of TPM. Tx71 also skipped canonical exons 15 and 16, which contain a protease susceptibility region (residues 633–713) and a MASP-1 cleavage site (645 – 646: RR) [49]. Cleavage at this site and subsequent formation of an ITIH4-MASP complex can inhibit complement activation via the lectin pathway [49]. The absence of much of the 35 kDa heavy chain domain is likely to impact on ITIH4 protein function, and further studies will be required to examine if it plays a role in neuronal phenotypes.

Using untargeted MS, we also confirmed a novel skipping event of cassette exon 5 (244–266) in gamma-aminobutyric acid type B receptor subunit 2 (GABBR2), which was identified in novel Tx29 (Additional file 4: Fig. M). The Tx29 protein is missing multiple features including an alpha helix, beta strand and disulfide bond forming cysteine (position 266) (Additional file 2: Fig. S13A–C). The correct formation of GABBR2 subunits is critical for assembly of the human gamma-aminobutyric acid type B receptor (GABABR), which plays an important role in inhibitory neurotransmission in the brain [50]. Disruption of the GABBR2 structure in Tx29 may negatively impact this receptor; however, further functional testing is required.

Ten novel isoforms were identified for the SZ risk gene glutamate metabotropic receptor 3 (*GRM3*). Three novel isoforms (Txs 6, 7, 9) skip exon 2 which contains the canonical translation start site. These isoforms instead could use an alternative, frame-retaining, translation initiation site in exon 1, extending the truncated reference isoform ENST00000454217.1, which is also supported by human amygdala mRNA (AK294178) (Additional file 4: Fig. Q). Translation of these isoforms could cause significant disruption to the resultant protein with removal of the signal peptide, transmembrane domain, and disulfide bonds. Cumulatively, these novel isoforms accounted for a relatively low 8.8% of expression when compared to the canonical isoform (86.5%).

**Fig. 7:**
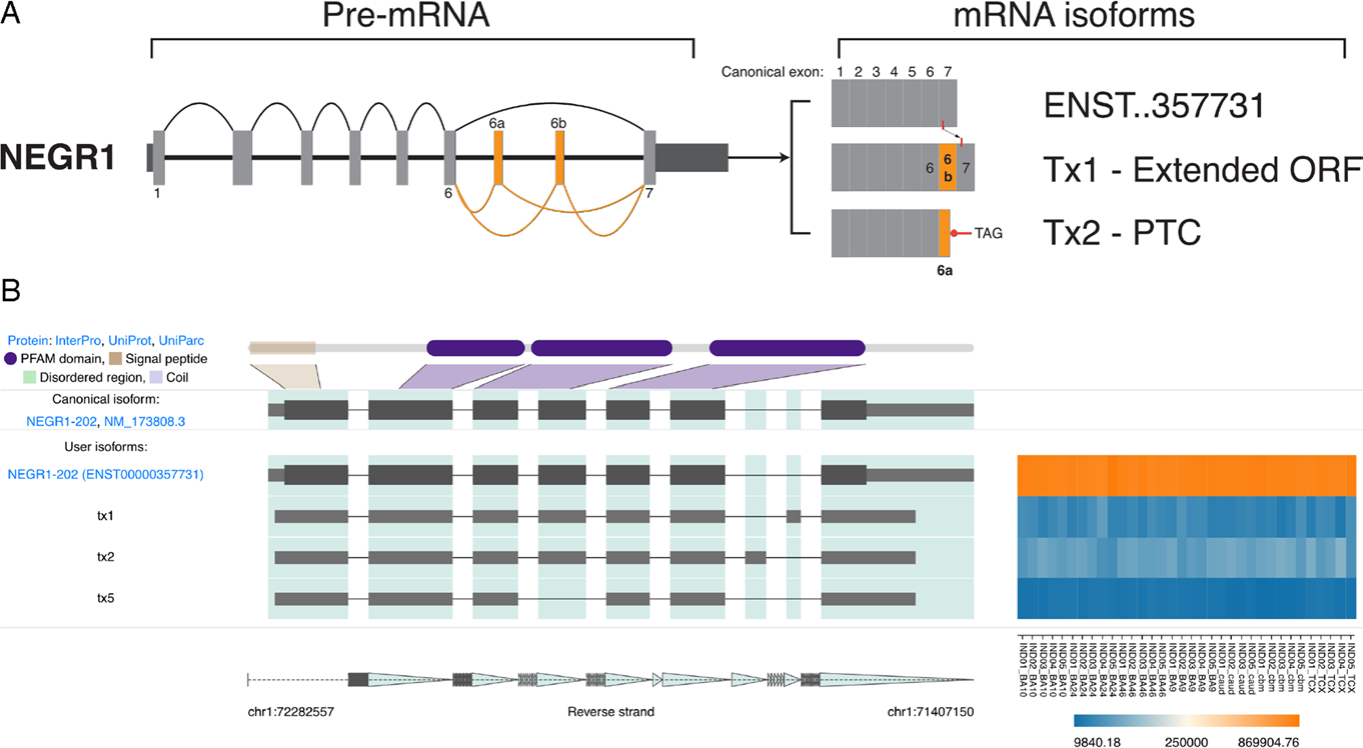
*NEGR1* splice isoforms in human brain. **A*** NEGR1* mRNA splice graph highlighting validated novel exons 6a and 6b. Dark and Light grey boxes indicate 5’ and 3’ UTR and coding exons respectively. Numbers indicate the coding exon of interest. Orange arcs (pre-mRNA) and boxes (mRNA) indicate novel splicing events/exons. mRNA isoforms depict known isoforms (ENST) against novel (Tx) isoforms; the symbol “..” indicates abbreviated zeroes. In the open reading frame (ORF) retaining Tx1, a GPI anchor (red line) is shown to shift 3’ in the final exon when compared to ENST00000357731. Tx2 encodes a premature termination codon (PTC) within the novel exon. **B** IsoVis visualization of *NEGR1* isoform structures (center stack) and expression levels grouped by brain region (heatmap). Canonical isoform shown at top of stack including exonic Mapping of a 5’ signal peptide (brown) and three immunoglobulin (Ig)-like domains (purple). Canonical 3’ UTR has been trimmed

Both novel isoforms and exons were identified for the shared MDD and ASD risk gene neuronal growth regulator 1 (*NEGR1*). Most reads (83.5%) were assigned to the canonical *NEGR1* isoform (ENST00000357731, 354 aa). Three novel isoforms were identified, two of which (Txs 1 and 2) contained novel exons (Fig. 7A, B). These transcripts accounted for 9.4% (Tx2) and 5.1% (Tx1) of TPM. Both novel exons were located between cassette exons 6–7 and were validated using Sanger sequencing. The novel exon within Tx1 was 42 nt (14 aa) in length, had high 100 vertebrate conservation (UCSC) and was predicted to be frame retaining (Additional file 2: Fig. S14A). Protein structure prediction of the 368 aa Tx1 using AlphaFold [51] showed a 14 aa extension near the C-terminal prior between the GPI anchor (G:324 aa) and the three immunoglobulin-like domains (Additional file 2: Fig. S14B). In contrast, the 58 nt novel exon within Tx2 encoded a PTC (TAG) only 35 nt distant to the final exon junction complex, suggesting it might not trigger NMD. Truncation of the protein at this position (313 + 7 novel aa) would remove the GPI anchor potentially creating a near complete protein (320 aa) that is unable to attach to the cell membrane (Additional file 2: Fig. S14C).

#### Brain region enriched expression of novel isoforms

Many isoforms have brain region enriched or specific expression [51, 52]. Our amplicon sequencing approach identifies the presence and relative expression proportion of different isoforms. We next asked if any novel risk gene isoforms showed expression differences between brain regions. Overall, cerebellum exhibited the most differences in isoform expression, consistent with previous whole transcriptome results [12].

**Fig. 8:**
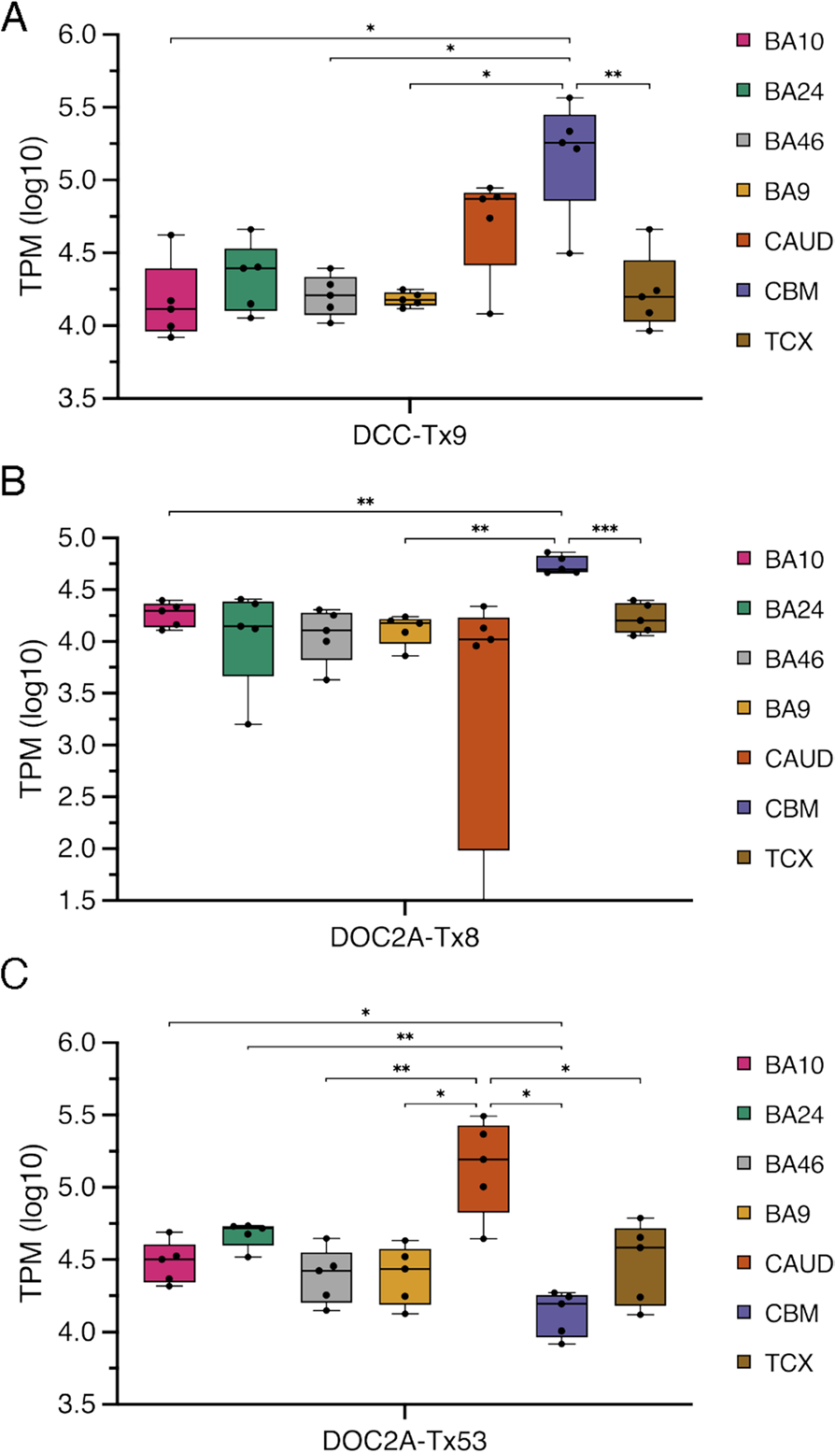
Brain region enriched expression of novel isoforms. **A** *DCC* novel transcript 9 had significantly higher TPM in CBM. ANOVA: *F* = 9.825, DF = 34. **B**, **C** *DOC2A* novel transcripts. Tx8 (**B**) had significantly higher TPM in CBM. One caudate sample recorded no expression and has been truncated from the plot. ANOVA: *F* = 1.893, DF = 34. Tx53 (**C**) had significantly higher TPM in caudate. ANOVA: *F* = 21.03, DF = 34. Brodmann’s Area (BA), caudate (CAUD), cerebellum (CBM), and temporal cortex (TCX). Ordinary one-way ANOVA Tukey’s multiple comparison adjusted *P* value: ** = *P* ≤ 0.01, *** = *P* ≤ 0.001, **** = *P* ≤ 0.0001

Depression risk gene DCC netrin 1 receptor (*DCC*) novel isoform Tx9 had significantly higher TPM in cerebellum (Fig. 8A, Additional file 4: Fig. J). TPMs of Tx9 in CBM were approximately 10 × higher than the average for cortical regions and 3 × higher than in caudate. This isoform, classified as a COJ and predicted to encode a 1425 aa protein, accounted for ~ 5% of total *DCC* expression (Additional file 2: Fig. S15A). Tx9 uses an alternative 3’SS (−60 nt) in cassette exon 17, and the skipped nucleotides cover an extracellular region and fibronectin type-III domain (UniProt). The SZ risk gene double C2 domain alpha (*DOC2A*) had two novel isoforms with significant variation in brain-specific expression including Tx8 in cerebellum and Tx53 in caudate (Fig. 8BC, Additional file 2: Fig. S15B, Additional file 4: Fig. K). Novel Tx8 used a novel splice donor in the canonical 5’UTR exon 1 (GT, + 158 nt) and was predicted to encode a 400 aa protein unchanged from the canonical transcript. Tx53 was the only novel transcript that showed moderate but specific expression in caudate samples or any tissue other than cerebellum (Additional file 2: Fig. S15C). Tx53 extends the known isoform ENST00000574405 to the canonical stop and is predicted to encode a 400 aa protein. Overall, 28 novel isoforms in 11 risk genes were found to have variable expression amongst brain tissues supporting a role for these isoforms within specific brain regions or potentially in a subset of cells (Additional file 3).

##### Sequencing and validation of novel exons

Our amplicon sequencing approach detected a total of 28 novel exons in 13 MHD risk genes. Using RT-PCR followed by Sanger sequencing, we validated a set of 21/21 targeted novel exons in ten genes (Table 1). The SZ risk gene chloride voltage-gated channel 3 (*CLCN3*) contained four novel exons within six novel isoforms, and an example of PCR validation is shown in Additional file 2: Fig. S16A. Validated novel exon mean length was 99 nt, ranging from 41 nt (*CLCN3*) to 231 nt (*GRM3*). 16 (76%) of validated novel exons were classified as “poison exons” as they encoded a PTC (Additional file 2: Fig. S16B), although two of these poison exons, within *NEGR1* and *XRN2*, were < 50 nt from the final exon junction and therefore may not undergo NMD. The novel exon contained within Tx3 for *XRN2* had the second highest isoform expression for the gene, following the canonical transcript (ENST0000037191), with 4.7% of TPM. If translated, this transcript would omit an omega-N-methylarginine modification site (ARG:946) within a disordered region at the C-terminus (Additional file 2: Fig. S17, Additional file 4: Fig. AE).

**Table 1:** Neuropsychiatric disorder risk gene novel exon validation. Definitions:* Chr* chromosome,* nt* nucleotide,* ORF* open reading frame, *PTC *premature termination codon, *UTR *untranslated region

Gene	Novel exon	Chr	Genomic coordinates		Size (nt)	Classification	UniProt/Pfam domain
			*Start*	*End*			
*SORCS3*	20a	10	105,244,778	105,244,837	60	ORF	Lumenal
*GRIA1*	2a	5	153,509,750	153,509,806	57	PTC	Extracellular
*XRN2*	16a	20	21,345,834	21,345,887	54	PTC	None
*XRN2*	29a	20	21,387,308	21,387,371	64	PTC (< 50nt)	Disordered
*SLC30A9*	6a	4	42,028,140	42,028,260	121	PTC	Helix
*SLC30A9*	9a	4	42,059,964	42,060,036	73	PTC	Cation efflux family
*GRM3*	2a	7	86,776,784	86,777,014	231	PTC	None
*GRM3*	3a	7	86,832,296	86,832,411	116	PTC	None
*NEGR1*	6a	1	71,532,866	71,532,907	42	ORF	None
*NEGR1*	6b	1	71,587,343	71,587,400	58	PTC (< 50nt)	Transmembrane
*RFTN2*	1a	2	197,654,257	197,654,391	135	PTC	None
*RFTN2*	1b	2	197,671,542	197,671,664	123	PTC	None
*RFTN2*	6a	2	197,616,952	197,617,073	122	PTC	None
*CNTN4*	1a	3	2,656,485	2,656,587	103	PTC	None
*PTK2B*	3a	8	27,318,676	27,318,793	118	UTR	None
*PTK2B*	3a (short)	8	27,318,696	27,318,793	98	UTR	None
*PTK2B*	3b	8	27,319,918	27,319,985	68	UTR	None
*CLCN3*	1a	4	169,630,165	169,630,335	171	UTR	None
*CLCN3*	2a	4	169,638,597	169,638,742	146	PTC	Cytoplasmic
*CLCN3*	2b	4	169,640,168	169,640,208	41	PTC	Cytoplasmic
*CLCN3*	2c	4	169,663,527	169,663,617	91	PTC	Cytoplasmic

Three novel exons were in untranslated regions and two were predicted to retain the ORF, including the 42 nt exon in *NEGR1* mentioned previously and a 60 nt exon within *SORCS3*. The novel exon in Tx1, encoding 20 aa (AMCGRAQWFTPVILALWETE), falls within the *SORCS3* lumenal region (position: LYS:956/PRO:957). Comparison of protein prediction models of the canonical (ENST00000369701.8) and novel isoform (Tx1) showed the addition of an unstructured loop with a partial alpha helix within the second polycystic kidney disease (PKD2) domain (Additional file 2: Fig. S18A–D), though the prediction confidence was low, so the structural impact on the PKD2 domain remains uncertain [53].

## Discussion

In this study, we used long-read sequencing to profile 31 candidate neuropsychiatric disorder risk genes identifying 363 novel RNA isoforms. We also present a new bioinformatic tool, IsoLamp, that can accurately identify and quantify novel RNA isoforms from long-read amplicon data. The recent proliferation of GWAS studies examining increasingly large population-wide data has identified hundreds of genomic variants associated with the risk of developing a mental health disorder [54, 55]. Evidence suggests that some risk variants, specifically those that are non-coding, play a role in pre-mRNA splicing, and our current understanding of the transcriptomic profile for these risk genes is limited [36, 55]. A key finding of our research is both the high number of novel expressed RNA isoforms and, for some candidate genes, the high expression of novel isoforms both individually and collectively. This finding reflects both the known complexity of alternative splicing in the human brain [56] and the current incompleteness of the reference transcriptome. As a result of the relatively deep sequencing afforded by this long-read approach, we have shown that there is a much higher level of RNA isoform diversity for these genes than reported in the current reference annotations. These findings provide new insight into the repertoire of RNA isoforms expressed in brain that could be important for understanding the risk and onset of neuropsychiatric disorders.

### RNA isoform discovery, classification and visualization

We generated a set of high-confidence RNA isoforms from nanopore long-read data using IsoLamp. IsoLamp optimizes and streamlines transcript isoform identification, quantification, and annotation from long-read amplicon data and outperformed other methods. IsoLamp improves upon our previously published pipeline TAQLoRe [36] by simplifying installation and use; the inclusion of optimized and tuneable isoform discovery parameters; the ability to down-sample reads to normalize for read depth differences between samples; primer position filtering to remove off-target or incomplete isoforms; and improved output files for downstream analysis. Our overall approach also overcomes the significant challenge of re-assembling and classifying RNA isoforms using short reads [57–59]. The primary outputs from IsoLamp, filtered transcripts (GTF) and transcript expression (TPM) are designed to be compatible with multiple downstream tools, including our visualization tool IsoVis to aid with interpretation and validation methods (https://isomix.org/isovis) [45].

Taken together, our benchmarking results highlight that IsoLamp’s optimized isoform discovery parameters and filters yield significant improvements in both precision and recall compared to Bambu, FLAIR, FLAMES and StringTie2. The expression filter applied to the data presented in this study is conservative, and for long and complex genes, may need to be tested to yield a balance of novel isoform detection and acceptable expression levels. IsoLamp also output consistent expression quantification that was robust to the quality of the annotations provided.

### Novel RNA isoforms in candidate neuropsychiatric disorder risk genes

The results presented in this study confirm our current limited understanding of RNA isoform profiles in the human brain and demonstrate how long-read sequencing is a powerful tool to address this issue [34, 36, 60].

We identified several highly abundant novel isoforms, including one, *ATG13* (Tx26), that was the most abundant gene isoform. *ATG13* forms part of a protein complex, including ULK1 and FIP200, that is critical for autophagy [61]. In our samples, transcript 26 had the highest TPM and contained a known skipping event of canonical (ENST000006683050) exon 12 which may be involved with FIP200 binding and the subsequent function of ATG13 [62]. Where Tx26 and several other novel isoforms differ from known (e.g., ENST00000359513) isoforms is in the 5’UTR, indicating this region may play a role in translation regulation [63]. Similarly, the SZ risk gene *CSMD1* had relatively high expression of novel isoforms. Recent evidence suggests enrichment of CSMD1 protein in the brain and activity as an inhibitor of the complement pathway in neurons [64]. Baum et al. [64] show that CSMD1 localizes to synapses and that loss of CSMD1 can lead to increased complement deposition potentially disrupting complement-associated synaptic pruning. The novel RNA isoforms identified in our samples provide transcriptional pathways through which *CSMD1* may be altered, potentially reducing expression or function of the protein, for example through incorporation of a premature termination codon (Tx33). Evidence from Alzheimer’s disease studies has also linked increased complement pathway activity to cognitive impairment. However, further studies, particularly in human models of neuronal development, will be needed to link *CSMD1* transcriptional variability to SZ risk and severity [64, 65].

Several novel isoforms and exons were identified for the ion channels *CLCN3*, *CACNA1C,* and *SLC30A9* which have shared risk for SZ, BPD, and ASD [21, 23, 54]. Voltage-gated ion channels are widely distributed in the brain and regulate neuronal firing. Mutations to these genes have been associated with disease and the emerging role of these channels in neuropsychiatric disorders has been previously reviewed [66]. *CLCN3* belongs to the CLC family of anion channels and transporters and has an established role in human neurodevelopment [67, 68]. We identified and validated four novel exons in *CLCN3*, three of which were predicted to encode a PTC which could lead to NMD. The fourth was located within the 5’UTR, an area known to impact translation regulation in humans, potentially through structural interference with the ribosome [63]. Splice variants of *CLCNC3* have been shown to impact intracellular localization and our results identified additional splice variants, in particular a novel RNA isoform (Tx9) which is similar to ENST0000613795, but includes a 76 bp exon 12 [67]. Twenty-two novel isoforms were identified for the calcium channel *CACNA1C.* Consistent with previous findings [36], the top ten novel isoforms, ranked by TPM, were classified as frame retaining, supporting their potential to generate functional proteins. *SLC30A9* (first known as *HUEL*) encodes the zinc transporter protein 9 (ZnT9), which is involved in zinc transport and homeostasis in the endoplasmic reticulum [69]. While the function of the protein is not fully understood, a 3 nt familial deletion (c.1047_1049delGCA) in the highly conserved cation efflux domain (CED) has been recorded to result in changes to protein structure, intracellular zinc levels and intellectual disability [70, 71]. Critically, we identified and validated a novel exon (Tx3-9a:73 nt) within this CED providing evidence that this region may be alternatively spliced more commonly than previously understood [69].

Finally, using mass spectrophotometry, we confirmed novel skipping events, including in ITIH4, indicating the utility of combining long-read sequencing with proteomics. ITIH4 is an acute-phase protein the serum levels of which have been associated with MDD and is thought to be involved in neuro-inflammation [72, 73]. Despite several studies outlining an association for *ITIH4* and risk for SZ, BPD, or MDD onset, the causal mechanisms for this gene remain elusive, and further study is required to further explore the impact of the coding change detected in our study.

### Limitations and future directions

Long-read amplicon sequencing, while providing an extremely sensitive isoform quantification method, is limited by the set(s) of primers used to amplify each risk gene. Our method aimed to locate primers within the 5’ and 3’ UTRs in proximity to the canonical translational start and stop codons. This approach generally amplified the entire coding sequence but does not capture full-length isoforms or variation in the UTRs, and additional primers must be made to capture alternative unique start and termination sites. When using this method, users must interpret the reported novel isoform proportions in the context of the known isoforms targeted, e.g., if the canonical isoform is not a target of the primer pair, novel isoform expression may appear inflated.

The results of our study are limited by the sample size of available control post-mortem brain tissue. The nanopore long-read data for each risk gene was generated from five elderly, male control individuals, with a single female sample removed from further analyses due to low RNA quality. The small number of available individuals means this dataset was not powered to investigate genotype impacts on isoform expression, though this will be an important area of investigation to determine which risk genotypes act through changes in isoform structure and/or expression.

Sample and RNA quality, as measured by RINe, is critical to high-quality sequencing, and this is especially true for long reads [36, 74]. Supporting previous findings in mRNA, our data suggest that pH values < 6.3 impacted the quality of post-mortem human brain RNA, which is especially critical for robust amplification of longer (> 5 kb) CDSs [75]. In addition, PCR cycling was kept as low as possible to avoid PCR bias towards shorter isoforms and other artifacts. However, we note that lower RINs, as recorded for Ind04, appeared to impact amplicons of longer CDS. To help overcome such issues, future long-read amplicon sequencing could incorporate unique molecular identifiers to tag molecules prior to PCR to ensure an accurate representation of the original RNA isoforms [76].

The candidate risk genes profiled in this study were selected based on multiple levels of evidence for their involvement in risk, not only from GWAS but from meta-analyses and further independent studies [22, 25]. While this approach was expected to produce a set of genes with high confidence of their involvement in disorder risk, it is not exhaustive and it will be important to ensure risk gene lists are updated as more evidence from GWAS and other studies becomes available [25, 55]. Recent evidence also supports the identification of genes linked to resistance against neuropsychiatric disease, and investigating gene and isoform expression of these may offer valuable insights into disease risk and progression [77]. In future, combining whole or amplicon transcriptomic data, large-scale proteomic data and machine-learning predictive models like TRIFID could help to identify and prioritize functional proteomic isoforms [78].

## Conclusions

In conclusion, we identified hundreds of unreported RNA isoforms, many of which could impact the function of neuropsychiatric risk genes, which also play crucial roles in normal neuronal development and activity. An understanding of the regulatory and functional impacts of these novel isoforms and their incorporation into existing transcript repositories will help form an important knowledge base of alternative splicing in the human brain [79, 80]. Some novel isoforms or exons may also be future therapeutic targets through the modulation of splice isoforms using antisense oligonucleotides or CRISPR technology.

## Methods

### Sample preparation and QC

Healthy control post-mortem human brain samples were obtained from six individuals collected through the Victorian Brain Bank (VBB) under HREC approvals #12,457 and #28,304. Age, sex, and additional details including the post-mortem interval (PMI), pH, and tissue weight are shown in Additional file 1: Table S1. Briefly, samples comprised 5 Males and 1 female, age range 51–72 years, PMI range 31–64 h, and pH range 5.7–6.7. Due to low RNA integrity number equivalent (RINe), the female control was removed from further analysis. Frozen tissue (weight range 57–135 mg) was cut from seven brain regions including Brodmann areas (BA), BA9 (dorsolateral prefrontal cortex (DLPFC)), BA46 (medial prefrontal cortex (MPFC)), BA10 (fronto-parietal cortex (FPC)), Brodmann Area 24 (dorsal anterior cingulate cortex (dACC)), caudate, cerebellum, and temporal cortex. Total RNA was extracted from bulk tissue in eight randomized batches of 3–6 samples. First, frozen brain tissue was homogenized on ice, using a manual tissue grinder (Potter–Elvehjem, PTFE), while immersed in 1 mL QIAzol Lysis Reagent (QIAGEN). Lysate was then processed using a RNeasy Lipid Tissue Kit (QIAGEN, 74,804), according to the manufacturers’ instructions. Isolated RNA quality and quantity was checked using a Qubit 4 Fluorometer (2 µL), TapeStation 4200 (RINe: cut-off = 6), and Nanodrop 2000.

### Database curation and candidate risk gene selection

MHD risk genes were selected for long-read amplicon sequencing using an internal database that aimed to collate evidence from the literature of gene involvement in disease risk. Lines of evidence included the following: GWAS; meta-analyses including MAGMA (and variants including eMAGMA, hMAGMA, nMAGMA); TWAS; SMR; and follow-up studies including fine mapping, protein–protein interaction (PPI), epigenetic (DNA methylation), and targeted experimental validation (Additional file 2: Fig. S5).

The foundation of this database was a list of significant GWAS SNPs for SZ, BD, MDD, and ASD. Association data was downloaded from the NHGRI-EBI GWAS Catalog [81]. MHD GWAS associations were filtered on the “Disease/Trait” column to exclude effects of treatments including pharmaceutical, mixed disorder studies and associations with behavioral traits like smoking or alcohol intake. Associations were excluded if both the “reported gene” column was “not reported (NR)” and the “mapped gene” column was blank. Date data was downloaded, filters applied, and percentage associations retained are detailed in Additional file 1: Table S4.

Follow-up studies and experiments were then identified in the literature, and the reported genes were manually collated and assigned to an “evidence” category (i.e. MAGMA, SMR, experimental validation). For each entry, the PubMed ID of the reporting manuscript, the first author, and the reported SNP and gene were recorded. A custom R script was then used to summarize the database and identify the number of unique lines of evidence reporting each gene as a candidate risk gene (Additional file 5). Candidate risk genes were then sorted by evidence (high to low), separately for each MHD. A multi-trait evidence list was also made by combining each MHD table together and again sorting by descending evidence. This gave us flexibility to focus on risk genes that appeared to be specific to a single MHD or those with shared risk across disorders.

### Primer design, cDNA synthesis and long-range PCR

Thirty-one (31) MHD risk genes were selected from our database and the full coding sequence (CDS) from the canonical isoform was downloaded from the UCSC Genome Browser [82]. Primers, located in the 5’ and 3’ UTRs, were designed to amplify the CDS using Primer3 Plus [83]. Additional primers were Made for 13 genes to amplify alternative start or end sites that were not captured by a single primer pair. Additional UCSC track sources including expressed sequence tags (EST), transcript support level (TSL), APPRIS designation, human mRNA support, cap-analysis of gene expression (CAGE) peaks, CpG islands, and H3K4Me3 marks were examined to ensure there was enough evidence that alternative start or end sites were real before a primer was designed [82]. All primers, primer combinations and modified Primer3 Plus settings are Listed in Additional file 1: Table S3. Risk gene primers from Primer3 Plus were aligned to tracks on the UCSC Genome Browser using BLAT for visualization and tested using the In-Silico PCR [82].

To amplify risk gene CDSs, 1 µg of total RNA was used as a template for cDNA synthesis using Maxima H Minus Reverse Transcriptase (Thermo Fisher Scientific, EP0752, 200 U/µL) according to the manufacturers’ instructions. Two duplicate cDNA plates were generated simultaneously to reduce variability and provide enough template for multiple risk gene PCRs. Risk genes were amplified using one of the following DNA polymerases: LongAmp® Taq 2X Master Mix (NEB, M0287S), Platinum™ SuperFi II PCR Master Mix (Thermo Fisher Scientific, 12,368,010) or PrimeSTAR GXL (TakaraBio, R050B). LongAmp® Taq was tested first by default; however, if it performed poorly, an alternative polymerase was tested and optimized to keep the number of PCR cycles to a minimum. Each set of gene primers was individually optimized by adjusting PCR cycling conditions (Additional file 1: Table S3) until sufficient pure template (~ 1–10 ng) could be produced for input to barcoding. Short fragments and primer-dimer were removed prior to barcoding using AMPure XP beads (Beckmann Coulter) at 0.5–0.8 × ratios. An overview of the experimental protocol is shown in Fig. 1A. A detailed user protocol titled “A guide to long-range PCR for Nanopore sequencing (v2)” is available on protocols.io [84].

### Long-read amplicon sequencing

Barcoding conditions for sample multiplexing (*N* = 35, EXP-PBC096, ONT) and library preparation for long-read sequencing followed the recommended ligation sequencing protocol (Fig. 1A) (SQK-LSK109/110, ONT). All barcoding PCR was done using LongAmp® Taq 2X Master Mix with an amplicon specific extension time (approximately 1 min/kb) and 10–15 × cycles. AMPure clean-up following adaptor Ligation was adjusted from the default ratio of 0.4 × depending on the length of the target amplicon. Adaptor ligated libraries were loaded (25–35 fmol) onto MinION (FLO-MIN106) flow cells, and a minimum of 10,000 reads per sample were targeted before flushing and storing the flow cell. All runs were re-basecalled using the super-accurate (SUP) basecalling model (Guppy v6.0.17, 2022) and minimum qscore = 10.

### Isoform discovery from long-read amplicon sequencing with IsoLamp

We developed a new bioinformatic pipeline, IsoLamp [38], for the analysis of long-read amplicon data (Fig. 1B). First, pass reads were downsampled [85] to a consistent number (8000) (default: 10,000) per barcode and mapped to the reference genome with minimap2 (v2.24) [39]. Then, low accuracy reads (≥ 5% error rate) were removed, and samples were merged prior to isoform identification. Read accuracy was calculated using CIGAR strings in the BAM files and is defined as (‘X’ + ’ = ’ + ’I’ + ’D’- ‘NM’)/(‘X’ + ’ = ’ + ’I’ + ’D’). Next, the merged BAM file of high accuracy reads was used as input for isoform discovery with Bambu (v3.2.4) using the following parameters: novel discovery rate (NDR) = 1 and min.fractionByGene = 0.001 [40]. Next, isoforms identified with Bambu were filtered to remove any with zero expression and to retain only isoforms overlapping the known primer coordinates using bedtools intersect (v2.30) [86]. Reads from each barcode were then quantified with salmon in alignment-based mode (v0.14.1) [87]. An expression filter can be applied at this stage by setting two optional, user definable parameters: *TPM_minimum* (minimum expression level for an isoform) and *samples_minimum* (proportion of samples an isoform must meet the TPM minimum threshold in). The default values based on SIRV optimization are 5000 TPM and 25% of samples, respectively. The application of this filter to our brain samples required isoforms to meet a threshold of 5000 TPM in a minimum of 8 brain samples. Identified isoforms were then used to create an updated transcriptome with GffRead (v0.12.7) [88] and annotated with GffCompare (v0.12.6) [88]. The pipeline outputs a list of isoform annotations (.gtf), isoform expression as transcripts per million (TPM) and proportion of overall gene expression, as well as a report summarizing the results. If the user specifies a grouping variable for their input samples, a *t*-test is performed between isoform proportions between groups and a false discovery rate of 0.05 is applied.

We benchmarked the performance of the IsoLamp pipeline using Spike-in RNA Variant (SIRV) Set 1 synthetic RNA controls (Lexogen). SIRV isoforms are present in three mixes (E0, E1, E2) that contain each isoform in varying known concentrations. Primers were designed to amplify from the first to the last exon (as described above) of the SIRV5 and SIRV6 genes from cDNA generated in triplicate from each SIRV mix (*N* = 27) (Additional file 2: Fig. S1). PCR amplification conditions for SIRV amplicons are shown in Additional file 1: Table S5. Samples were barcoded and sequenced as described above, and subsequent base calling and demultiplexing were performed with Guppy (v6.0.17, SUP, 2022). The IsoLamp pipeline was compared against four other isoform discovery tools: StringTie2 [43], FLAIR [41], FLAMES [42] and Bambu [40] (using both their default parameters and the optimized parameters used in IsoLamp). The sensitivity, specificity, and quantification accuracy (based on the correlation between the expected versus observed counts) of the programs were compared using three SIRV reference annotations: Complete (C), Incomplete (I) (missing isoforms, to test ability to recover unannotated true positive isoforms) and Over (O) (annotation contains extra isoforms not present in mixes, to test ability to minimize false positive annotated isoforms). These references were downloaded from Lexogen under “Additional annotations” (https://www.lexogen.com/sirvs/download/). Specific information on SIRV isoform annotation for each gene can be found in the SIRV Set 1 user guide (pp. 25–28). Briefly, there are 69 SIRV isoforms in the Complete reference (SIRV_C), 25 (36.2%) of these are removed from the Insufficient reference (*N* = 44, SIRV_I) and 31 (44.9%) are added to the Over-annotated reference (*N* = 100, SIRV_O). Additionally, we removed one extra SIRV (502) from the insufficient annotation to ensure multiple amplified SIRV5 isoforms were missing from the annotation and to increase the stringency of our benchmarking. Novel isoforms were categorized using SQANTI3 against the human reference (GENCODE release 41, GRCh38.p13). Finally, a combined dataset of expression values for each known and novel isoform and its associated metadata (Additional file 1: Table S6) including brain region, gene, RINe, pH, individual, PMI, and age was analyzed using principal component analysis (PCA) in R (Additional file 6).

### Novel exon validation

Nanopore long-read supported novel exons were validated by RT-PCR. Amplification was initially tested in a single sample shown by nanopore sequencing to express the novel exon (13 samples across 6 brain regions were used). Successful amplification was confirmed by Sanger sequencing. For exons which required PCR optimization, a pool of 4 post-mortem brain samples were utilized, followed by Sanger sequencing. Amplicons were designed from the known 5’ flanking exon into the novel exon and from the novel exon into the known 3’ flanking exon. An amplicon spanning the known 5’ and 3’ flanking exons was used as a positive control. Primers were designed using Primer3 [83] and checked using Primer BLAST [89] and are Listed in Additional file 1: Table S7. In some cases, the primer design space was restricted by the novel exon sequence length and/or nucleotide composition. Novel exons were amplified using *Taq* 2X MasterMix (NEB, M0270L), and cycling conditions can be found in Additional file 1: Table S7. PCR products were visualized via gel electrophoresis using GelGreenⓇ Nucleic Acid Stain (Biotium, 41,005) and GeneRuler 100 bp ladder (TFS, SMN0243). PCR products in the expected size range were cleaned up using AMPure XP Reagent (Beckmann Coulter, A63881) at a 1.8 × ratio to remove fragments < 100 bp and sent for Sanger sequencing (100–200 bp, AGRF).

### Protein isolation and novel sequence detection using mass spectrophotometry (MS)

Two Mass spectrophotometry techniques, targeted and untargeted with fractionation, were used to analyze bulk, post-mortem Human brain tissue from 7 individuals with no known neurological or neuropsychiatric conditions. Brain regions included frontal cortex (BA46), cerebellum, and caudate (*N* = 4 targeted, *N* = 9 untargeted). Sample mean age = 68.3 (51.6–81.2 years), mean PMI = 36.2 (22–64 h), and mean weight = 59.3 (37–102 mg) (Additional file 1: Table S8). Samples were lysed in 500 µL of guanidinium-HCl buffer using tip-probe sonication, heated briefly to 95 ℃ and diluted 1:1 with LC–MS water before 4 mL of ice-cold acetone was added to precipitate protein overnight at − 30 ℃. Following a wash (3 mL 80% cold acetone) and incubation (− 30 ℃, 1 h), supernatant was discarded and protein air-dried (RT, 30 min). The protein pellet was resuspended in 500 µL 10% TFE in 100 mM HEPES (pH 7.5) and sonicated. Protein concentration was estimated with BCA (1 µL sample + 9 µL 2% SDS). Normalized protein (10 µg/10 µL) was then digested using a combination of LysC/trypsin or GluC for all samples. Peptides were separated on a Dionex 3500 nanoHPLC, coupled to an Orbitrap Lumos mass spectrometer (Thermo Fisher Scientific) via electrospray ionization in positive mode with 1.9 kV at 275 °C and RF set to 30%. Separation was achieved on a 50 cm × 75 µm column packed with C18AQ (1.9 µm; Dr Maisch, Ammerbuch, Germany) (PepSep, Marslev, Denmark) over 120 min at a flow rate of 300 nL/min. The peptides were eluted over a Linear gradient of 3–40% Buffer B (Buffer A, 0.1% v/v formic acid; Buffer B, 80% v/v acetonitrile, 0.1% v/v FA), and the column was Maintained at 50 °C. The instrument was operated in targeted M2 acquisition mode with an MS1 spectrum acquired over the Mass range 300–1300 m/z (120,000 resolution, 100% automatic gain control (AGC) and 50 ms maximum injection time) followed by targeted MS/MS via HCD fragmentation with 0.7 m/z isolation (60,000 resolution, 200% AGC, 300 ms Maximum injection time and stepped normalized collision energy 25, 30, and 35 eV). Data were analyzed in Proteome Discover v2.5.0.400 with SequestHT [90] and searched against a custom.fasta database containing only risk gene predicted protein isoform sequences containing the targeted peptide sequences. Precursor Mass tolerance was set to 10 ppm and fragment Mass tolerance set to 0.02 Da. Data were filtered to 1% FDR at the peptide spectral match level only, i.e., no protein level FDR, and MS/MS annotations were Manually verified. To increase the depth of untargeted analysis, peptides were separated offline into 12 fractions. Briefly, 12 µg of peptide was injected onto a 15 cm × 0.3 mm column packed with 1.7 μm C18BEH particles (Waters) using a Dionex U3000 UHPLC. A gradient of 0–30% acetonitrile containing 10 mM ammonium formate pH 7.9 was delivered over 60 min at 5 µL/min. Peptides were detected at 210 nm, and 48 fractions were collected and automatically concatenated into 12 fractions. Peptides were dried by vacuum centrifugation and resuspended in 2% acetonitrile containing 0.1% trifluoracetic acid prior to LC–MS/MS analysis. MS proteomics data have been deposited to the ProteomeXchange Consortium via the PRIDE [91, 92] partner repository with the dataset identifier PXD063836 [91]. Protein structure prediction was performed using AlphaFold accessed through UCSF ChimeraX (v1.5) [51, 53, 93].

### Statistics

Statistical results presented in this manuscript included linear regression to investigate RNA quality (RIN) against individual, pH, and PMI. Ordinary one-way ANOVAs using Tukey’s multiple comparison correction were used to analyze isoform TPMs between brain regions. Statistical tests and associated graphical output were performed using GraphPad Prism 10.1.0.

### Isoform data visualization

The publicly available web-tool IsoVis (v1.6, https://isomix.org/isovis/) was used to visualize RNA isoforms and associated expression data [45]. Known and novel RNA isoforms are represented as a stack to compare alternative splicing events between different isoforms. Read counts assigned to each RNA isoform for each of the 35 samples were visualized as a heatmap.

## Supplementary Information

Additional file 1: Table S1. Case demographics and tissue information. Table S2. IsoLamp benchmarking summary statistics. Table S3. Primers, PCR conditions and additional details for risk gene amplicons. Table S4. GWAS Catalog meta-data and filtering conditions. Table S5. SIRV primers and PCR cycling conditions. Table S6. Sample metadata. Table S7. Novel exon validation. Genes, final PCR primers and additional details for validation. Table S8. Post mortem brain samples used for targeted and deep fractionation mass spectrophotometry (MS).

Additional file 2: Fig. S1. Experimental design of SIRV amplicon controls. Fig. S2. Benchmarking IsoLamp using spike-in SIRVs and the optimised IsoLamp expression-based filter. Fig. S3. Post-mortem human brain RNA QC. Fig. S4. Principal component analyses (PCA) of brain samples. Fig. S5: Mental health disorder (MHD) risk gene list curation pipeline. Fig. S6. Long-read amplicon mapping accuracy. Fig. S7. Filtering RNA isoforms using the *samples_minimum* parameter in IsoLamp. Fig. S8. Risk gene isoform counts. Fig. S9. Linear regression of amplicon length or canonical exon count against isoform count and novel isoform TPM proportion does not deviate significantly from zero. Fig. S10. Novel alternative splicing counts. Fig. S11. UCSC screenshot of *CACNA1C* splicing hotspot. Fig. S12. Screenshot peptide spectrum. Fig. S13. Confirmation of GABBR2 exon 5 skipping. Fig. S14. *NEGR1* splice isoforms and protein prediction. Fig. S15. A. Brain region enriched expression of novel isoforms. Fig. S16. Novel exon validation in *CLCN3*. Fig. S17. Splice graph of *XRN2* novel isoforms containing novel exons. Fig. S18. *SORCS3* novel exon and protein structure predictions.

Additional file 3. List of all known and novel isoforms and predicted impact on known protein domains, open reading frame (ORF), associated instability index, and notes.

Additional file 4. IsoVis (v1.6 (2024–02-29)) stack and heatmap output for each risk gene sorted by descending TPM. Figures A – Z, AA – AG.

Additional file 5. Custom R script to summarise GWAS database and identify the number of unique lines of evidence reporting each gene as a candidate risk gene.

Additional file 6. Custom R script for combining all risk gene TPM counts and performing PCA analyses. 

Additional file 7. Review history.

## Data Availability

All raw nanopore (ONT) long-read data (fastq) generated for each of the genes reported in this manuscript are available at the European Genome-Phenome Archive (EGA) study: EGAS00001007744 [94, 95]. A single GTF and TPM file containing all risk gene isoform and associated expression data from IsoLamp analysis and R scripts (Additional files 5 and 6) are freely available (MIT License) on Zenodo [95]. The IsoLamp pipeline is open source (MIT License) and freely available on GitHub (https://github.com/ClarkLaboratory/IsoLamp) and Zenodo [38]. IsoVis is open source (Mozilla Public License Version 2.0) and freely available at: https://isomix.org/isovis/ [45]. MS data are freely available (Creative Commons Public Domain) via ProteomeXchange: PXD063836 [91].
