# Supplementary material for: Long-read sequencing reveals the RNA isoform repertoire of neuropsychiatric risk genes in human brain
Source: Genome Biol. 2025 Sep 23;26:298. doi: 10.1186/s13059-025-03724-1 (PMC12455821; doi:10.1186/s13059-025-03724-1)
Supplement: Supplementary file 2 — Additional file 2: Fig. S1. Experimental design of SIRV amplicon controls. Fig. S2. Benchmarking IsoLamp using spike-in SIRVs and the optimised IsoLamp expression-based filter. Fig. S3. Post-mortem human brain RNA QC. Fig. S4. Principal component analyses (PCA) of brain samples. Fig. S5: Mental health disorder (MHD) risk gene list curation pipeline. Fig. S6. Long-read amplicon mapping accuracy. Fig. S7. Filtering RNA isoforms using the samples_minimum parameter in IsoLamp. Fig. S8. Risk gene isoform counts. Fig. S9. Linear regression of amplicon length or canonical exon count against isoform count and novel isoform TPM proportion does not deviate significantly from zero. Fig. S10. Novel alternative splicing counts. Fig. S11. UCSC screenshot of CACNA1C splicing hotspot. Fig. S12. Screenshot peptide spectrum. Fig. S13. Confirmation of GABBR2 exon 5 skipping. Fig. S14. NEGR1 splice isoforms and protein prediction. Fig. S15. A. Brain region enriched expression of novel isoforms. Fig. S16. Novel exon validation in CLCN3. Fig. S17. Splice graph of XRN2 novel isoforms containing novel exons. Fig. S18. SORCS3 novel exon and protein structure predictions. [file 13059_2025_3724_MOESM2_ESM.pdf]

Figure S1.

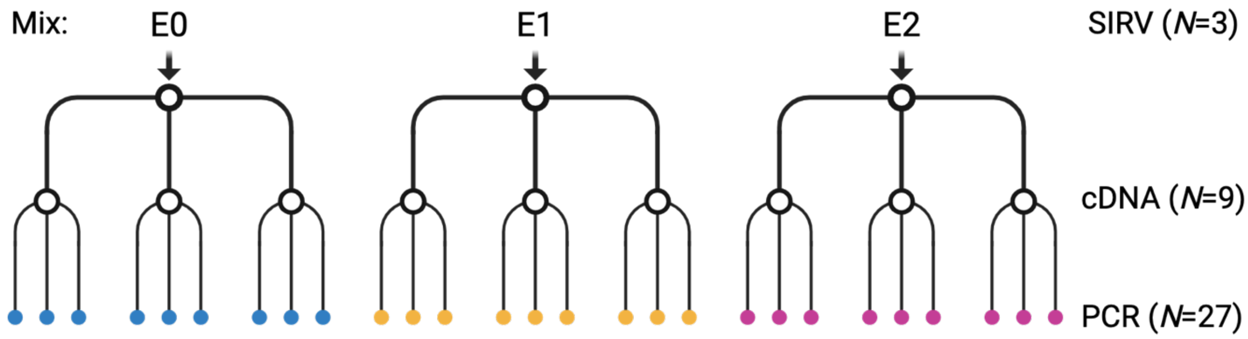

Fig. S1. Experimental design of SIRV amplicon controls. E0, E1 and E2 represent each SIRV mix of known isoform concentrations. Each mix was converted into cDNA in triplicate and finally, the full length of synthetic genes SIRV5 and 6 were amplified using PCR in triplicate for each cDNA replicate.

Figure S2.

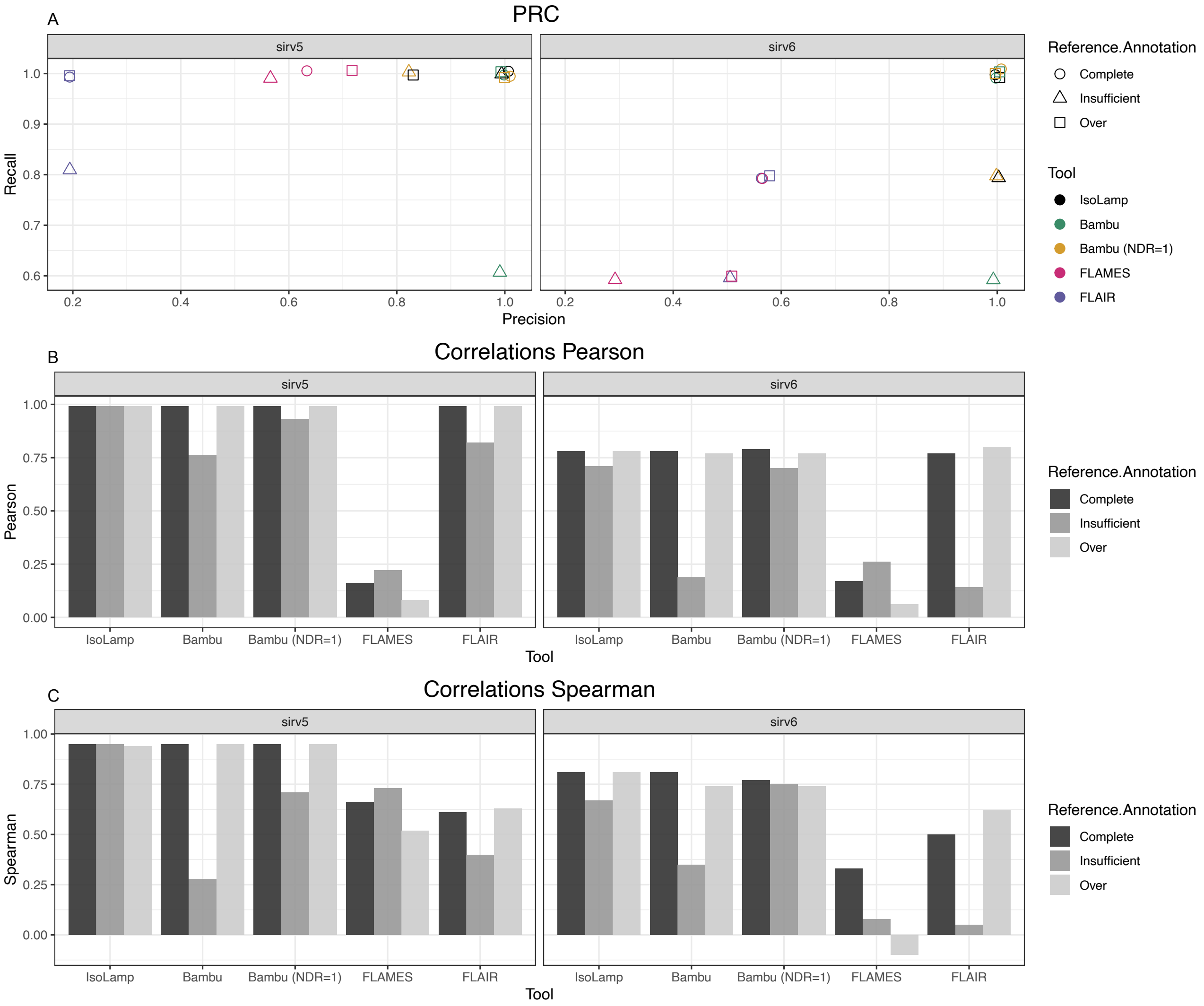

Fig. S2. Benchmarking IsoLamp using spike-in SIRVs and the optimised IsoLamp expression-based filter. A. Precision recall of each tested pipeline with the complete (N=69), insufficient (N=44) or over-annotated (N=100) SIRV reference, filtering all results using the IsoLamp expression-based filter. IsoLamp (black) returned high quality isoforms from amplicon data of both SIRV5 and 6. Pearson **(B)** and Spearman **(C)** correlations for each pipeline between known and observed expression values for SIRV 5 and 6 mixes.

Figure S3.

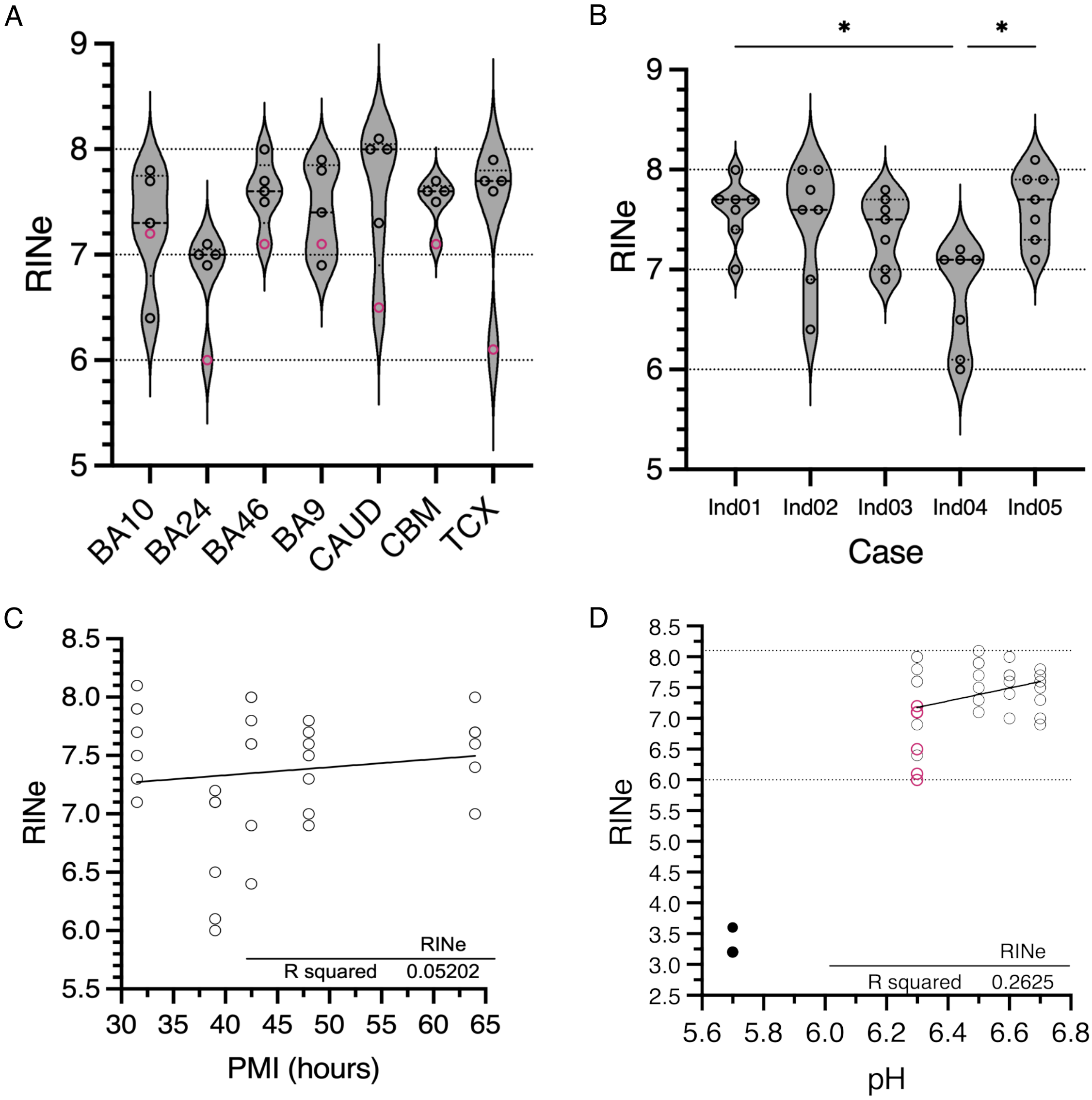

Fig. S3. Post-mortem human brain RNA QC. A. RNA integrity number equivalent (RINe) did not show any significant differences between brain regions. Pink circles indicate samples from Individual 04 (pH = 6.3) in A and D. **B.** RINe for brain tissue was generally between 7 – 8. RINe from individual (Ind) 04 was significantly lower when compared to individuals 01 (p=0.0259) and 05 (p=0.0433). One way ANOVA: F=6.224, DF=6. **C.** No correlation was detected between RNA quality (RINe) and individual post-mortem interval (PMI). **D.** Decreasing individual brain pH appears to impact RINe. Filled circles indicate degraded RNA isolated from individual 06 (female) which was excluded from further analysis. Data in B are staggered for clarity.

Figure S4.

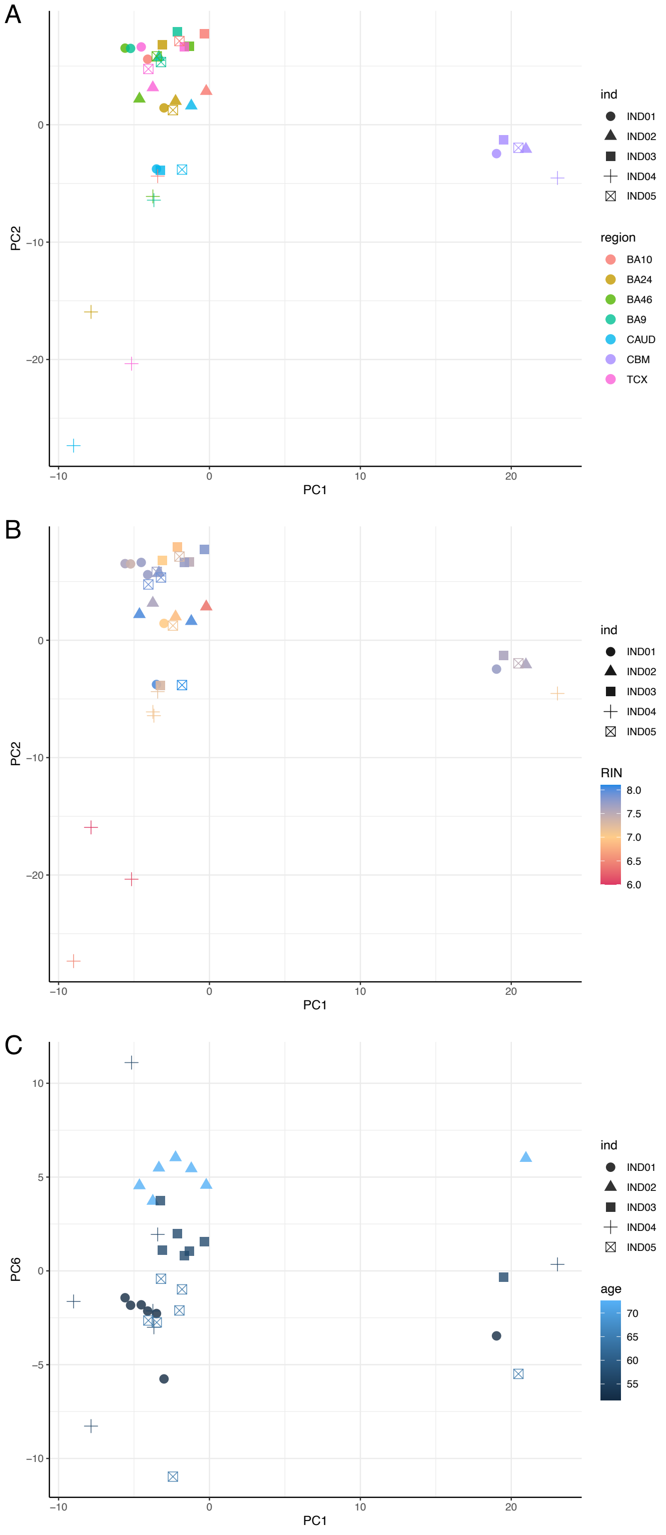

Fig. S4. Principal component analyses (PCA) of brain samples. PC1 and PC2 coloured by brain region **(A)** and RNA integrity (RIN) **(B)**. **C.** PC1 and PC6 coloured by donor age (years). Key: individual (IND), Brodmann's area (BA), caudate (caud), cerebellum (cbm) and temporal cortex (TCX).

Figure S5.

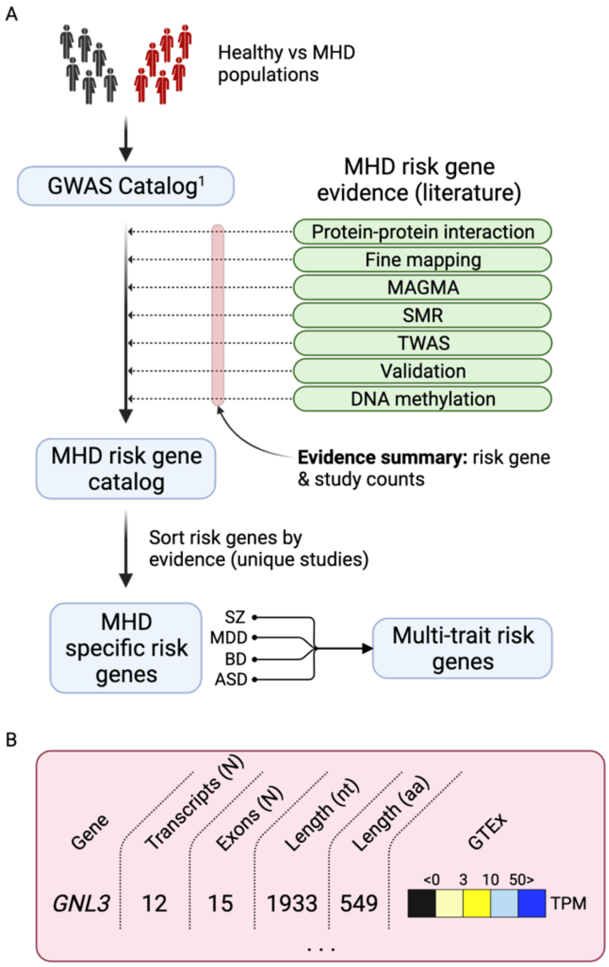

Fig. S5. Mental health disorder (MHD) risk gene list curation pipeline. A. Single nucleotide polymorphism (SNP) catalogues form the foundation of the final candidate risk gene evidence lists used to select genes for amplicon sequencing. These catalogues are collated from up-to-date, large-scale genome wide association studies (GWAS) of MHDs. A single GWAS catalogue was downloaded for schizophrenia (SZ), major depressive disorder (MDD), bipolar disorder (BD) and autism spectrum disorder (ASD), and associations were filtered according to criteria in Supplementary Table 4. GWAS data generally had either a mapped or reported gene associated with each SNP. Further evidence (i.e. reported genes) from categorised literature sources (shown in green) was then added to the list. This list was then sorted (high to low) by the number of occurrences of a risk gene in unique studies across all evidence/validation categories. No weighting was applied to any category. A multi-trait list was also made containing evidence for risk genes across all four MHDs so shared risk genes could be identified. **B.** An example of additional risk gene information included in the list e.g. for *GNL3*, count of known transcripts, count of coding exons for the canonical isoform, length in nucleotides (nt), length of protein in amino acids (aa) and the categorised Genotype-Tissue Expression (GTEx) in transcript per million (TPM) for each brain tissue [94]. *Definitions*: multi-marker analysis of Genomic annotation (MAGMA), summary-based Mendelian randomisation (SMR), transcriptome wide association study (TWAS).

Figure S6.

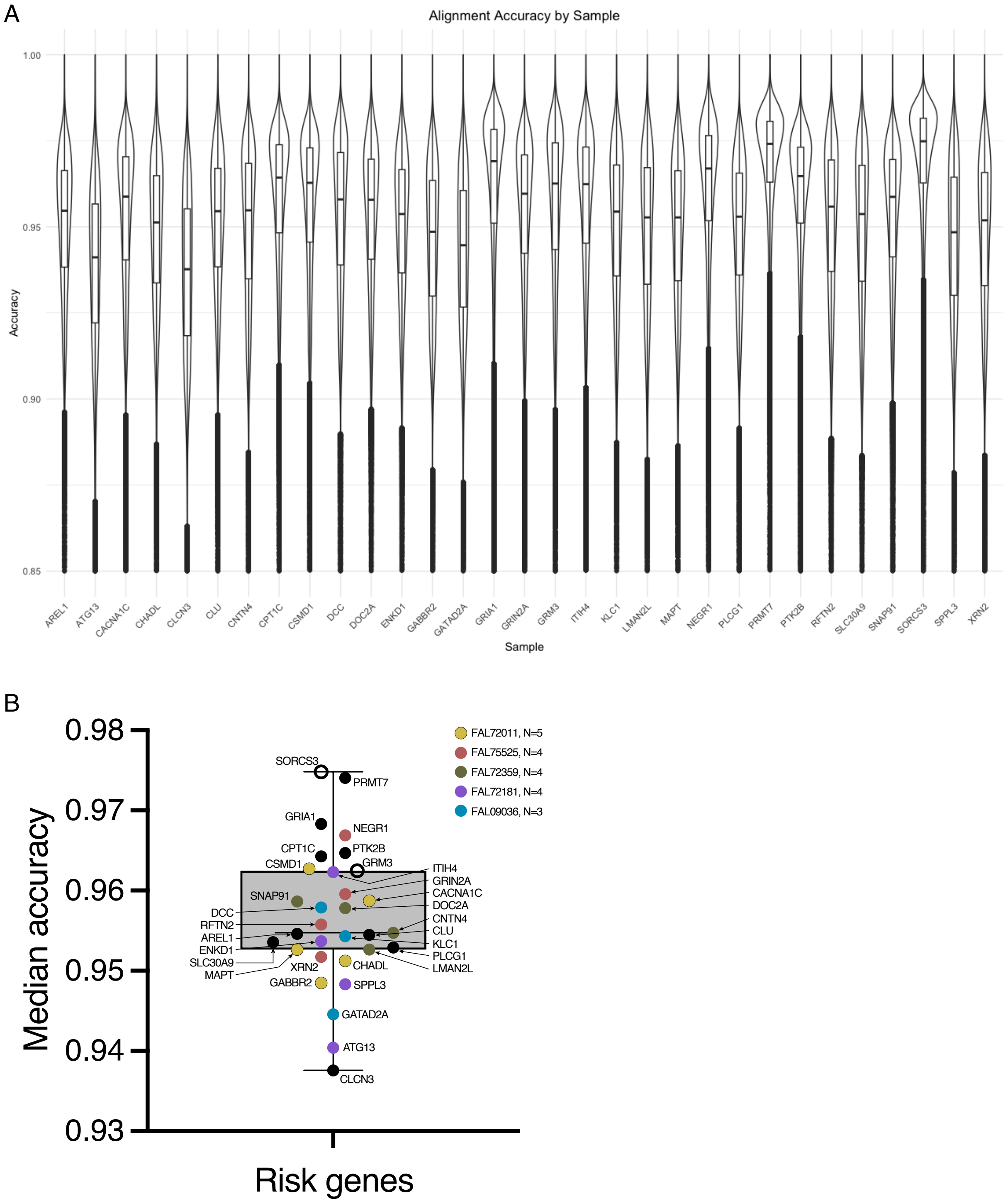

Fig. S6. Long-read amplicon mapping accuracy. A. All sequenced risk genes. Plotted range 0.85 – 1.00. **B.** A box and whiskers plot of the median accuracy for each risk gene. Open circles indicate the library was prepared with ligation sequencing kit 110 (ONT). Colours indicate flow cells (prefix: FAL) that were used for multiple long-read libraries (N=3-5).

Figure S7.

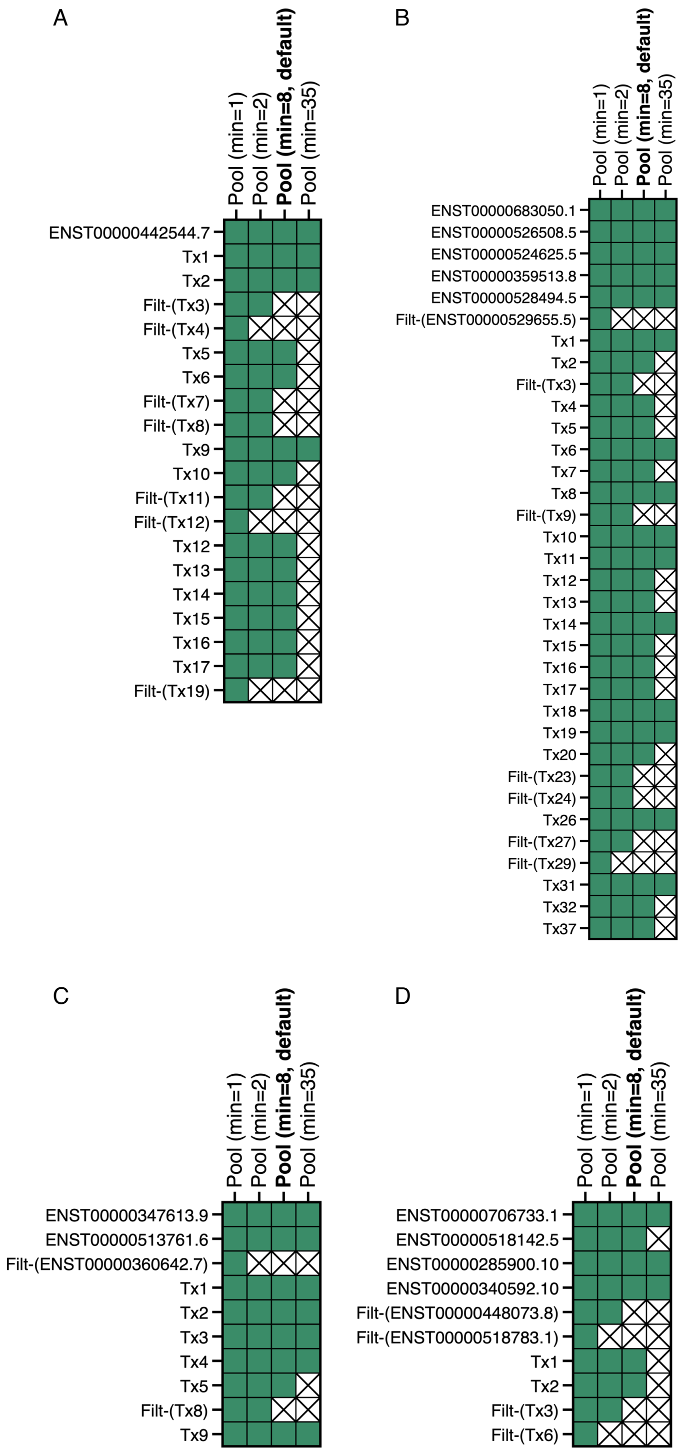

Fig. S7. Filtering RNA isoforms using the *samples_minimum* parameter in IsoLamp. Known and novel isoform filtering is shown for **A.** DCC, **B.** ATG13, **C.** GRIA1 and **D.** CLCN3. The isoforms reported for each of these genes in the manuscript were identified using the default value (25%, min=8, shown in bold) for this parameter. Stepwise filtering was done using the same subset of reads and the parameter set to 1, 2, 8 and 35 (100% of samples). The 'Filt-' notation denotes isoforms removed from the final set of isoforms reported in the manuscript. An isoform removed at min=2 indicates an isoform that was found in only 1 sample while isoforms removed at the min=8 filter indicates isoforms that were present in a low number of samples (N=2-7). The *TPM-minimum* parameter for all testing was set to 5000.

Figure S8.

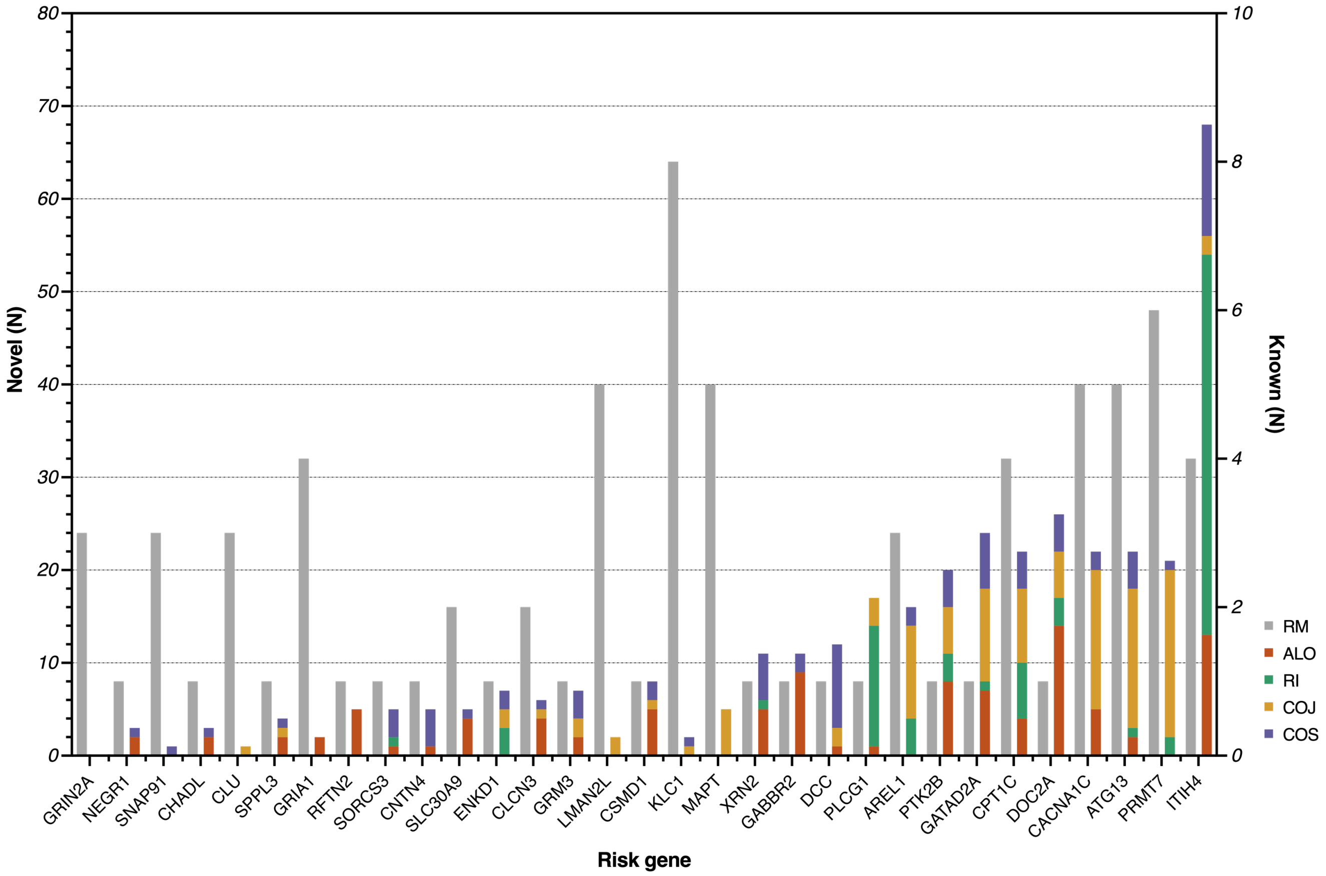

Fig. S8. Risk gene isoform counts. The number of detected isoforms (known and novel) is shown for each risk gene sorted from lowest (*GRIN2A*) to highest (*ITIH4*). Each isoform was classified into a SQANTI subcategory: reference match (RM), containing at least one novel splice site (ALO), retained intron (RI), combination of known junctions (COJ) or splice sites (COS). Novel isoform counts are plotted on the left Y-axis and known (RM) isoform counts are plotted on the right Y-axis.

Figure S9.

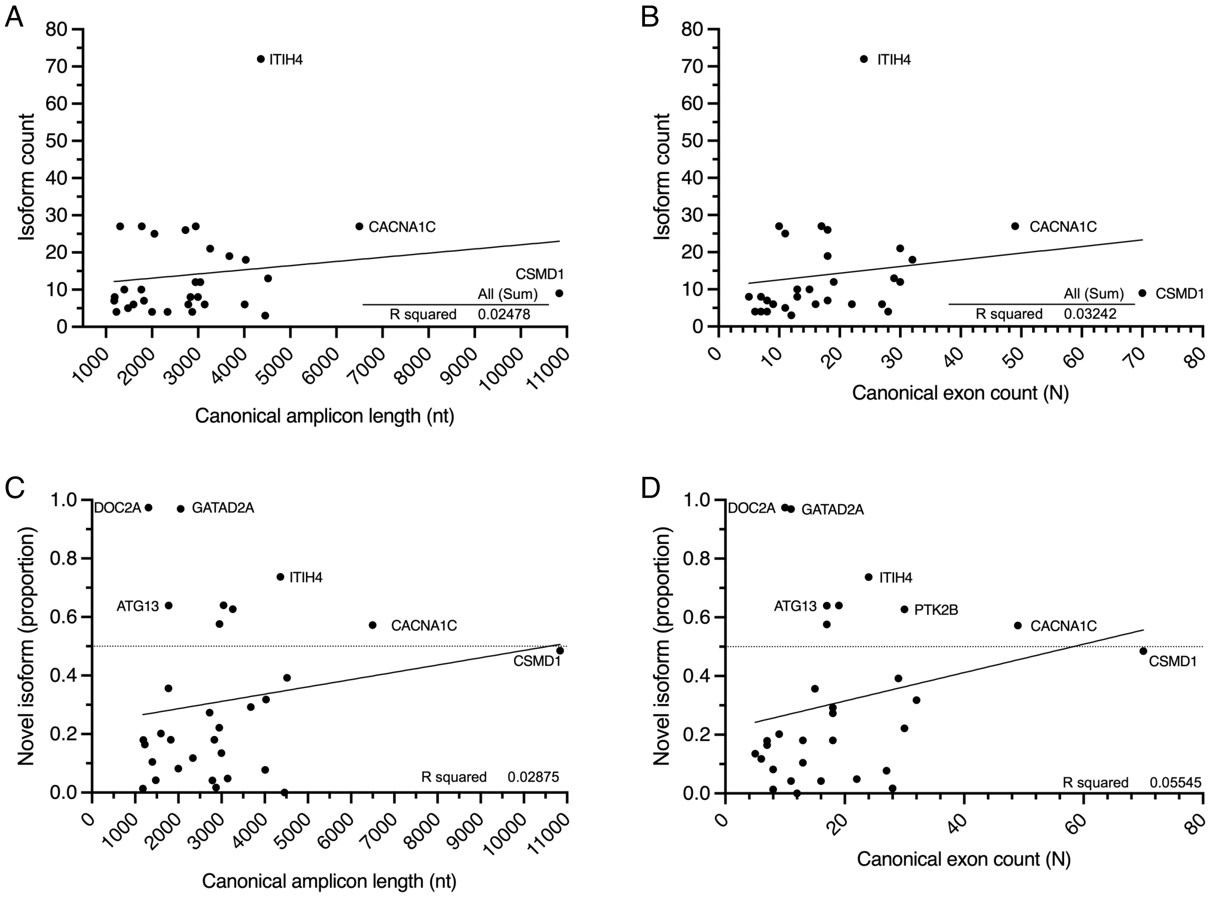

Fig. S9. Linear regression of amplicon length or canonical exon count against isoform count and novel isoform TPM proportion does not deviate significantly from zero. Linear regression of known and novel isoform counts with expected canonical amplicon length (**A**) and number of canonical exons **(B**). Linear regression of novel isoform read proportion with expected canonical amplicon length (**C**) and number of canonical exons (**D**).

Figure S10.

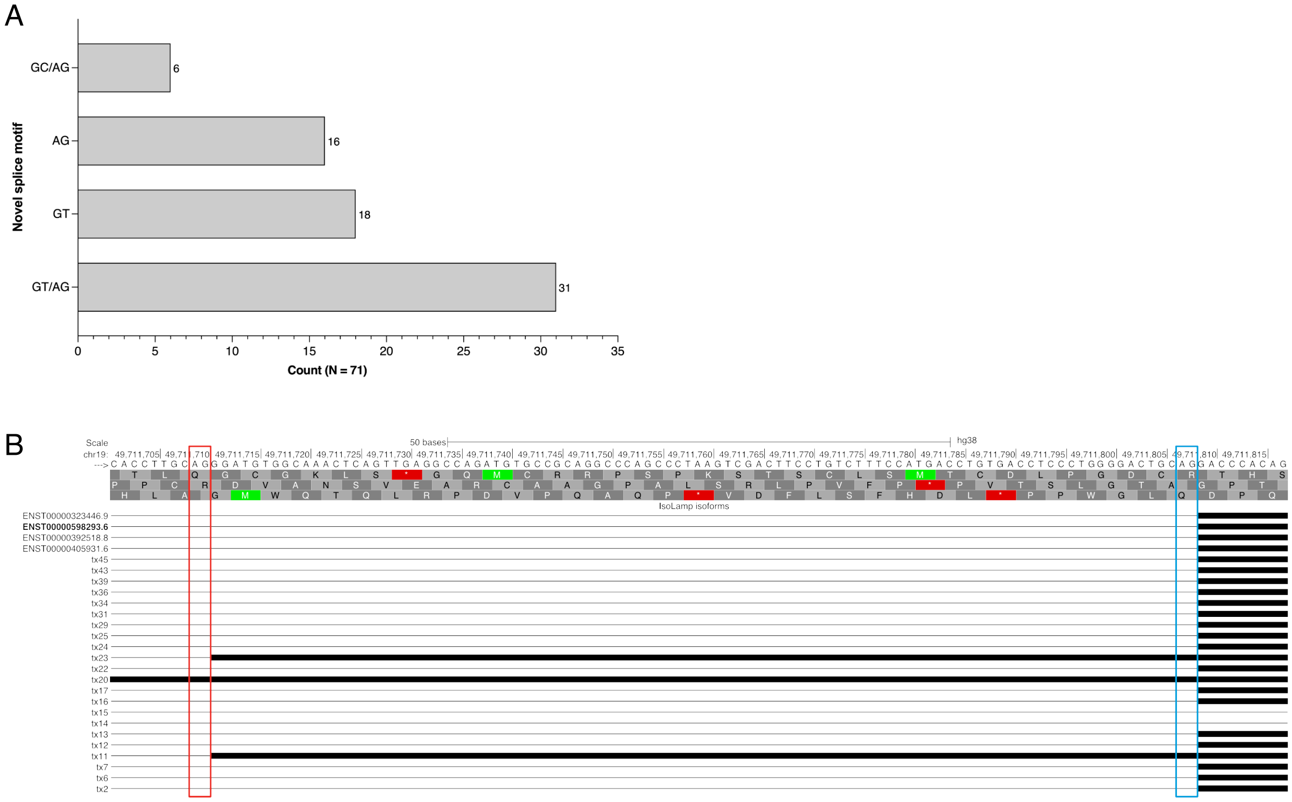

Fig. S10. Novel alternative splicing counts. A. Count of novel isoform splice pairing. Isoforms classified as containing at least one novel splice site (ALO) were examined and the novel pair or donor/acceptor was counted. Duplicates were counted only once. **B.** UCSC screenshot of an example novel splice acceptor (red box, GT/**AG**, +98 nt) detected in two novel isoforms (Tx 11 and 23) in canonical exon 17 (blue box) for the schizophrenia risk gene *CPT1C*.

Figure S11.

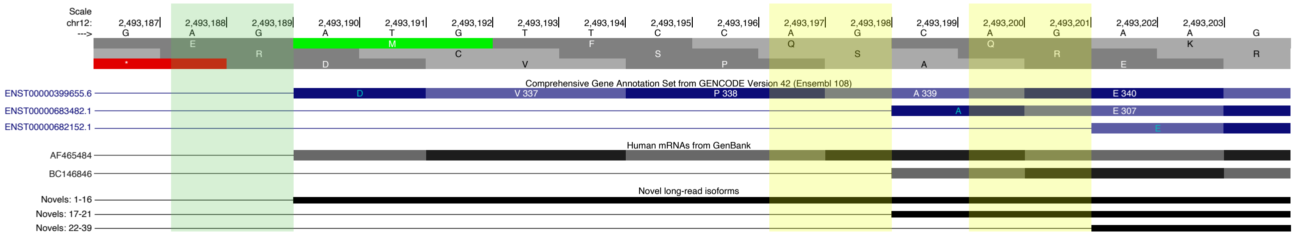

Figure S11. UCSC screenshot of *CACNA1C* splicing hotspot. Long-read sequencing identified 10 novel isoforms (black tracks) that support one of two annotated alternative splicing events (yellow boxes) within a 12 nt region (chr12:2,493,190-2,493,201) of exon 7 in *CACNA1C*. 11 novel isoforms also supported the use of the canonical (ENST00000399655.6) acceptor site (green box).

Figure S12.

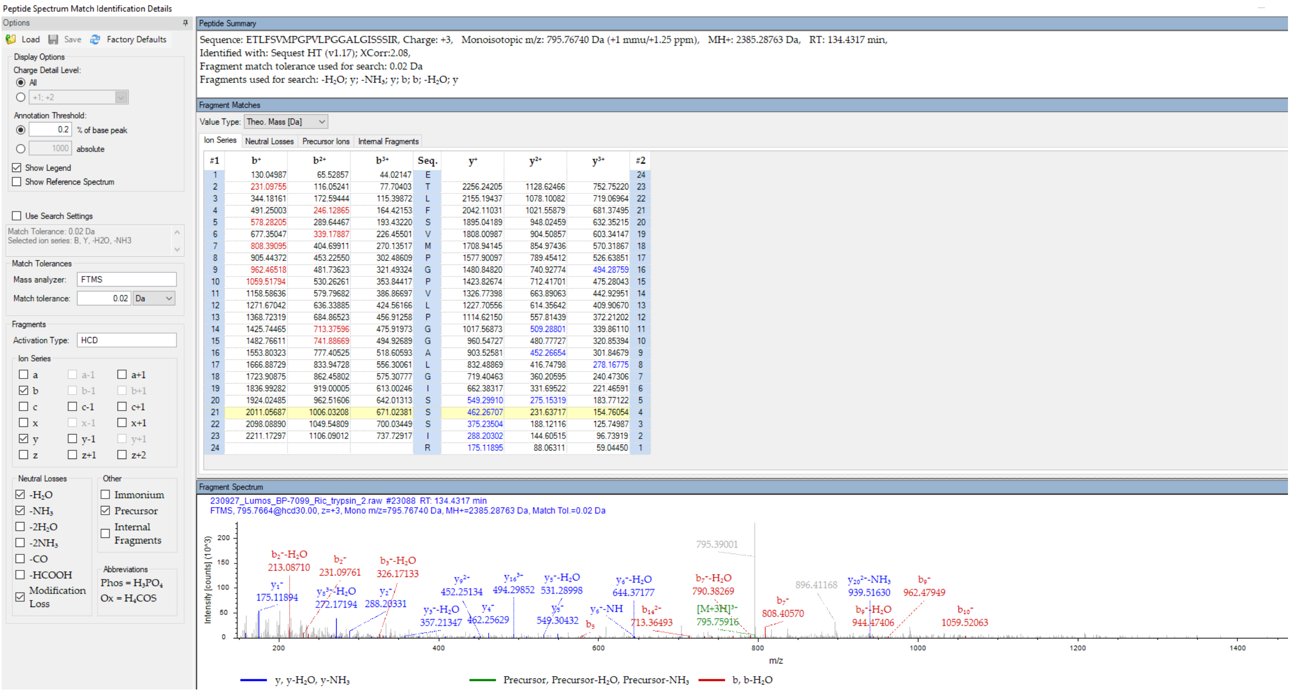

Fig. S12. Screenshot peptide spectrum. Annotated MS/MS spectrum highlighting matched b- and y-type ions providing proteomic [ETLFSVMPG//PVLPGGALGISSSIR] evidence for novel skipping of canonical exon 22 in *ITIH4* detected using long-reads.

Figure S13.

A

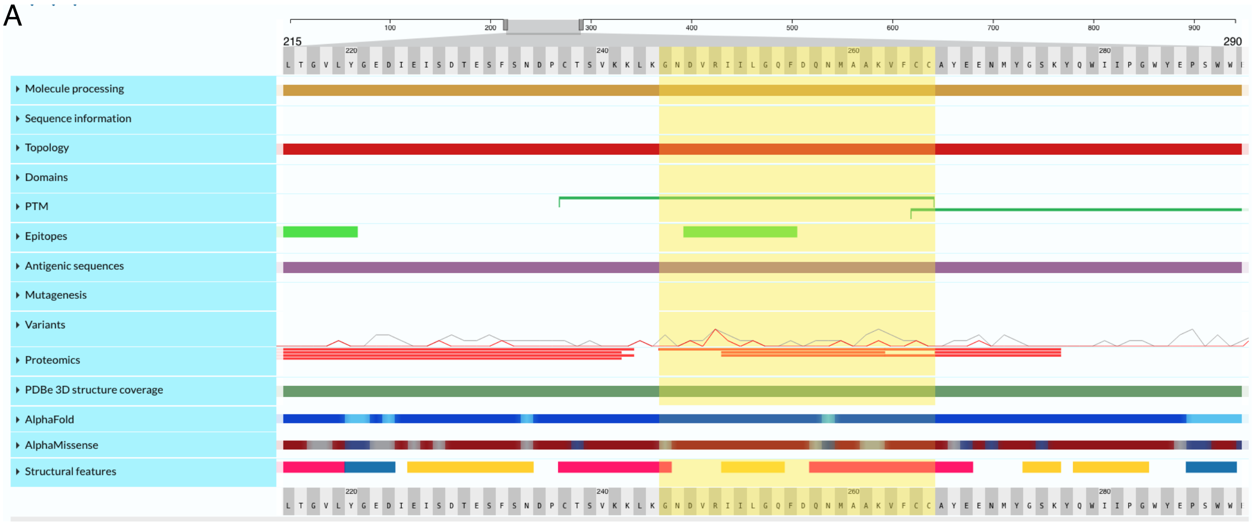

B

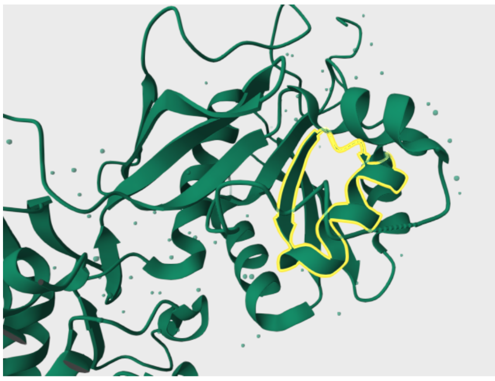

C

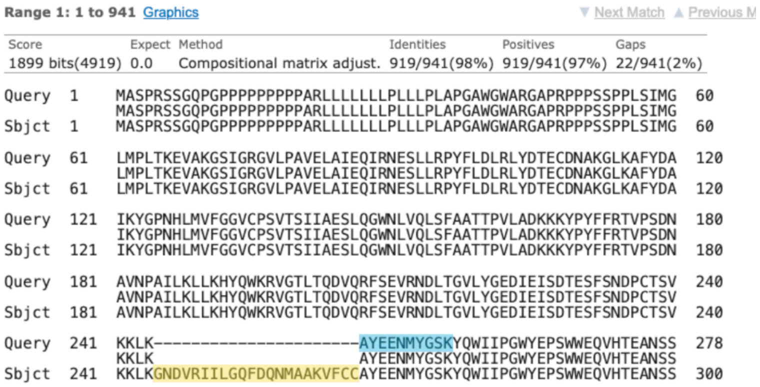

Fig. S13. Confirmation of GABBR2 exon 5 skipping. A. UniProt feature viewer screenshot of positions 215 – 290 (75 aa) of GABBR2 with skipped peptides highlighted in yellow (22 aa). Removal of these amino acids show disruption of an extracellular domain (topology), disulphide bond (PTM), epitope and structural features including beta strand (yellow box) and helix (pink box) as also shown in **B.** an x-Ray model of GABBR2 (ID: 4F11, 2.38 Å, 42 – 466). **C.** Protein Blast (NCBI) screenshot of the translated ORFs (1 – 300) for Tx29 (Query) and *GABBR2* canonical (Sbjct) isoforms indicates the skipped exon 5 (yellow box) and confirmed peptide (blue box).

Figure S14.

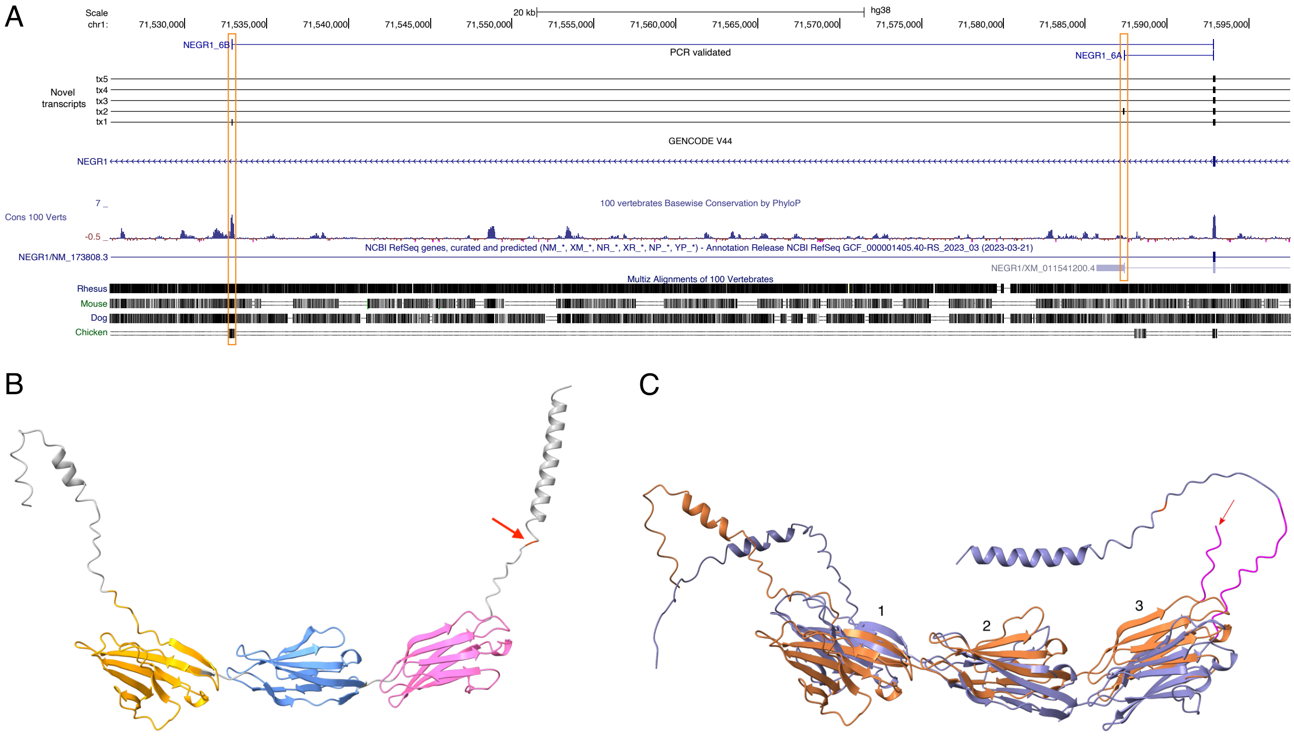

Fig. S14. *NEGR1* splice isoforms and protein prediction. A. *NEGR1* novel exons were validated using Sanger sequencing of PCR amplicons and sequence reads were aligned and viewed using UCSC Genome Browser. Orange boxes indicate the novel exons and highlight high vertebrate conservation for exon 6b and predicted (NCBI RefSeq) termination site for exon 6a. **B.** AlphaFold protein prediction of the canonical *NEGR1* isoform (ENST00000357731). Three Ig-like domains are coloured according to the reported amino-acid positions (UniProt: Q7Z3B1); 1 (orange), 2 (blue) and 3 (pink). A GPI anchor residue (red) is shown at position 324 aa (C-terminal, red arrow). **C.** Overlaid AlphaFold protein predictions of novel Tx1 (purple) and Tx2 (orange). Ig-like domains are numbered 1-3, the GPI anchor (red) is present in Tx1 and the termination of Tx is indicated by a red arrow. Novel residues (pink) are indicated at the C-terminal end for both transcripts, Tx1: 14 aa and Tx2: 7 aa.

Figure S15.

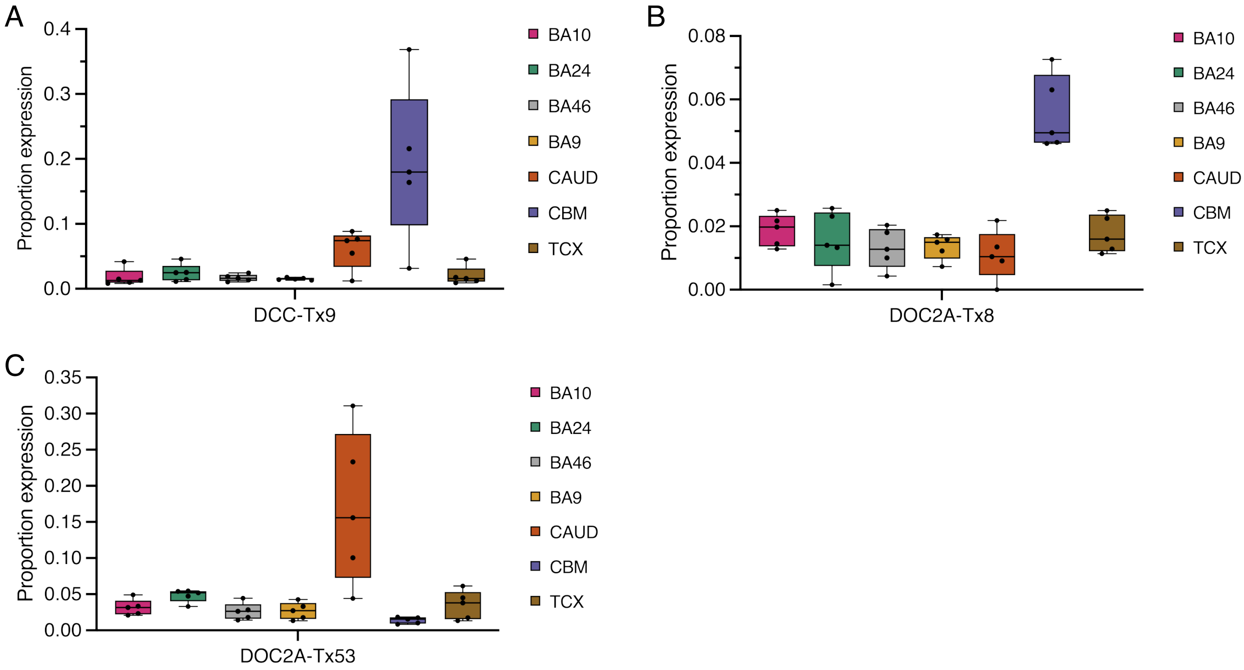

Fig. S15. A. Brain region enriched expression of novel isoforms. Expression of each novel isoform is shown as a proportion of the total gene expression for each brain region. **A.** *DCC* novel transcript 9 (Tx9) uses a known (ENST00000581580) alternative 3’SS (-60 nt) in cassette exon 17 and had significantly higher TPM in CBM. **B-C.** *DOC2A* novel transcripts. Tx8 (B) used a novel splice donor in canonical 5’UTR exon 1 (GT, +158 nt). One caudate sample recorded no expression and has been truncated from the plot. (C) extended the known reference isoform ENST00000574405 to the canonical translational stop and had significantly higher TPM in caudate. Brodmann’s Area (BA), caudate (CAUD), cerebellum (CBM) and temporal cortex (TCX). Ordinary one-way ANOVA Tukey’s multiple comparison adjusted P value: **= P ≤ 0.01, ***=P ≤ 0.001, ****=P ≤ 0.0001.

Figure S16.

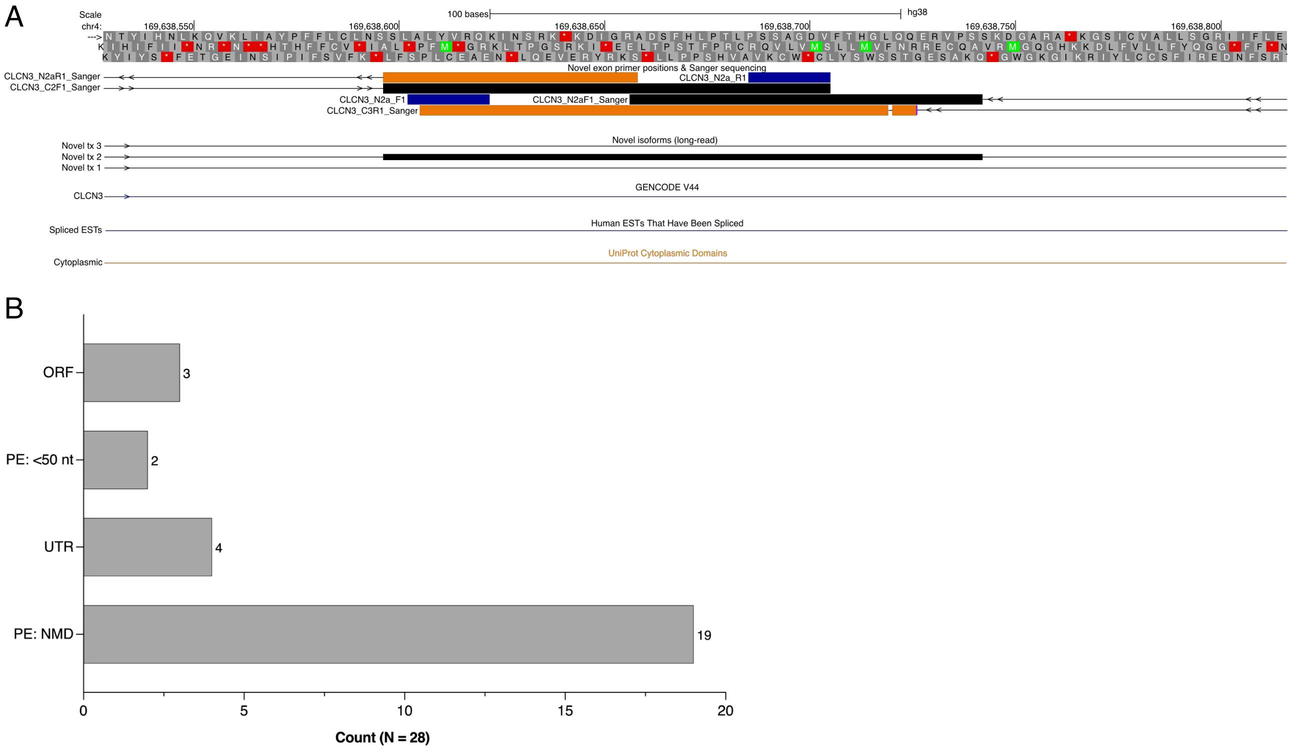

Fig. S16. Novel exon validation in *CLCN3*. A. Novel exon 2a in the schizophrenia risk gene *CLCN3*, identified in long-read sequencing data, was validated by PCR using primers designed in the flanking cassette exons 1 and 3 (ENST00000513761). The novel exon sequence shown in novel transcript (Tx) 2 was validated with Sanger sequencing from the 5’ canonical exon 2 (C2F1) to the reverse primer within the novel sequence (N2aR1) and from the novel sequence (N2aF1) to the 3’ canonical exon 3 (C3R1). Direction of reads are indicated with arrows. Black and orange boxes indicate forward and reverse Sanger sequence respectively. Blue boxes indicate forward and reverse primers within the novel exon. A known cytoplasmic domain is shown by an orange track. Key: expressed sequence tag (EST). **B. Novel exon categories.** The impact of novel exon inclusion on the open reading frame of novel isoforms was predicted using Expasy [45] and then classed into groups, predicted to retain the open reading frame (ORF), inclusion of premature termination codon or ‘poison exon’ that was predicted to lead to nonsense-mediated decay (PE:NMD) or was <50 nt from the final exon junction (PE: <50 nt) or was with the 5’ or 3’ untranslated regions (UTR).

Figure S17.

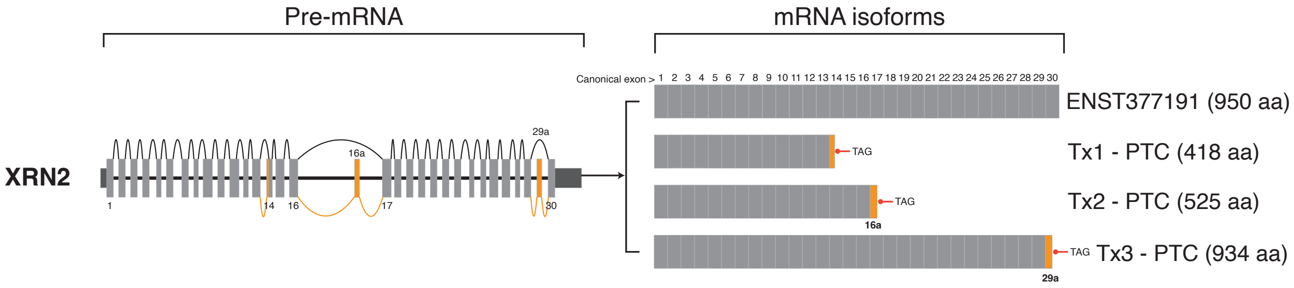

Fig. S17. Splice graph of *XRN2* novel isoforms containing novel exons. Dark and light grey boxes indicate 5' and 3' UTR and coding exons respectively. Orange lines and boxes indicate novel splicing events and exons. Novel transcript 1 (Tx1) contains a novel splice acceptor (AG) within exon 14 (+24 nt) leading to a premature termination codon (PTC) and predicted nonsense mediated decay. Novel transcript 2 (Tx2) includes the validated novel exon 16a (54 nt) which was also predicted to encode a PTC. Novel isoform 3 (Tx3) contains a validated novel exon (29a) which encodes a PTC <50 nt from the final exon junction. Tx3 was predicted to lead to a truncated protein (934 aa). ".." indicates 0's removed for brevity.

Figure S18.

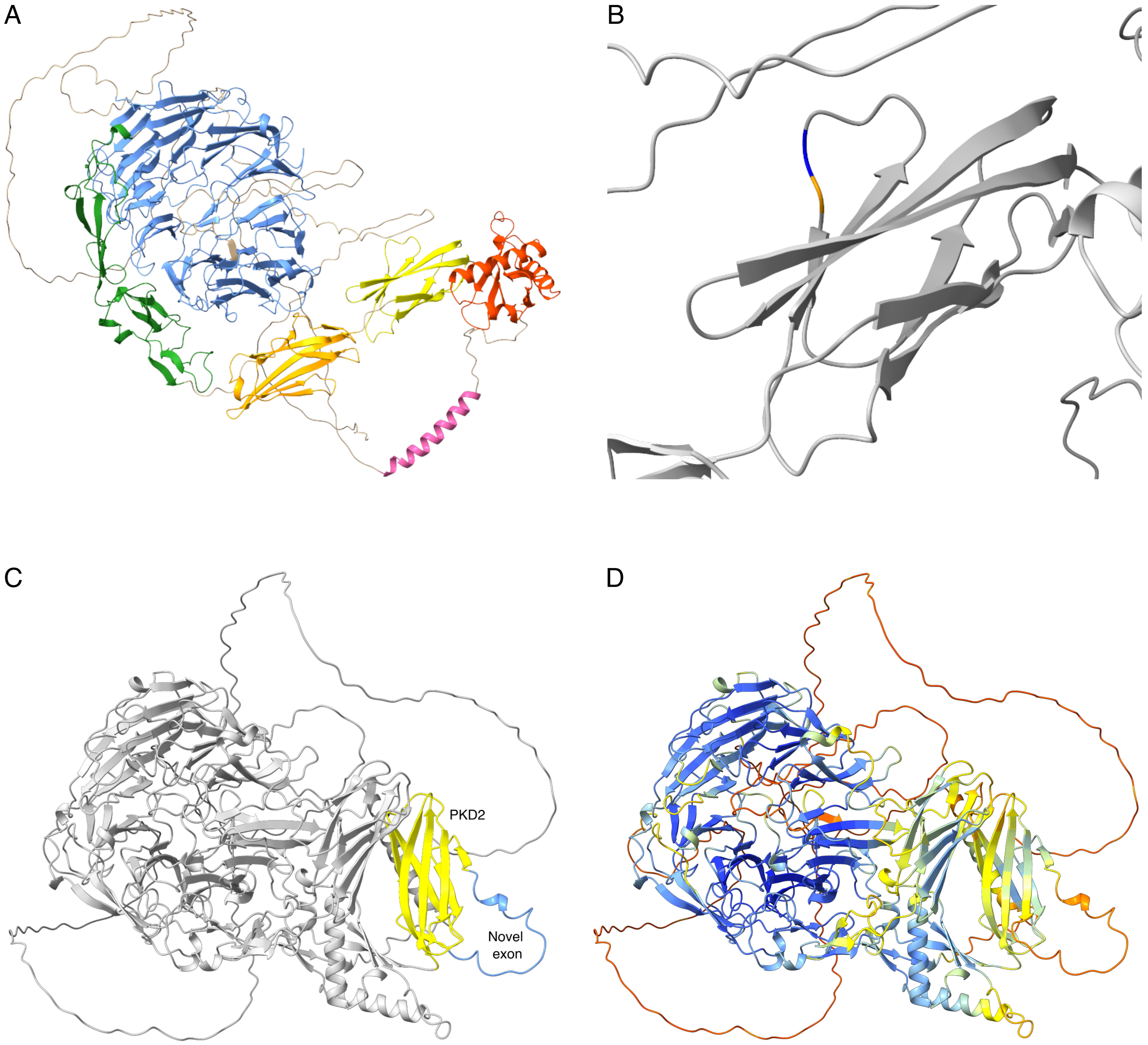

Fig. S18. *SORCS3* novel exon and protein structure predictions. A. AlphaFold prediction of *SORCS3* canonical isoform (ENST00000369701.8: Q17R88) coloured by domain: β-propeller (blue), 10CC domain domains (green), polycystic kidney disease (PKD) domains PKD1 (orange) and PKD2 (yellow), SorCS membrane proximal (SoMP) (red) and transmembrane domain (pink). **B.** Zoomed view of the PKD2 domain indicating LYS:956 (blue) and PRO:957 (orange) where frame-retaining novel exon 20a (60 nt) is inserted. **C.** Protein structure prediction of novel transcript 1 (Tx1) containing the novel exon (blue) within the PKD2 domain (yellow). **D.** AlphaFold per-residue confidence scores (pLDDT) (0-100) for novel transcript 1: very high (>90, blue), confident (90-70, light-blue), low (70>50, yellow) and very low (<50, orange).
