# Supplementary material for: Long-read sequencing reveals the RNA isoform repertoire of neuropsychiatric risk genes in human brain
Source: Genome Biol. 2025 Sep 23;26:298. doi: 10.1186/s13059-025-03724-1 (PMC12455821; doi:10.1186/s13059-025-03724-1)
Supplement: Supplementary file 4 — Additional file 4. IsoVis (v1.6 (2024–02-29)) stack and heatmap output for each risk gene sorted by descending TPM. Figures A – Z, AA – AG. [file 13059_2025_3724_MOESM4_ESM.docx]

**Additional File 4.** IsoVis (v1.6 (2024-02-29)) stack and heatmap output for each risk gene sorted by descending TPM. The protein track is shown at the top of the isoform stack and is coloured by source; pfam (purple), mobidblt (green), coils (blue), phobius (brown). Protein domain descriptions are given for each gene where novel isoforms were identified. The second track from the top indicates the canonical transcript (Ensembl). The open reading frame (black boxes) is shown for known transcripts. TPM for each isoform are shown ranging from relatively low (blue) to relatively high (orange), TPM range and middle value is specific to each isoform.

**A.** *AREL1*. Protein track shows from left to right: HECT domain, consensus disorder region and Filamin/ABP280 repeat.
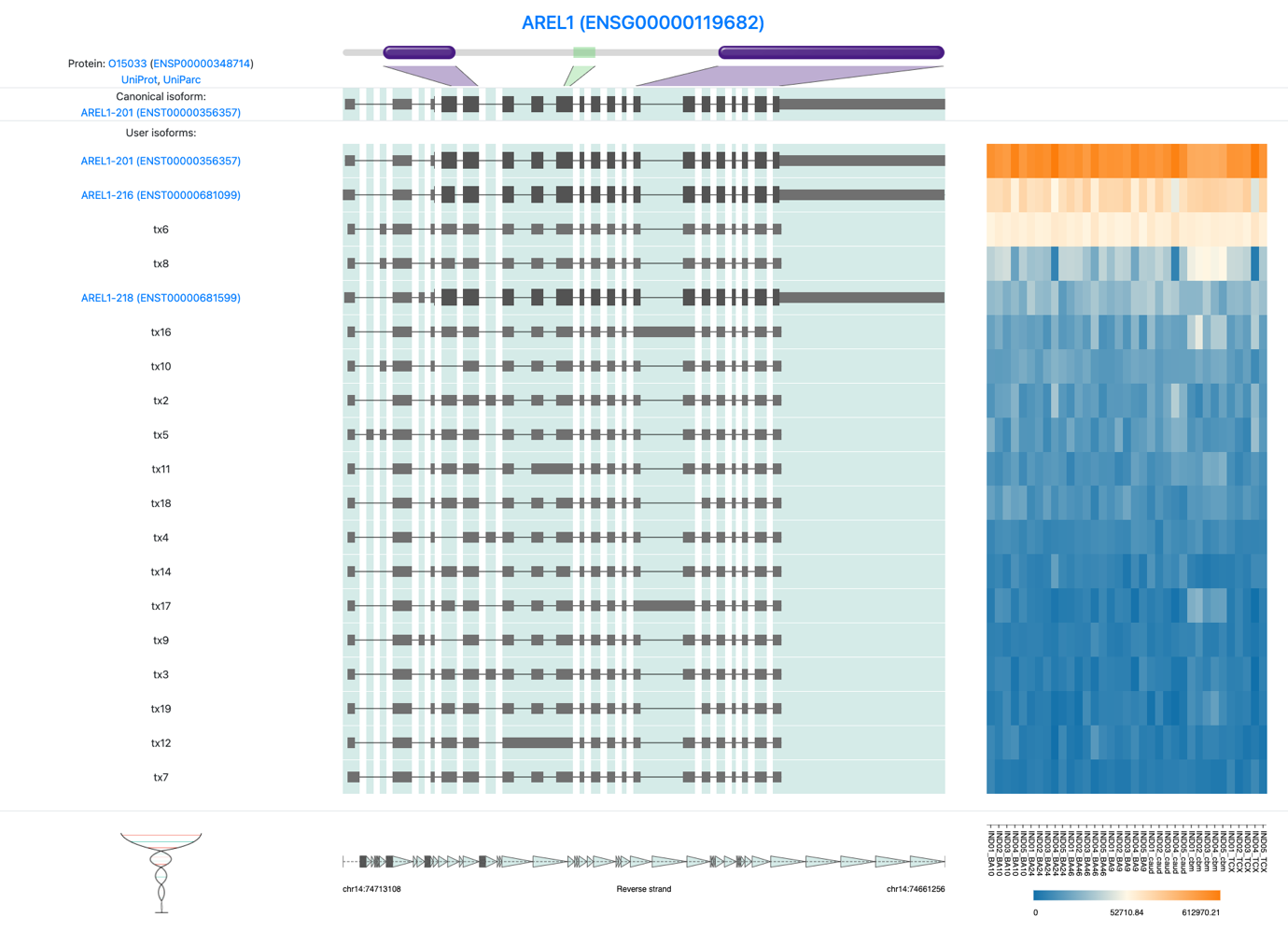


**B.** *ATG13*. Protein track shows from left to right: Autophagy-related protein 13 and three consensus disorder predicted regions.

**
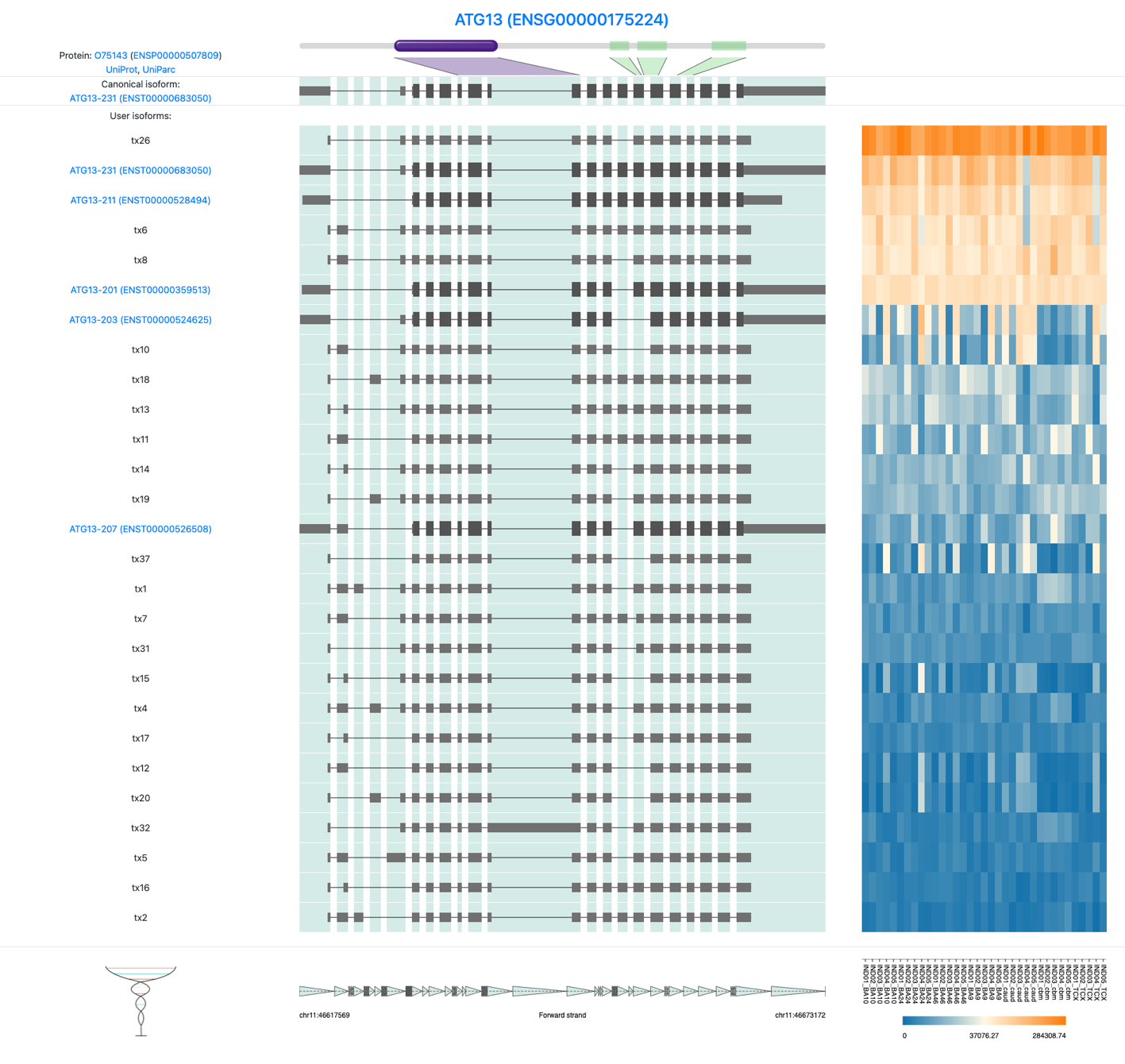
**

**C.** *CACNA1C*. Protein track indicates ion transport proteins (purple), consensus disorder predicted regions (green) and coils (blue).

**
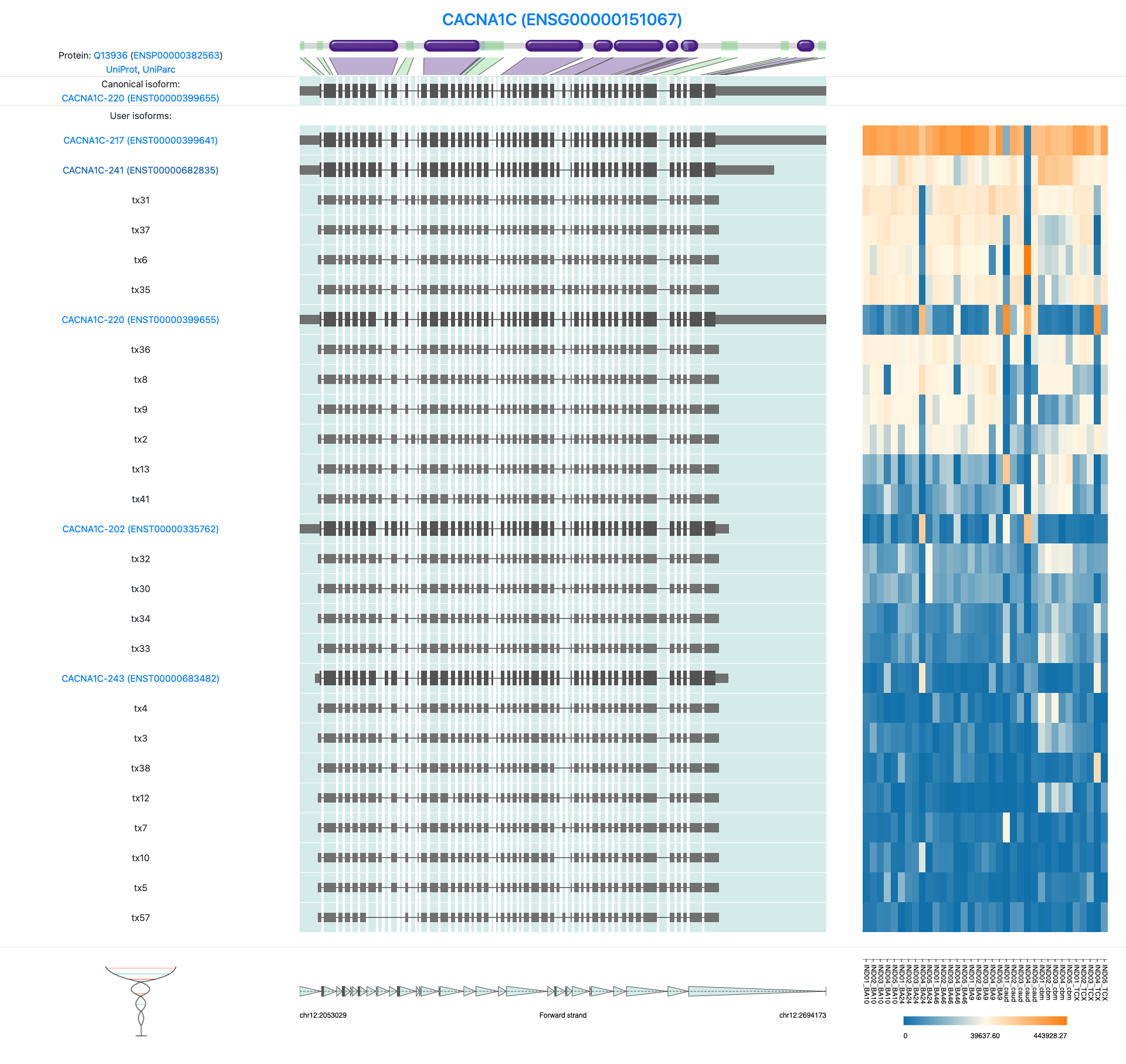
**

**D.** *CHADL*. Protein track indicates a signal peptide (brown) and two consensus disorder predicted regions (green).


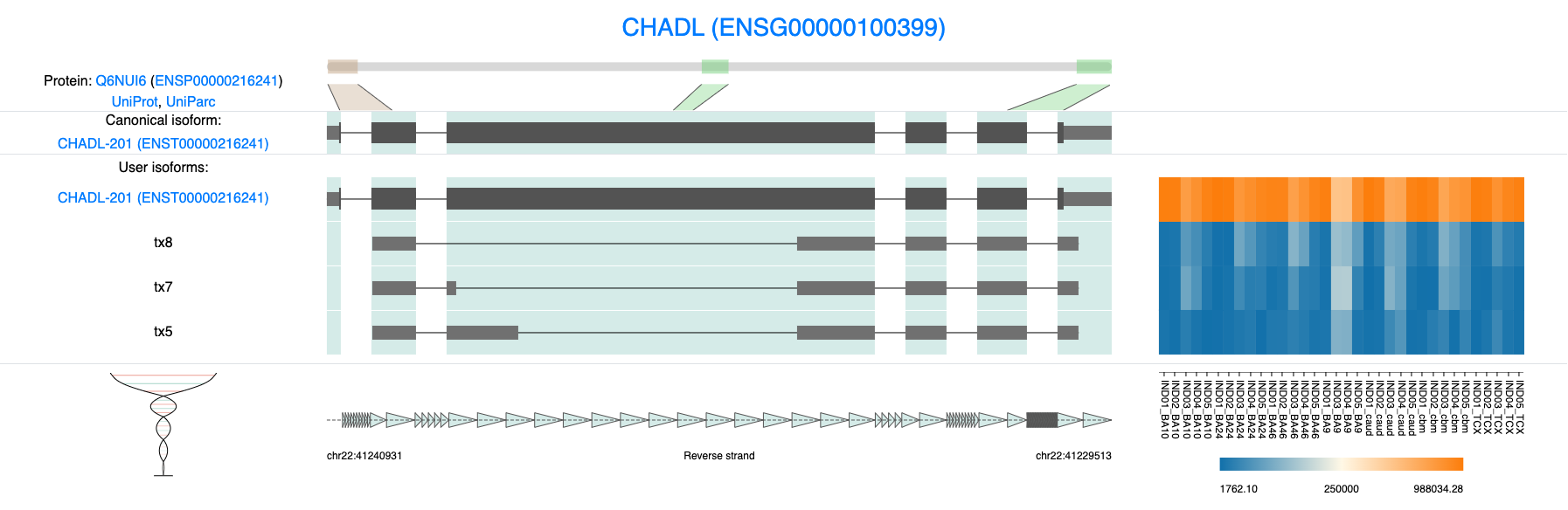


**E.** *CLCN3*. Protein track indicates from left to right: voltage gated chloride channel and CBS domain.


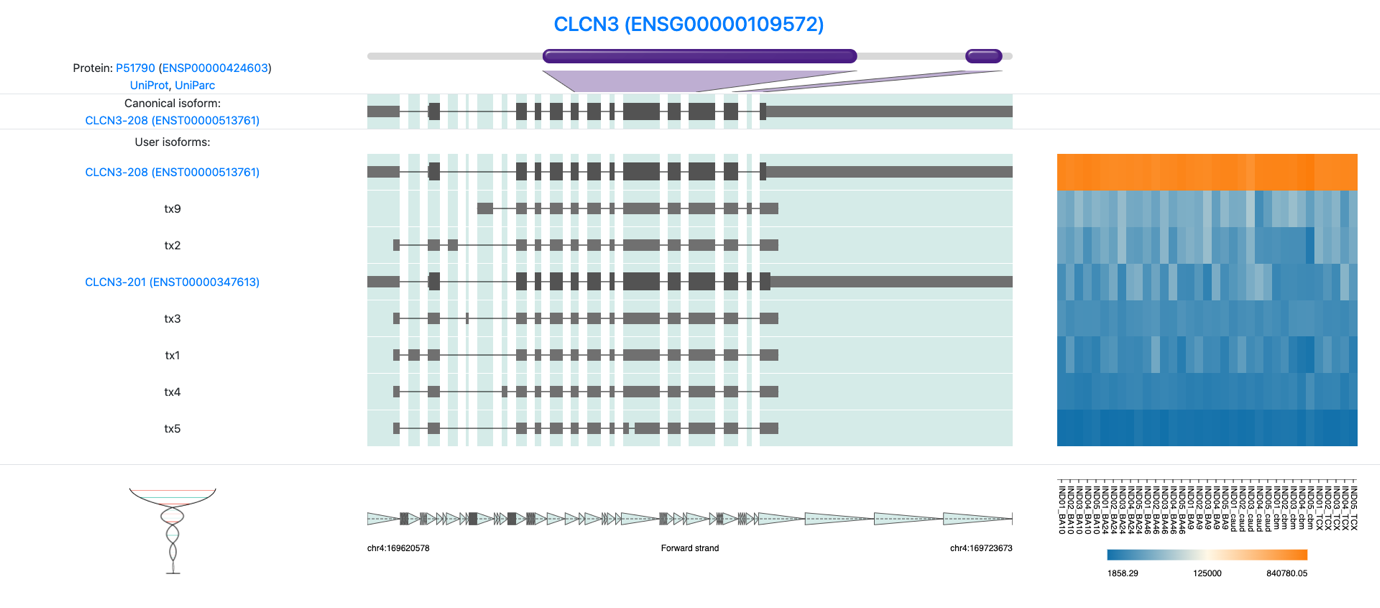


**F.** *CLU*. Protein track indicates a signal peptide (brown), clusterin domain (purple) and two coils (blue).


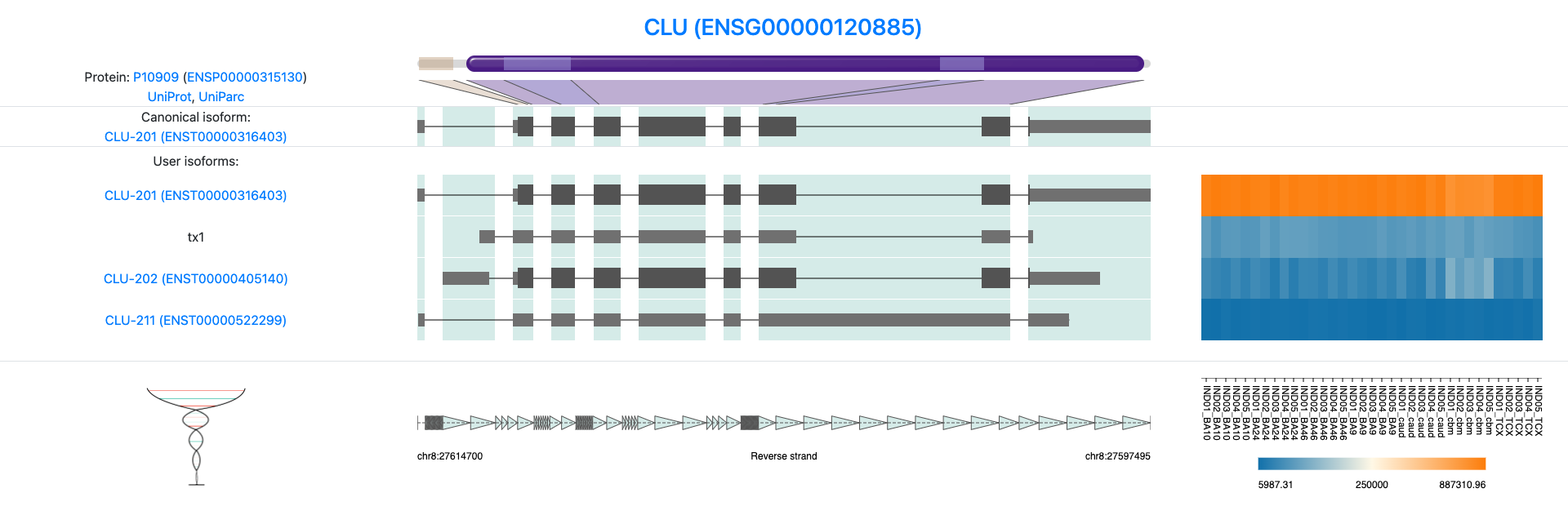


**G.** *CNTN4*. Protein track indicates a signal peptide (brown), six sequential immunoglobulin I-set domains followed by two fibronectin type III domains (purple) and two consensus disorder predicted regions (green).


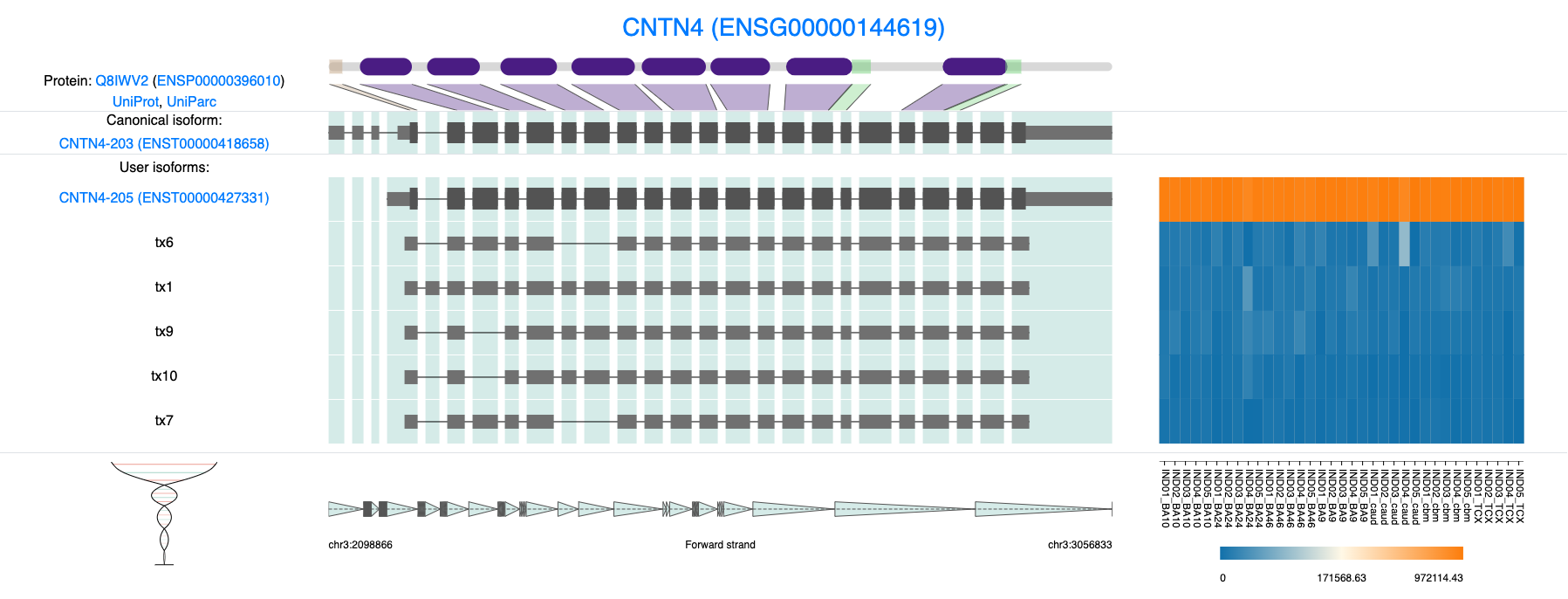


**H.** *CPT1C*. Protein track indicates 5’ > 3’ a carnitine O-palmitoyltransferase N-terminus followed by a Choline/carnitine o-acyltransferase domain (purple) and a consensus disorder predicted region (green).


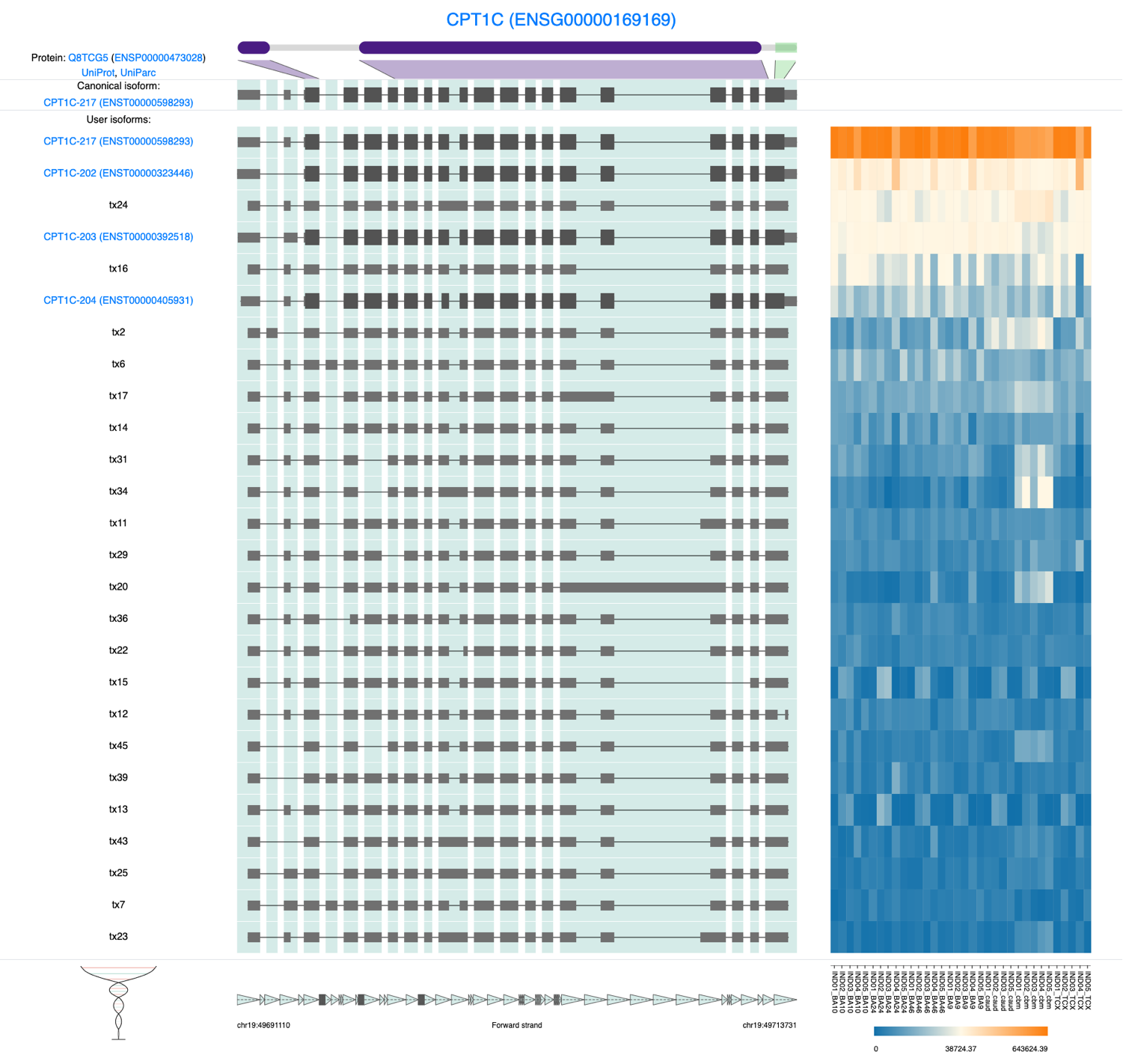


**I*.*** *CSMD1*. Protein track indicates a signal peptide (brown) and alternating CUB and Sushi (SCR) repeats (purple).


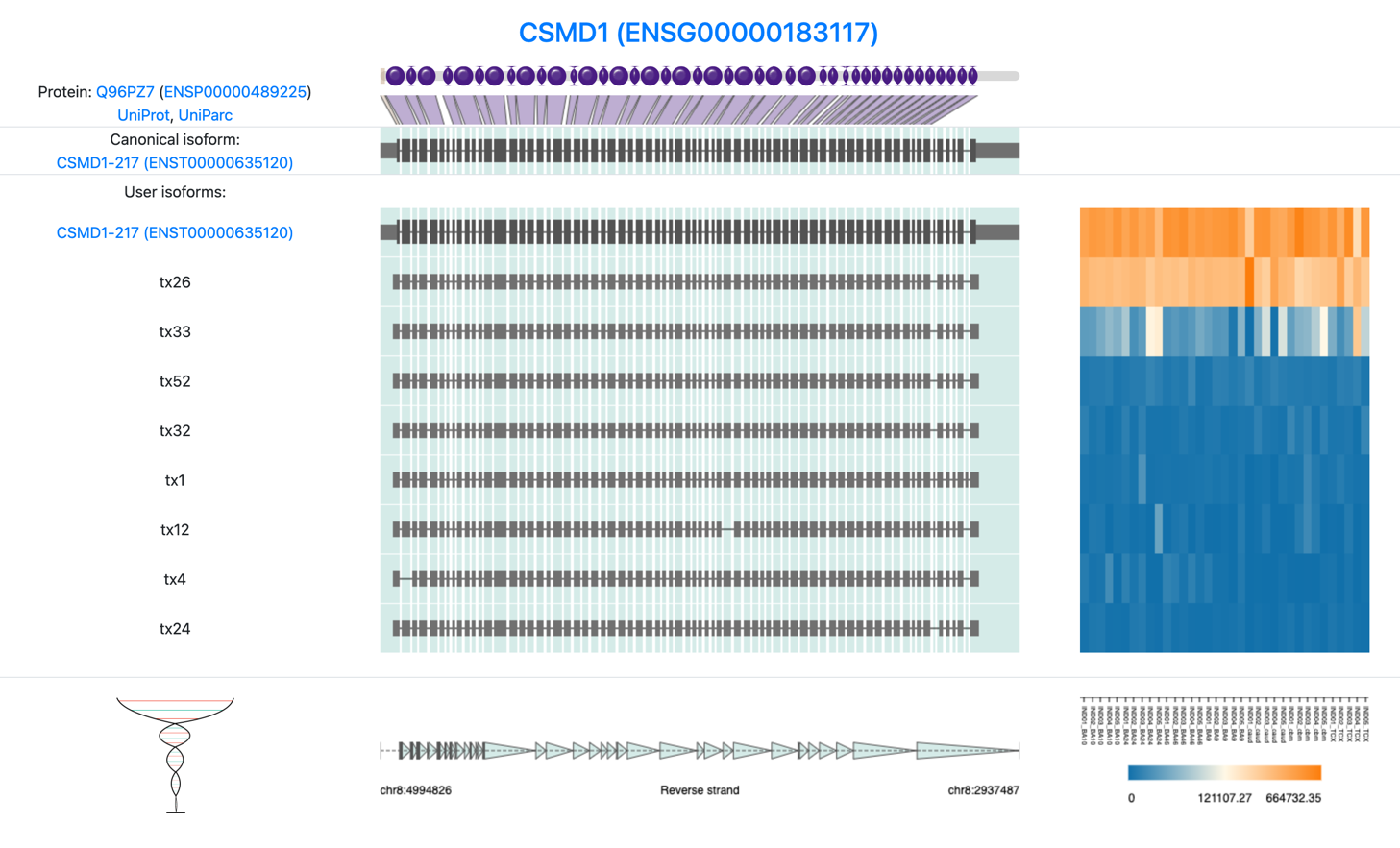


**J.** *DCC*. Protein track indicates 5’ > 3’ signal peptide (brown) followed by three Immunoglobulin I-set domains, six fibronectin type III domains and a neogenin C-terminus (purple). Consensus disorder predicted regions and coils are in green and blue respectively.

**
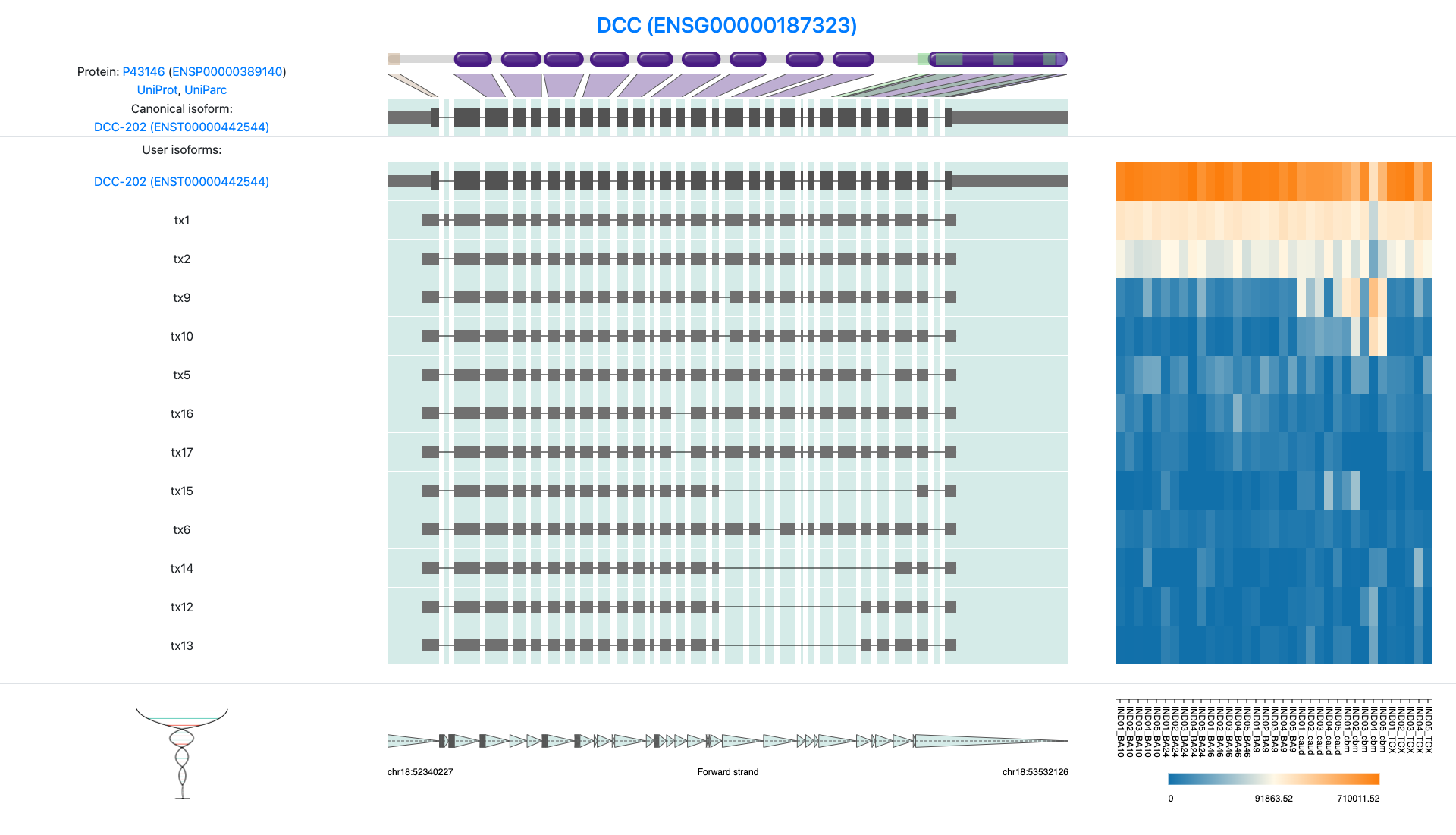
**

**K.** *DOC2A*. Protein track indicates two C2 domains (purple).


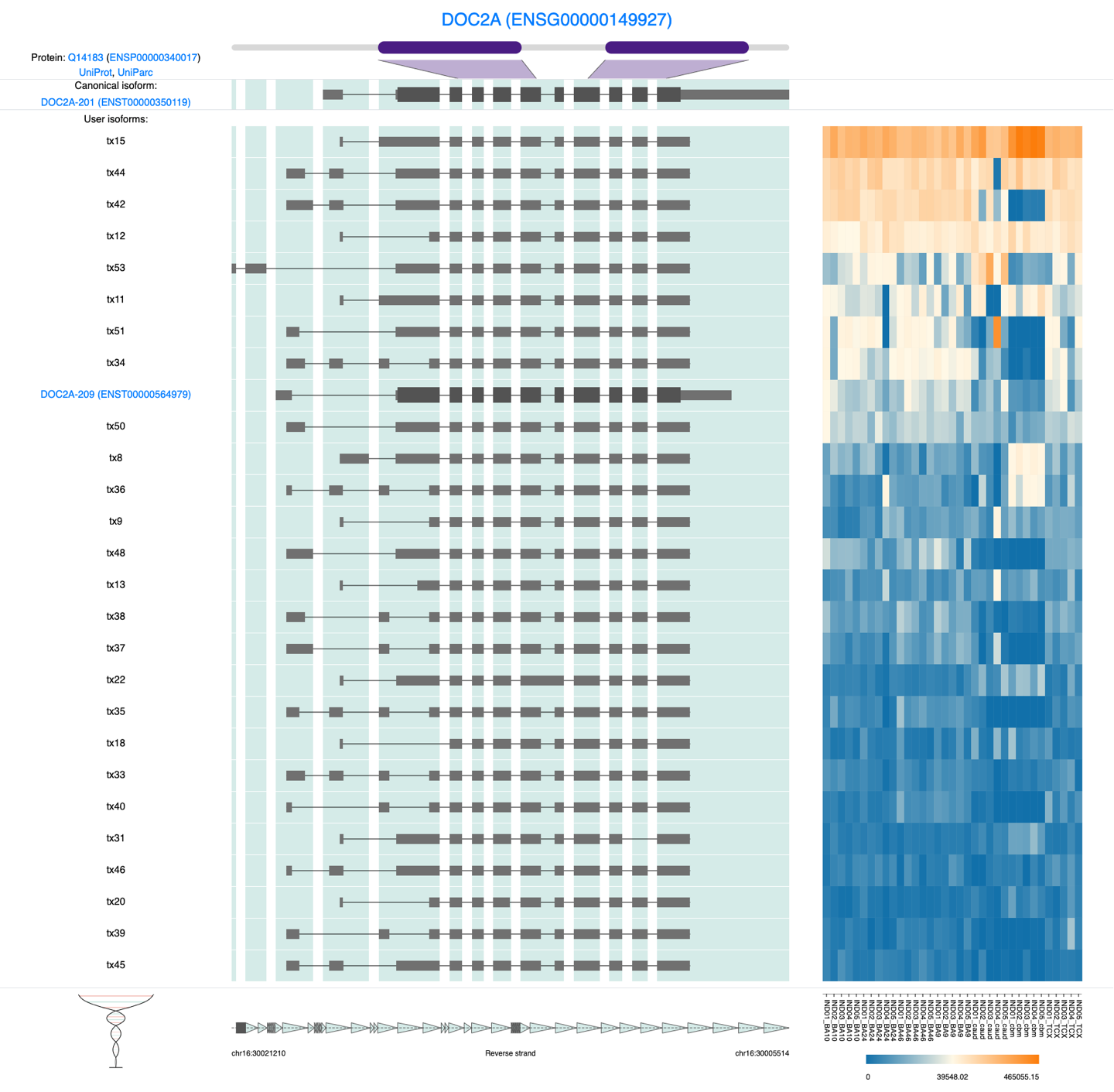


**L.** *ENKD1*. Protein track indicates consensus disorder predicted regions (green) and a Calmodulin-binding domain (purple).

**
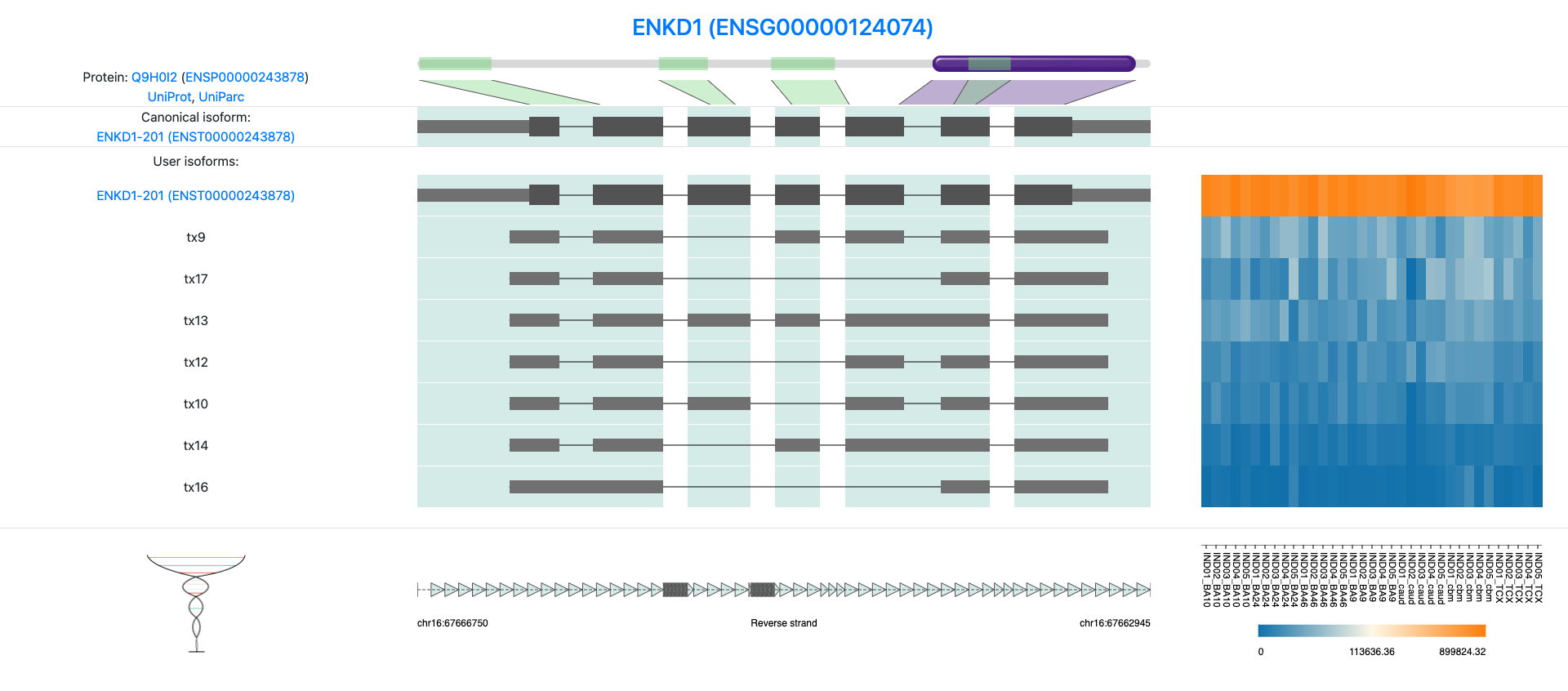
**

**M.** *GABBR2*. Skipping of canonical exon 5 (tx29, red box) was confirmed by MS. Protein track indicates 5’ > 3’ a signal peptide (brown), a receptor family ligand binding region followed by a 7 transmembrane sweet-taste receptor of 3 GCPR (purple), consensus disorder predicted regions (green) and coil (blue).


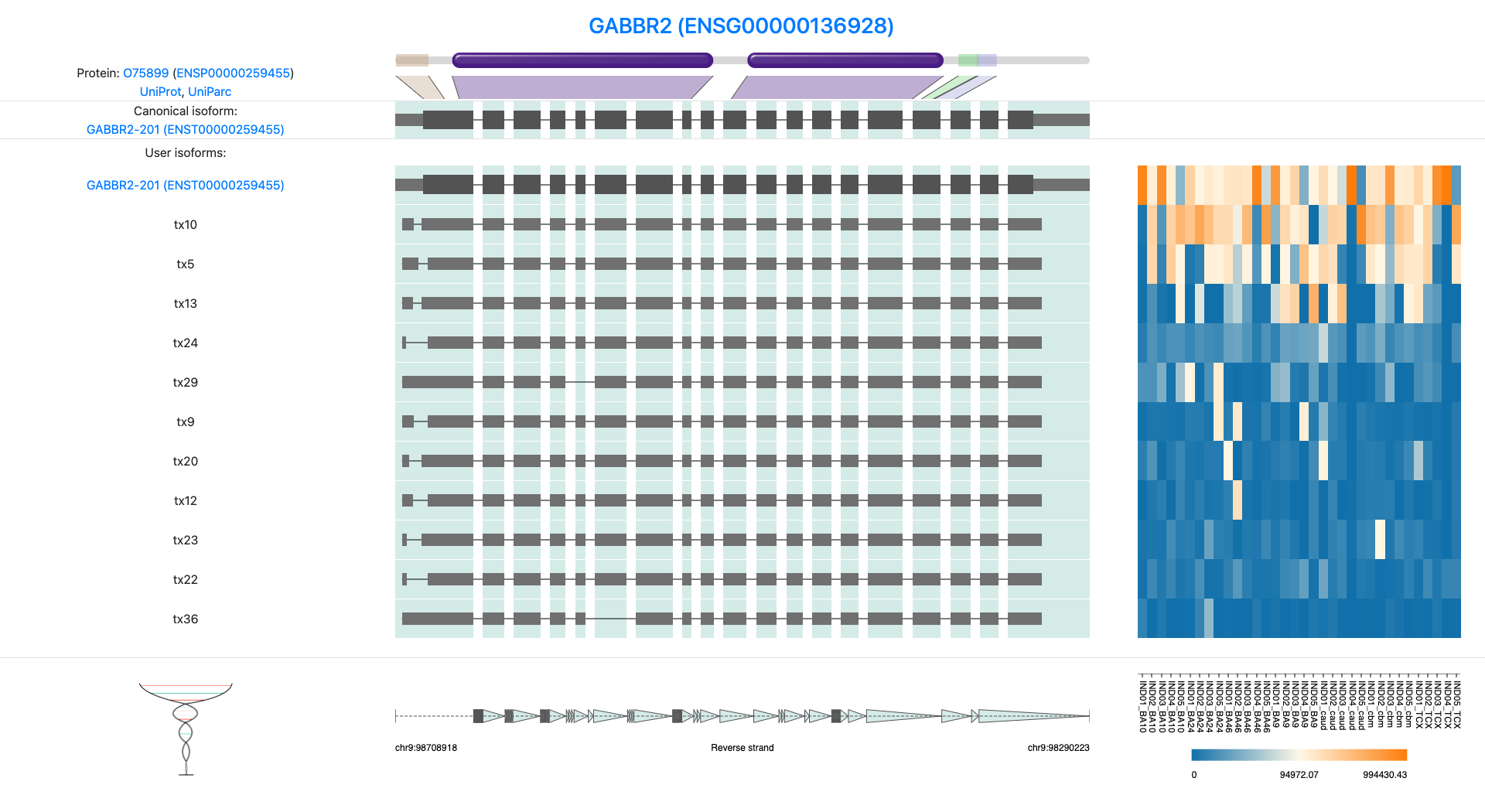


**N.** *GATAD2A*. Protein track indicates consensus disorder predicted regions (green), coil (blue) and a GATA zinc finger (purple).


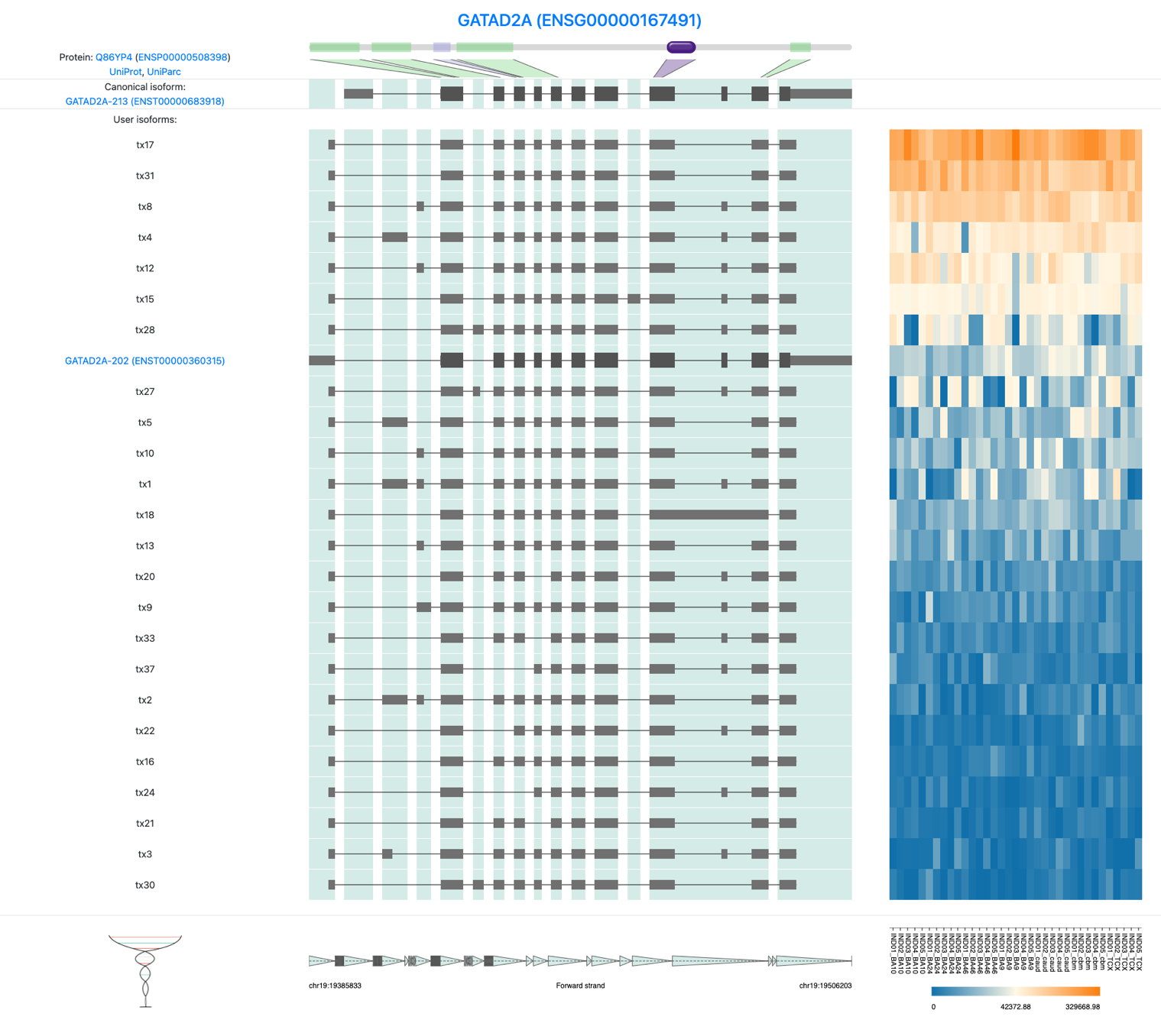


**O.** *GRIA1*. Protein track indicates 5’ > 3’ a signal peptide (brown), receptor family ligand binding region, ligated ion channel L-glutamate and glycine binding site, ligand-gated ion channel, bacterial extracellular solute-binding proteins (family 3) and a consensus disorder predicted regions (green).
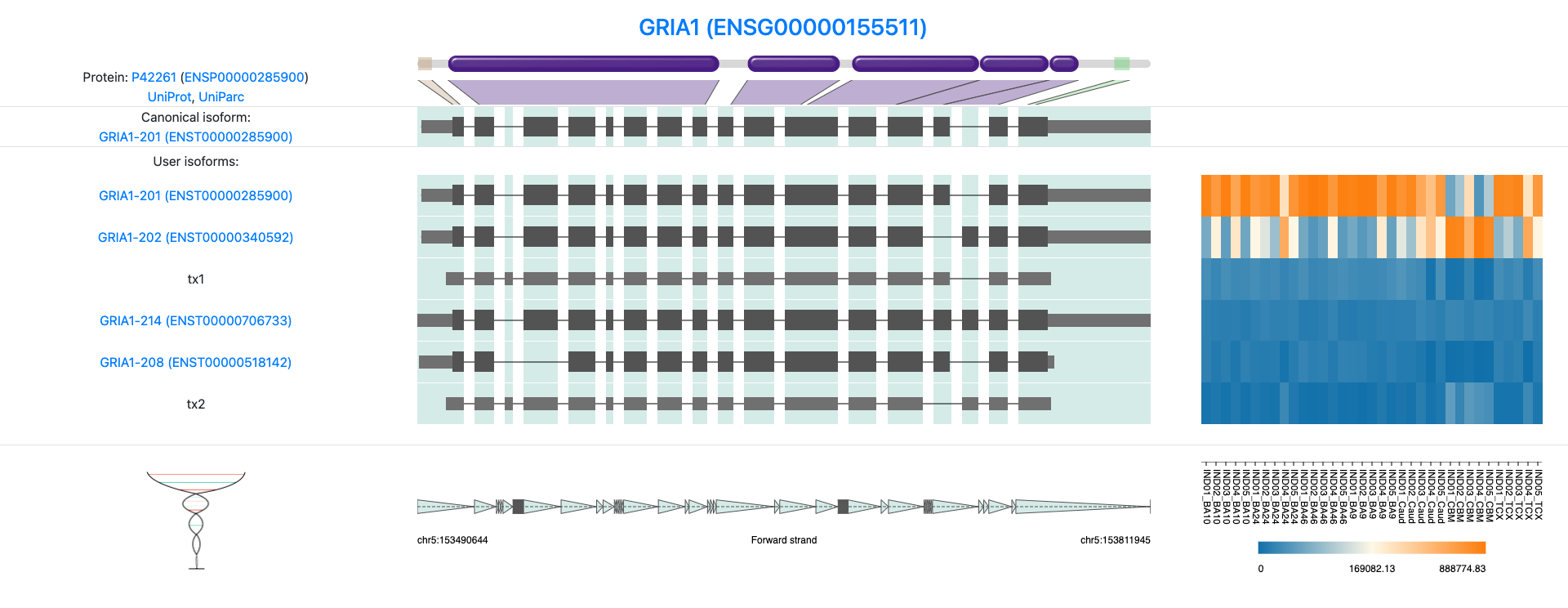


**P.** *GRIN2A*. Protein track indicates 5’ > 3’ a signal peptide (brown), receptor family ligand binding region, ligated ion channel L-glutamate and glycine binding site, ligand-gated ion channel, bacterial extracellular solute-binding proteins (family 3), ligand-gated ion channel, N-methyl D-aspartate receptor 2B3 C terminus and two consensus disorder predicted regions (green).

**
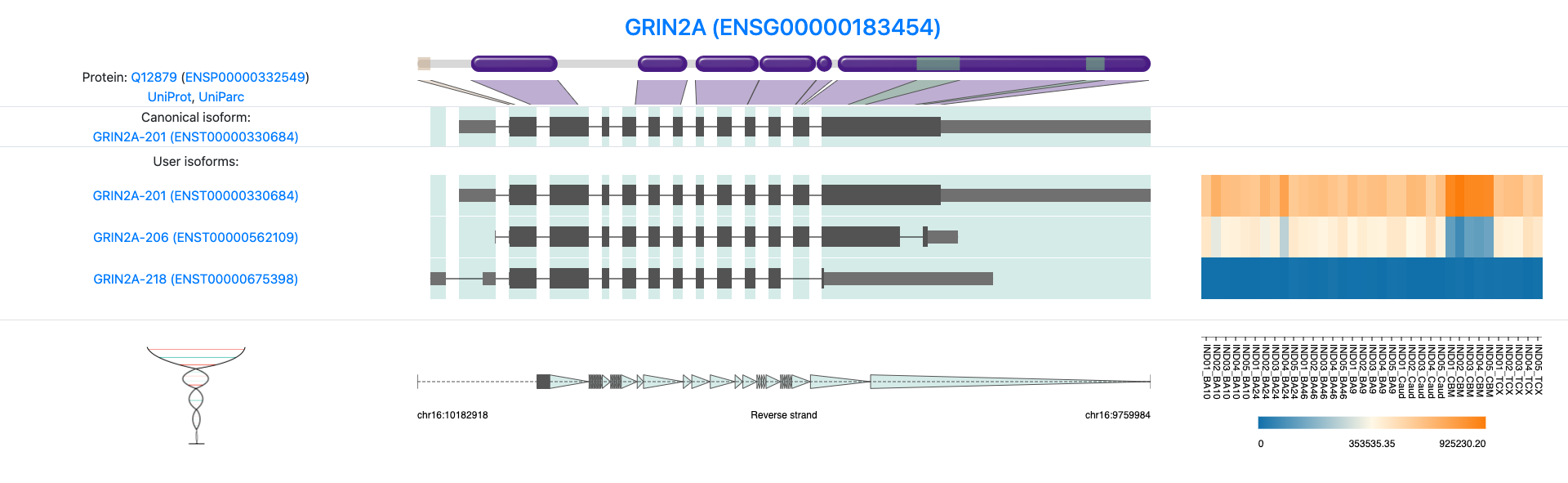
**

**Q.** *GRM3*. Protein track indicates 5’ > 3’ a signal peptide (brown), receptor family ligand binding region, nine cysteines domain of family 3 GPCR and a 7 transmembrane sweet-taste receptor of 3 GCPR (purple).

**
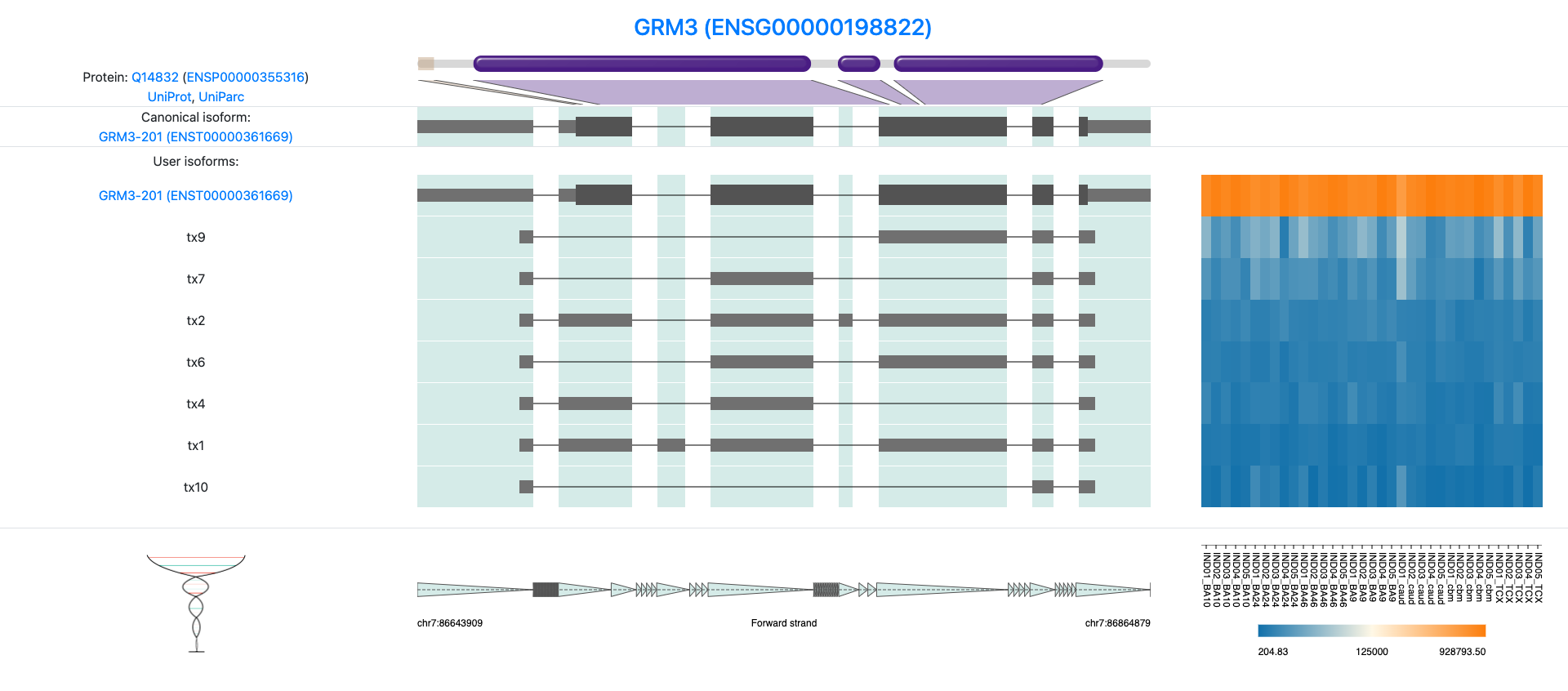
R.** *ITIH4* (top 20 RNA isoforms by TPM). Protein track indicates 5’ > 3’ a signal peptide (brown), a vault protein inter-alpha-trypsin domain (purple), von Willebrand factor type A domain (purple), consensus disorder predicted region (green) and inter-alpha-trypsin inhibitor heavy chain C-terminus (purple).


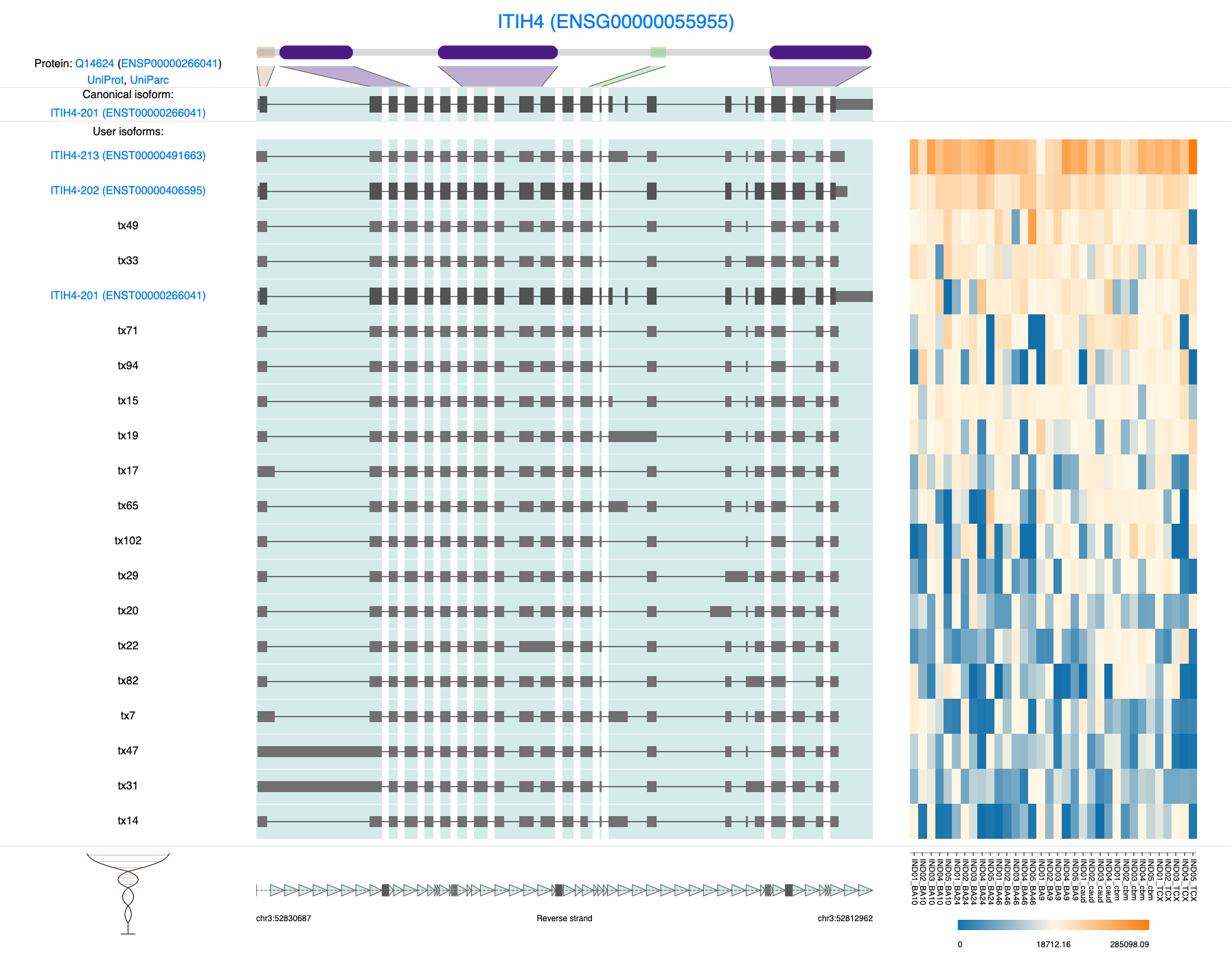


**S.** *KLC1*. Protein track indicates coil (blue) and consensus disorder predicted regions (green).


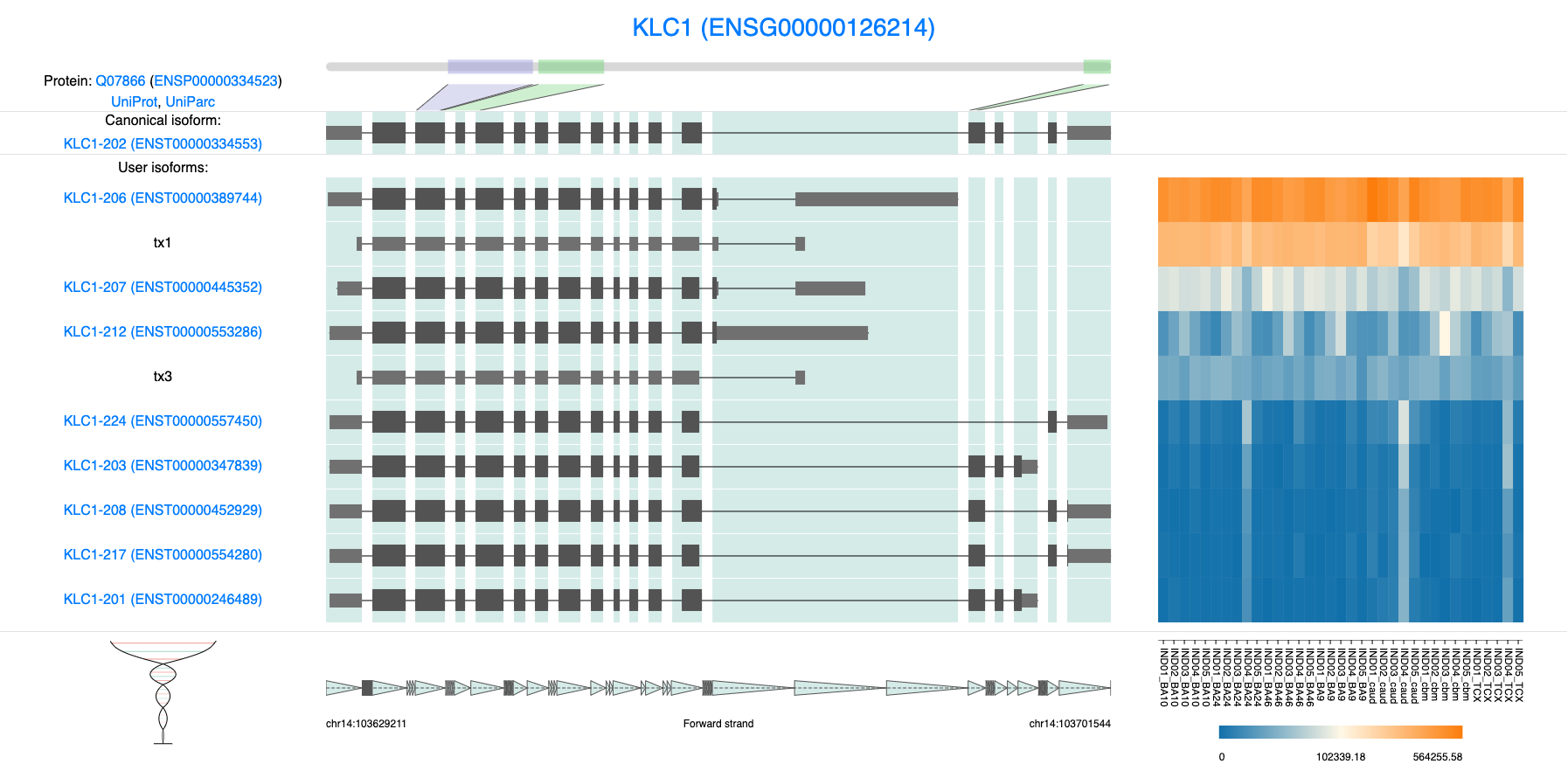


**T.** *LMAN2L*. Protein track indicates 5’ > 3’ a signal peptide (brown) and legume-like lectin family domain.

**
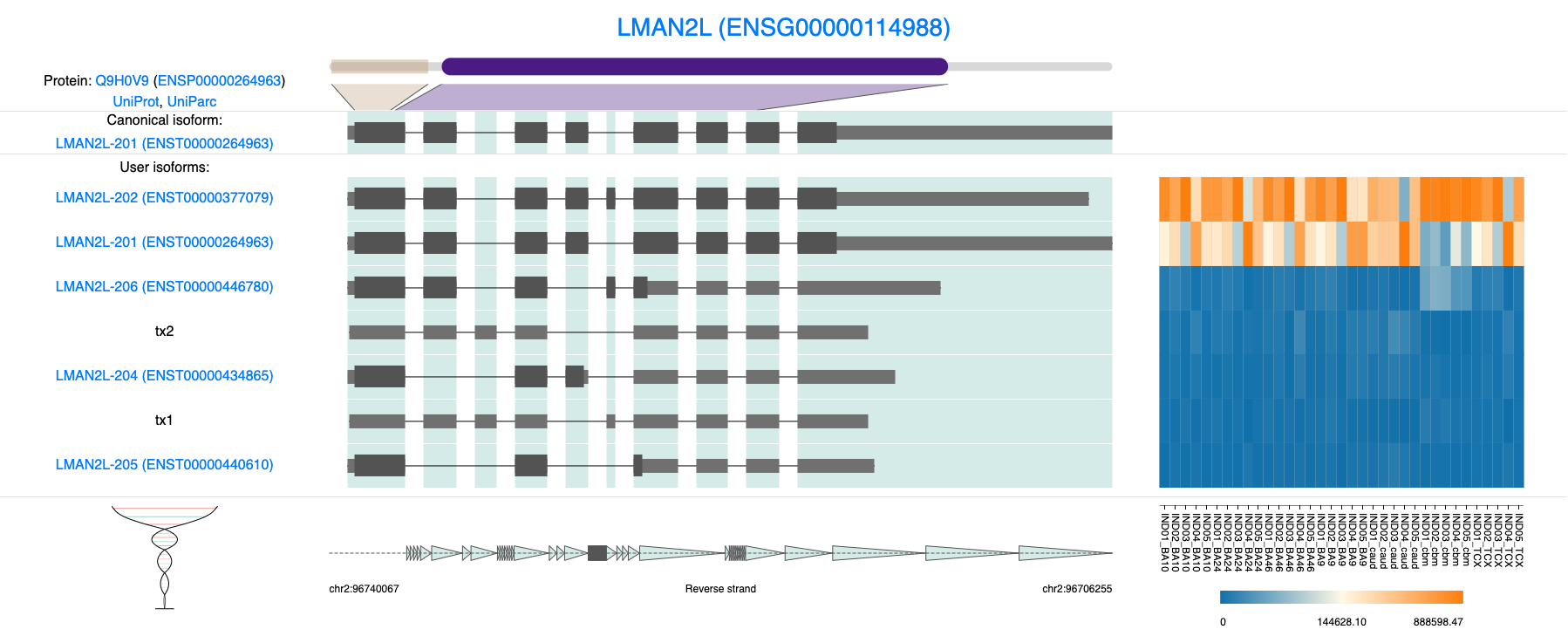
**

**U.** *MAPT*. Protein track indicates consensus disorder predicted regions (green) and four Tau and MAP protein, tubulin-binding repeats (purple).


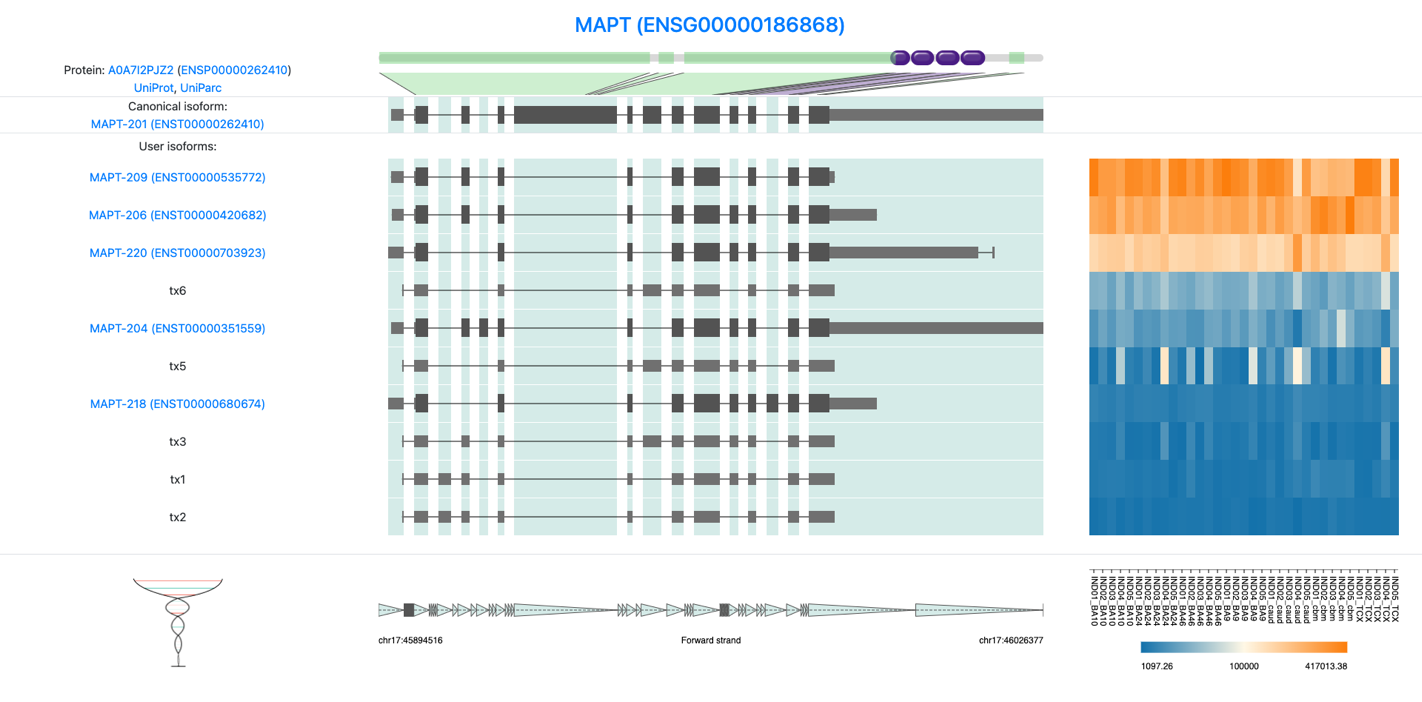


**V.** *NEGR1*. Protein track indicates 5’ > 3’ a signal peptide (brown) and three immunoglobulin-like domains (purple).


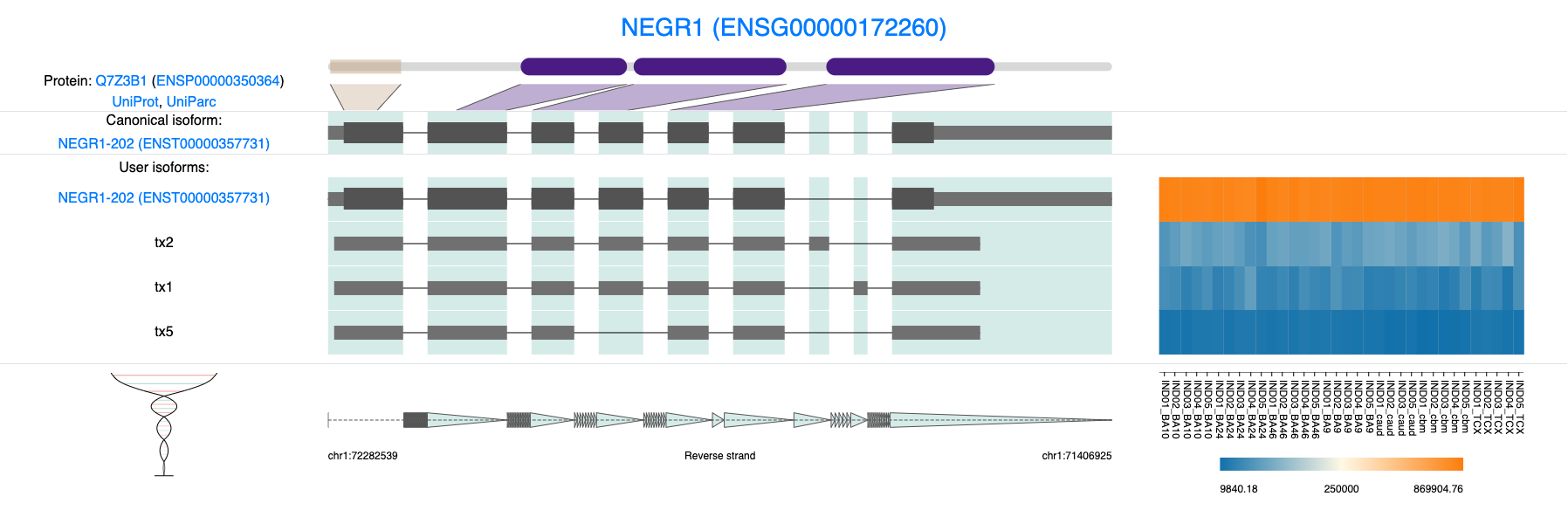


**W.** *PLCG1*. Protein track shows consensus disorder predicted regions in green.

**
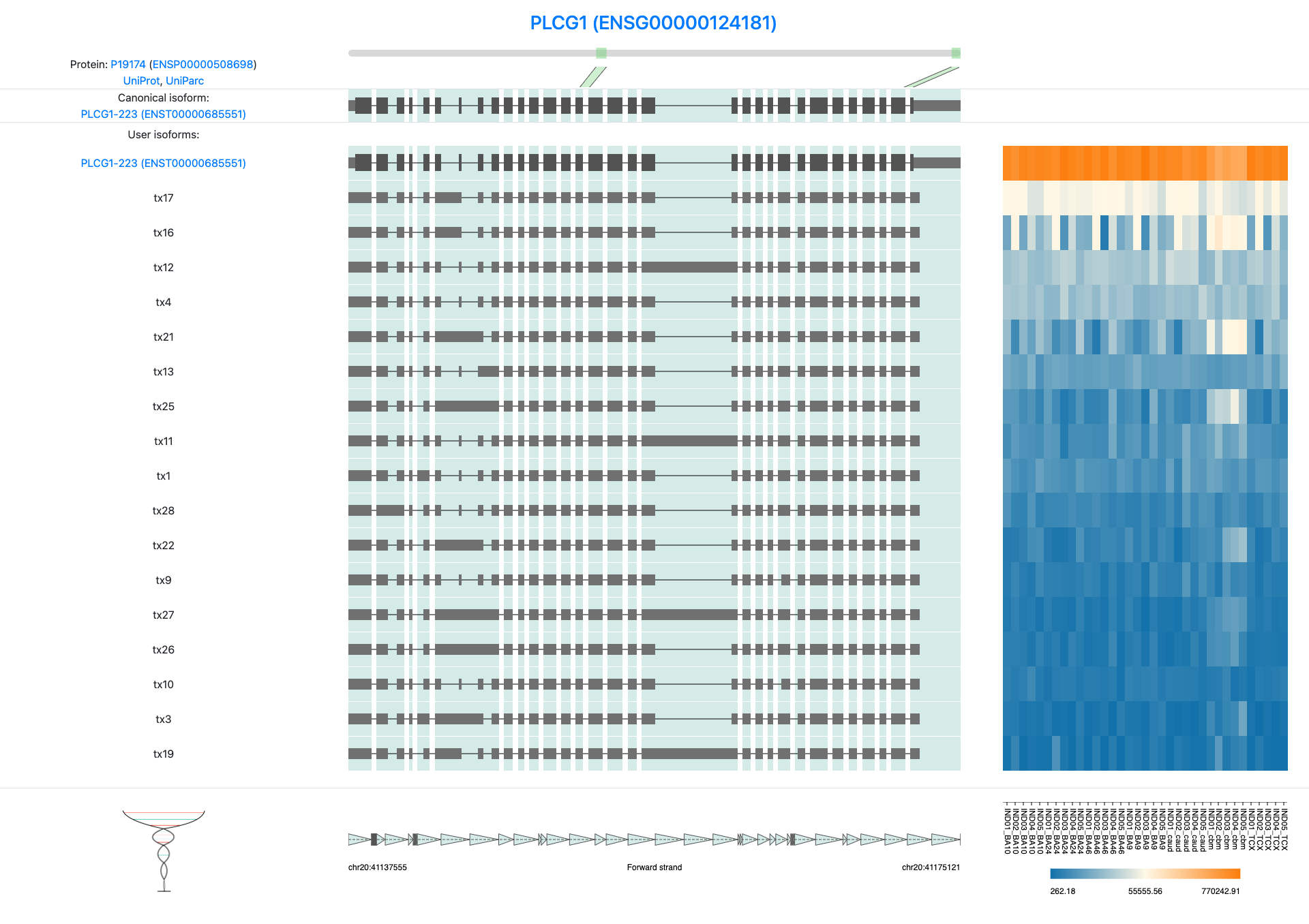
**

**X.** *PRMT7*. Protein track indicates a single ribosomal protein L11 methyltransferase (purple).


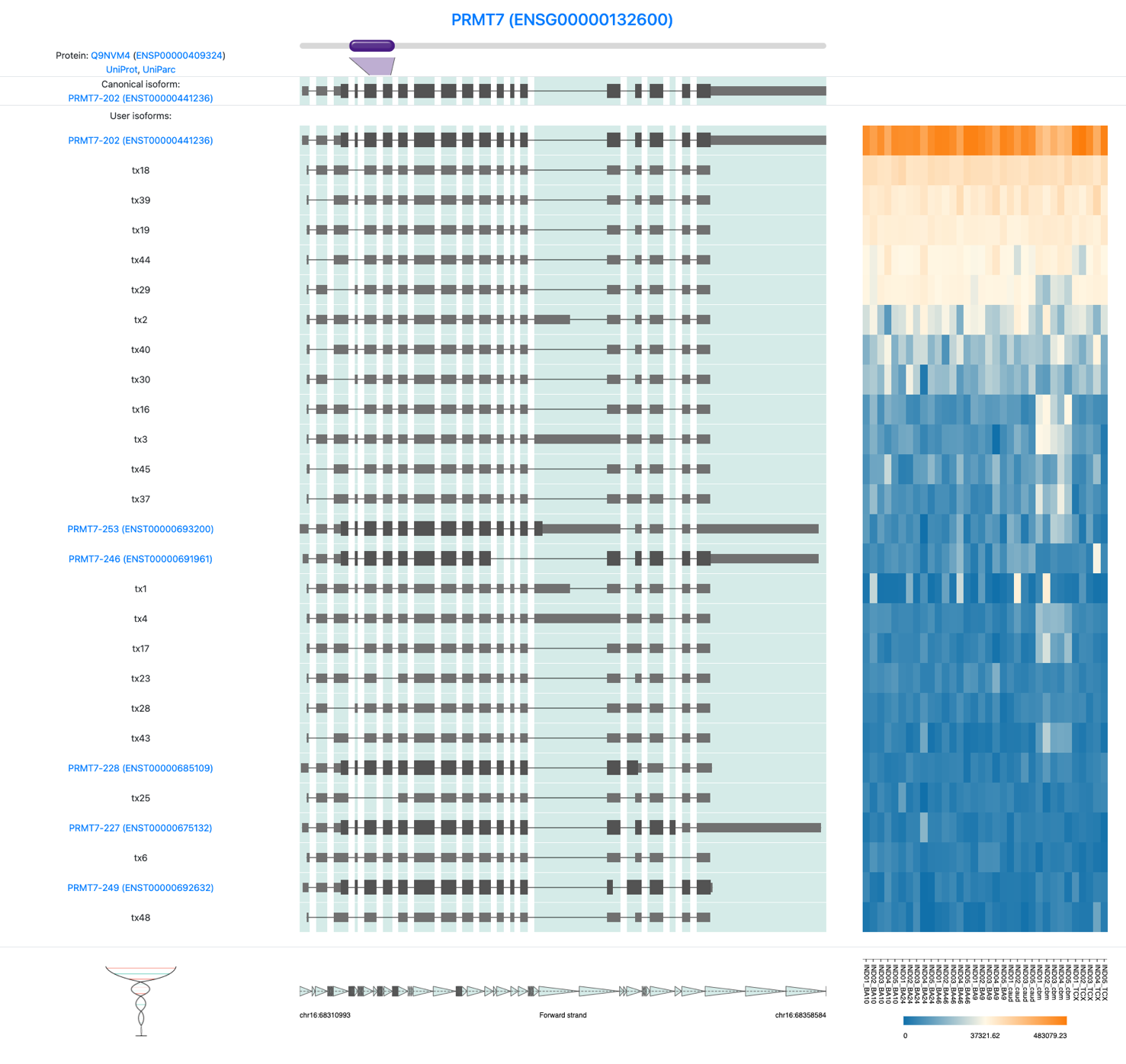


**Y.** *PTK2B*. Protein track shows consensus disorder predicted region (green) and coil (blue).

**
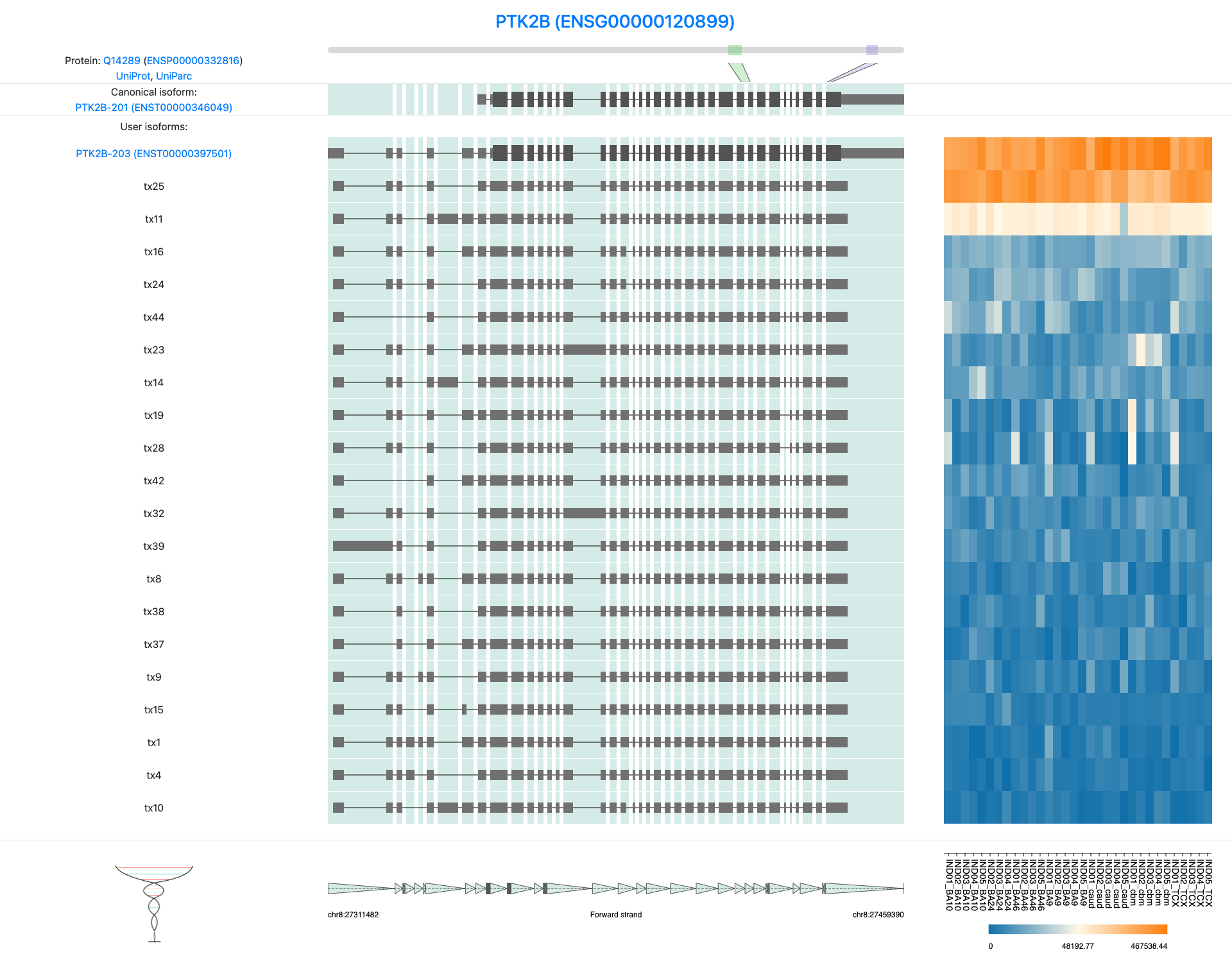
**

**Z.** *RFTN2*. Protein track indicates consensus disorder predicted regions (green) and raftlin (purple).


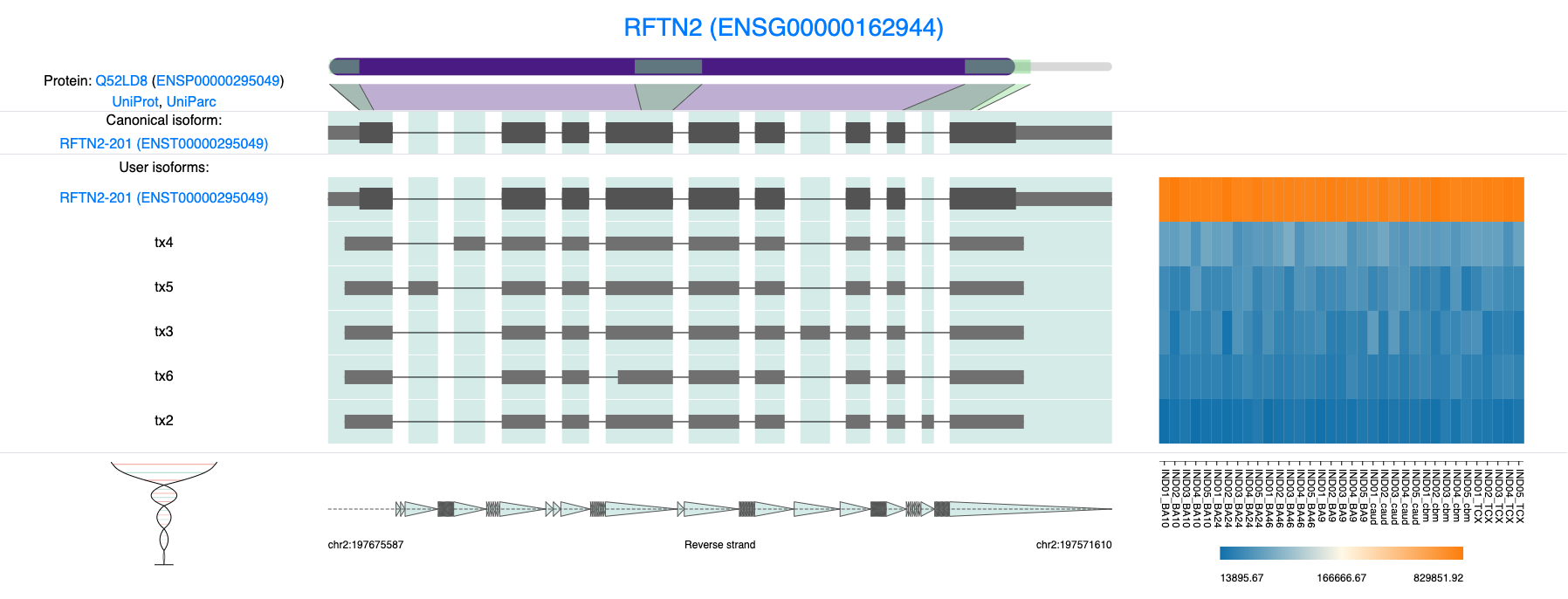


**AA.** *SLC30A9*. Protein track indicates cation efflux family domain (purple) and coil (blue).


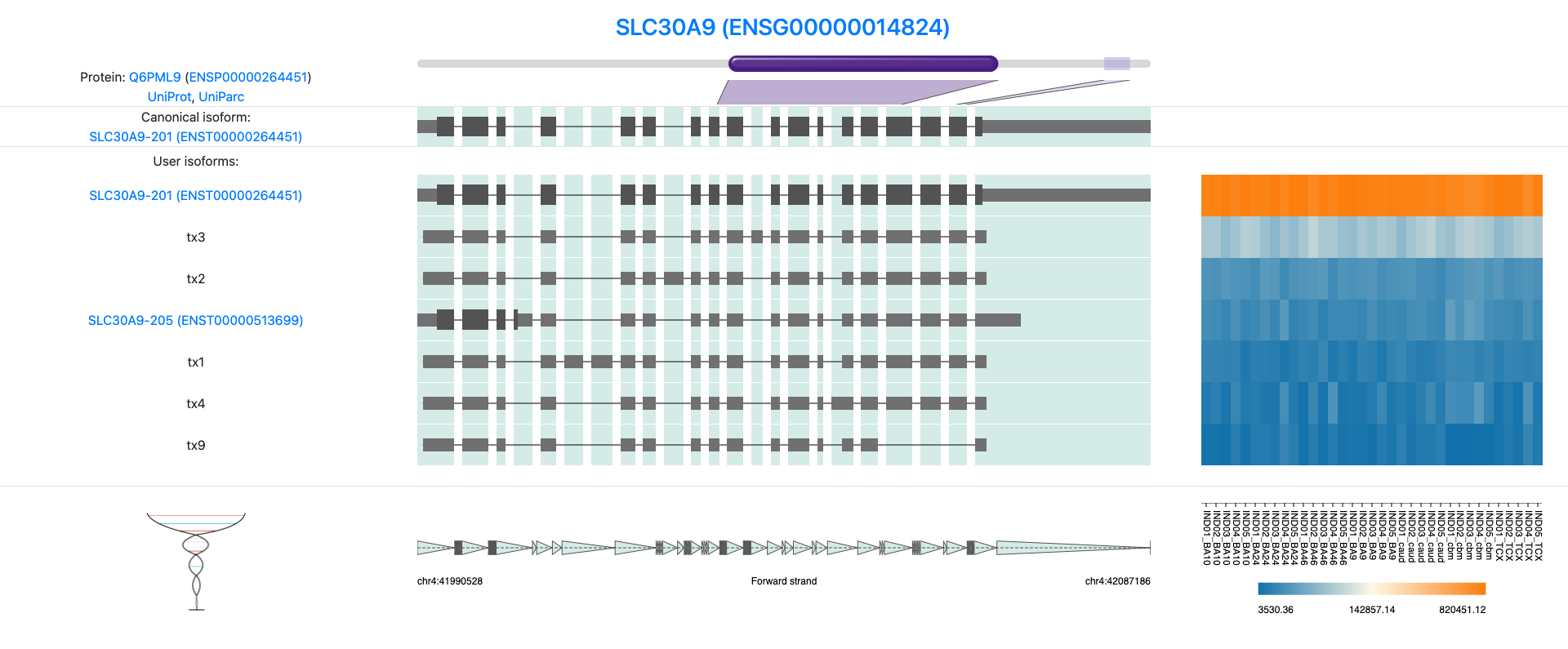


**AB.** *SNAP91*. Protein track consensus disorder predicted regions in green.


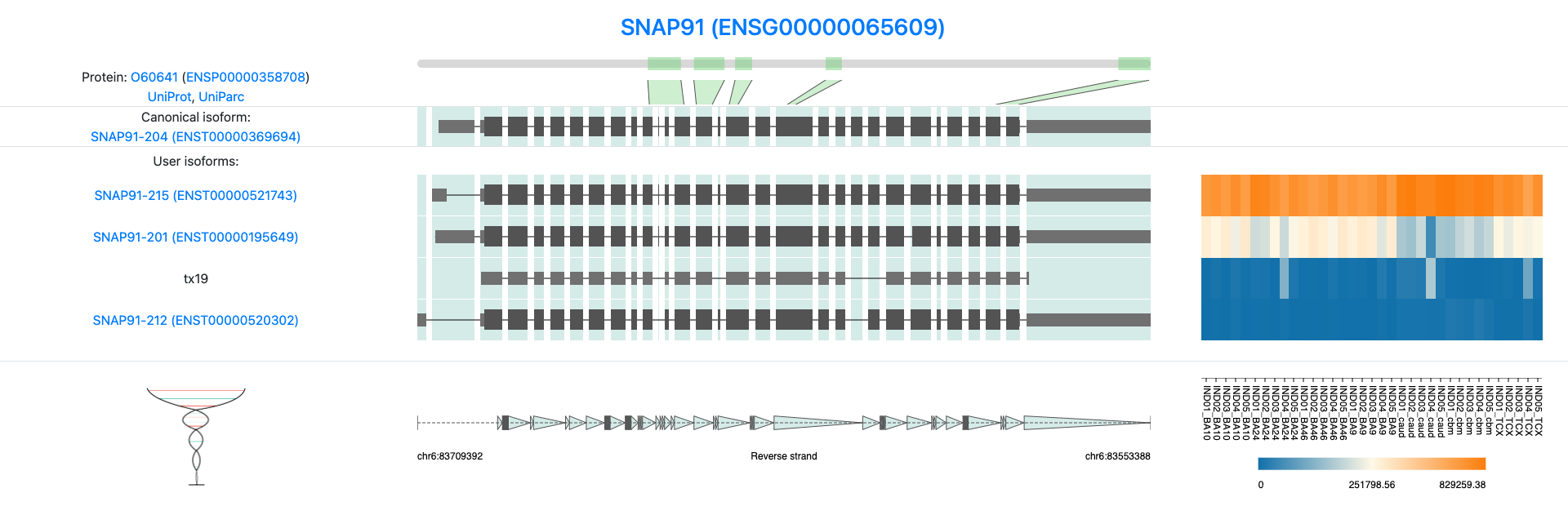


**AC.** *SORCS3*. Protein track consensus disorder predicted regions (green), sortilin, neurotensin 3 receptor 3, C-terminal and a PKD domain (purple).

**
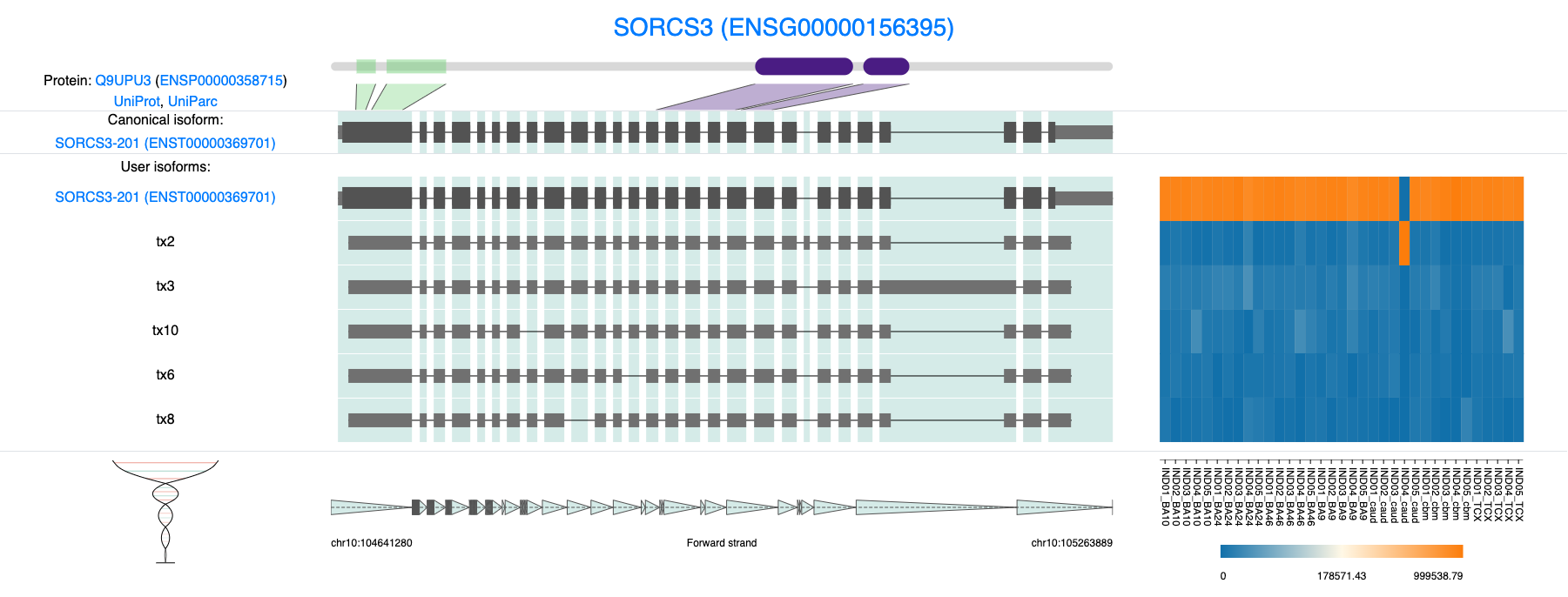
**

**AD.** *SPPL3*. Protein track indicates a signal peptide peptidase region (purple).

**
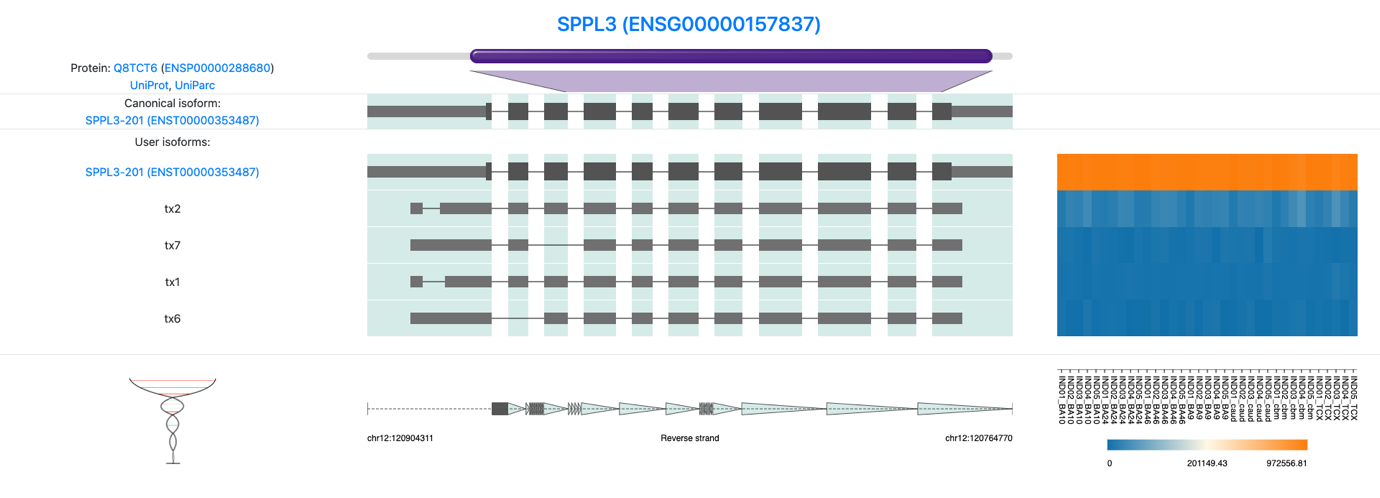
**

**AE.** *XRN2*. Protein track indicates XRN 5’-3’ exonuclease N-terminus and Xrn1 helical domain (purple), consensus disorder predicted regions are shown in green.


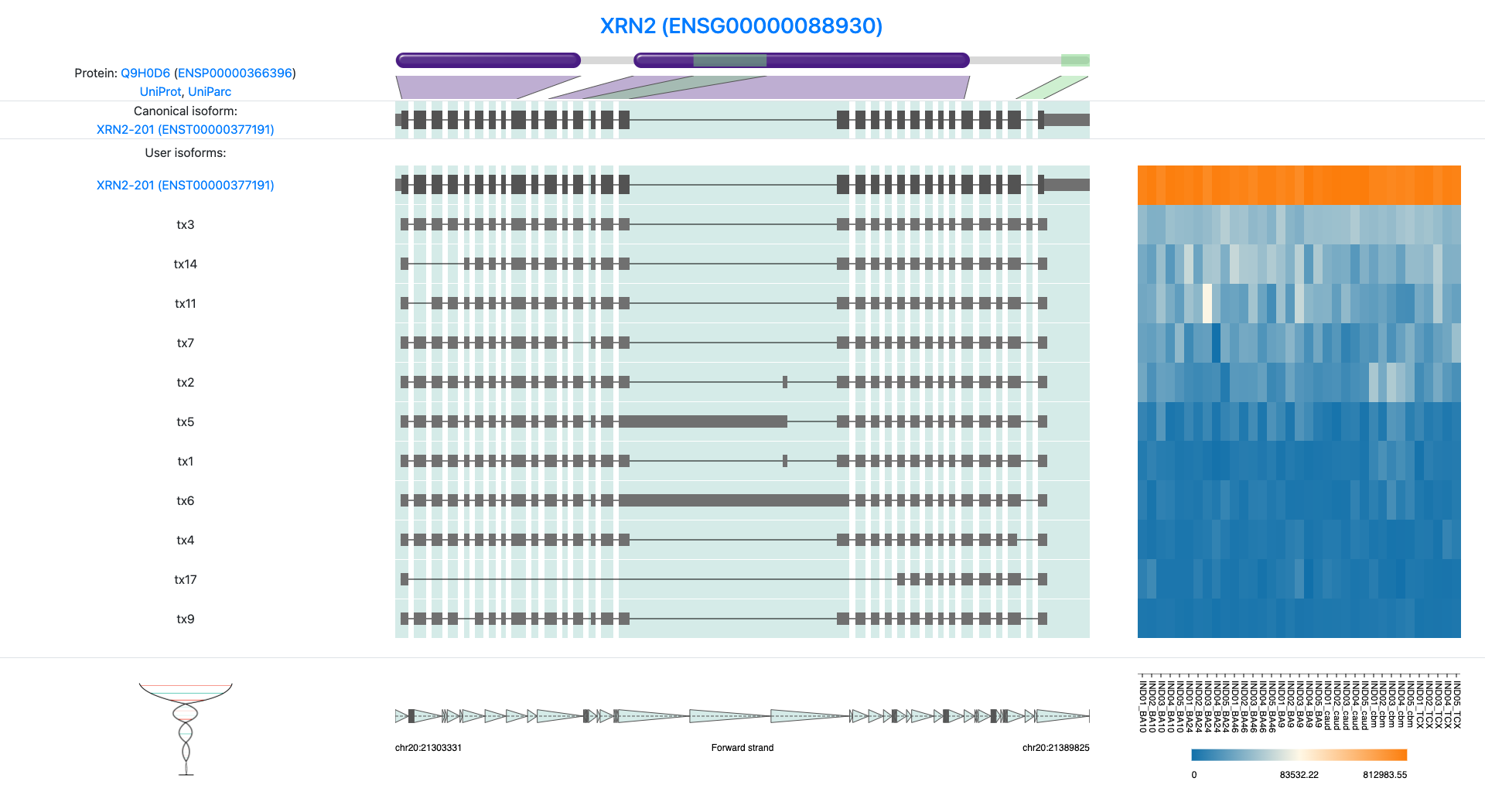


**AF.** *PACS2*. *PACS2* is involved in ion homeostasis in the endoplasmic reticulum and encodes 25 exons (amplicon: ~2.7 – 3.5 kb). We identified two novel exons (red boxes) in Txs 1 and 2. Novel Tx2 exon 20 was 135 nt in length with moderate CBM enrichment and predicted to be frame retaining, encoding 945 aa if translated. The protein track below indicates N-terminal C2 domain of phosphofurin acidic cluster sorting protein and PACS1 cytosolic sorting protein (purple), consensus disorder predicted regions are shown in green.

**
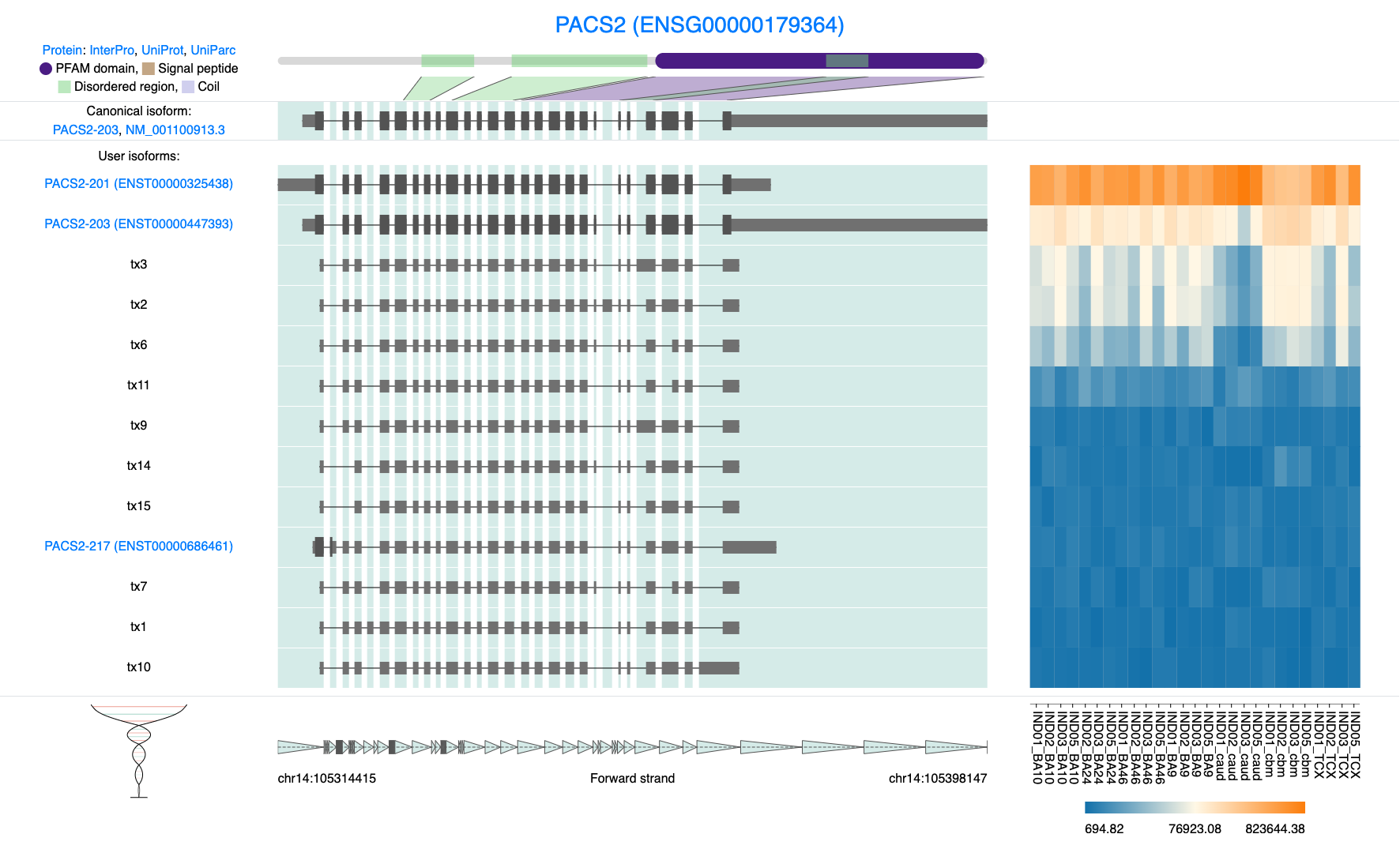
**

**AG.** *PREX1*. *PREX1* is guanine nucleotide exchange factor, involved in apoptosis and has two annotated isoforms in UCSC [1]. The canonical isoform for *PREX1* has 40 coding exons amounting to a ~5.1 kb amplicon. Despite the potential for abundant alternative splicing, we only identify two unannotated RNA isoforms (Tx14 and 18), both with novel canonical, but uncommon, splice donor/acceptor GC-AG pairing in the first exon. In Tx14, this novel splicing event led to a 61 nt deletion and PTC in 3’ canonical exon 5. Interestingly in Tx18, a similar splicing event and deletion (69 nt) is frame retaining (1636 aa) and appears to remove a phosphoserine site, which may impact its activity [2]. **A.** Protein track indicates consensus disorder predicted regions in green and coil (blue). **B.** A zoomed in view showing two unannotated splicing (GC/AG) events in exon 1, leading to a PTC (Tx14) and frame retention (Tx18).

**A**


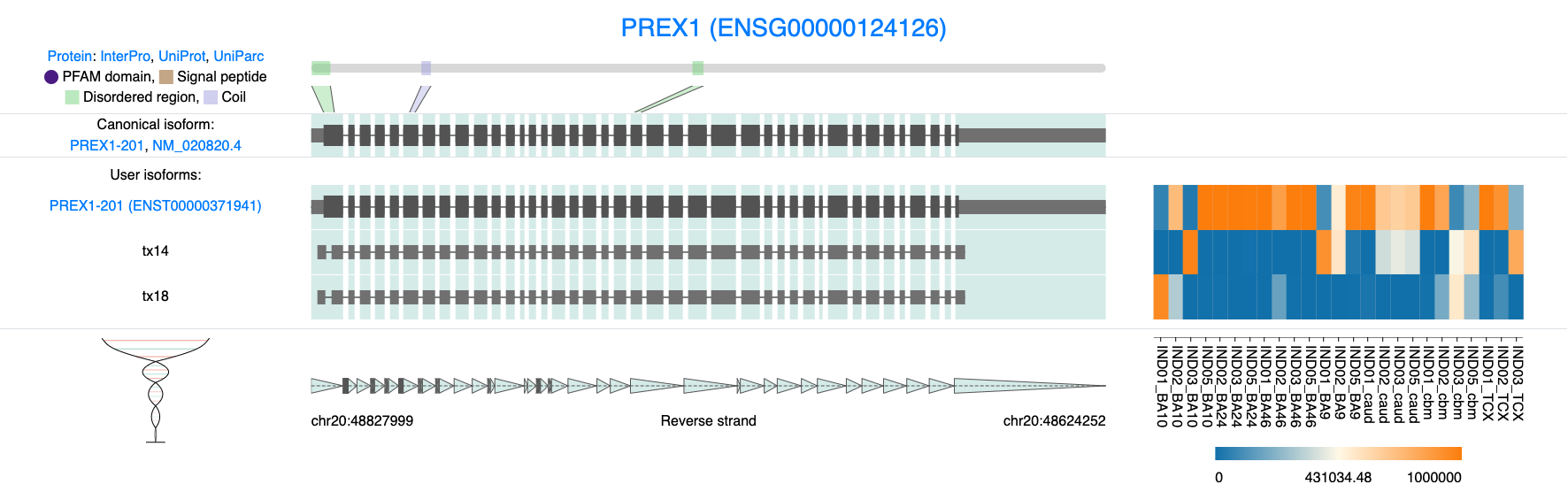


**B**


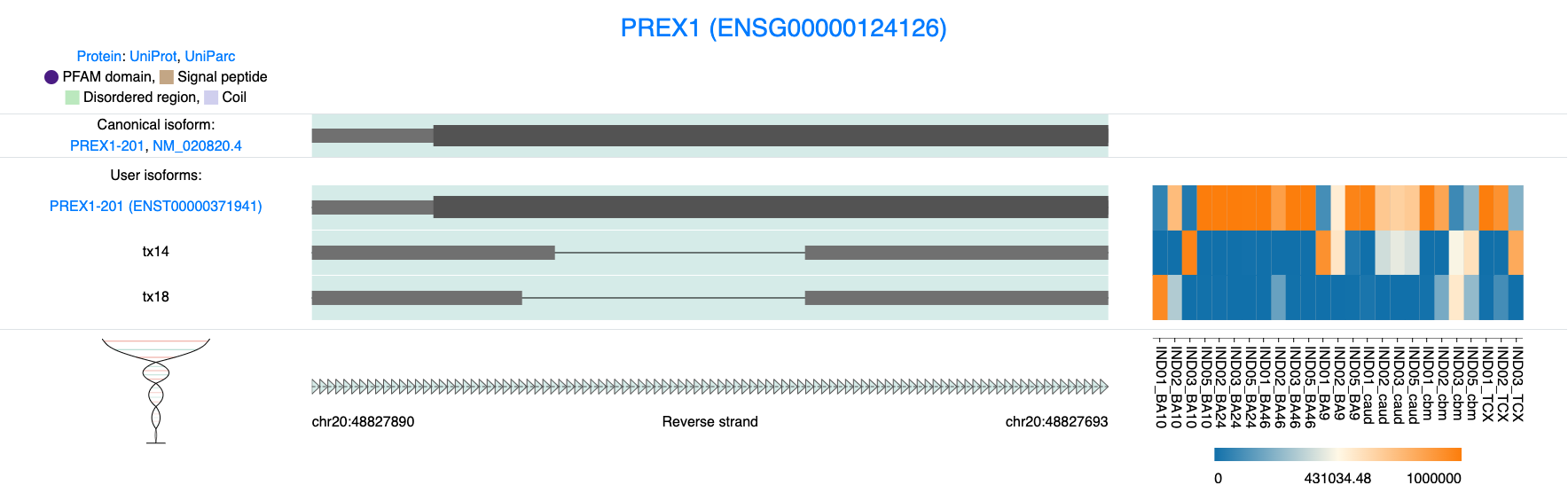


**References**

1. Negi SK, Guda C: **Global gene expression profiling of healthy human brain and its application in studying neurological disorders.** *Scientific Reports* 2017, **7:**897.

2. Barrows D, He JZ, Parsons R: **PREX1 Protein Function Is Negatively Regulated Downstream of Receptor Tyrosine Kinase Activation by p21-activated Kinases (PAKs).** *J Biol Chem* 2016, **291:**20042-20054.
