# Supplementary material for: Long-read sequencing reveals the RNA isoform repertoire of neuropsychiatric risk genes in human brain
Source: Genome Biol. 2025 Sep 23;26:298. doi: 10.1186/s13059-025-03724-1 (PMC12455821; doi:10.1186/s13059-025-03724-1)
Supplement: Supplementary file 7 — Additional file 7. Review history. [file 13059_2025_3724_MOESM7_ESM.docx]

Review History

**First round of review**

**Reviewer 1**

**Were you able to assess all statistics in the manuscript, including the appropriateness of statistical tests used?**

Yes, and I have assessed the statistics in my report.

**Comments to author:**

Genome Biology

Long-read sequencing reveals the RNA isoform repertoire of neuropsychiatric risk genes in human brain

Ricardo De Paoli-Iseppi1*, Shweta Joshi1, Josie Gleeson1, Yair David Joseph Prawer1, Yupei You2,3, Ria Agarwal1, Anran Li1, Anthea Hull1, Eloise Marie Whitehead1, Yoonji Seo1, Rhea Kujawa1, Raphael Chang1, Mriga Dutt1, Catriona McLean4,5, Benjamin Leo Parker1, Michael Ben Clark1*


Manuscript ID: GBIO-D-24-00527

This manuscript by De Paoli-Iseppi et al. builds a pipeline for gene-level targeted ONT long read sequencing to identify unannotated transcripts for disease relevant genes. The authors apply this pipeline to identify 363 novel isoforms and 28 novel exons of 31 genes, selected based on their association with various mental health disorders (MHDs). Thirty-one primers were designed (Supplementary Table 3) based on sequences in 5'- and 3'- UTRs of each canonical isoform. mRNA isolated from 7 regions (BA9, BA46, BA10, BA24, caudate, cerebellum, and temporal cortex) of 5 normal human brains (age range: 51-72 yrs.) used as template. Hundreds of isoforms were generated after RT-PCR with conditions optimized for individual gene. Resultant amplicons were processed by ONT barcoding kit, followed by long-read sequencing. A pipeline "IsoLamp" was created for isoform identification and analysis and found to outperform other isoform discovery tools (FLAIR, FLAMES, and Stringtie2, Figure 2) using a spike in dataset of SIRVs. The pipeline appears to be mostly just an extension of Bambu with slightly different filtering and parameters. Results are visualized by IsoVis, another published tool recently developed by the authors, which shows CDS of a canonical isoform, stacks of isoforms, and expression levels (heatmaps) (Figure 4-7, Additional_File_2). Because of the PCR based amplification, a limitation is that isoforms identified here represent variations in intronic splicing (e.g., exon skipping, novel exon inclusion, etc), but do not account for alternative transcript start sites / alternative promoter usage, alternative termination sites, or alternative polyadenylation which are thought to contribute more substantially transcript-isoform differences (PMID: 29202200). Altogether, this manuscript provides a blueprint for uncovering and beginning to characterize new isoforms for disease relevant genes. It applies this blueprint to 31 MHD relevant genes, finding many new isoforms. Future work will be necessary to establish the disease relevance, and function, of these newly identified isoforms.


Major comments:

Method Development - The beginning of the paper is focused on the "IsoLamp" method development and its comparison with other tools using spike in (SIRV) data. However, I found the description of the method itself to be lacking in detail in the main results. What exactly is the advance here over Bambu with "optimized parameters"? What are the optimized parameters, besides NDR which is discussed? What is the significance of these parameters and filtering?

IsoLamp employs an "optimized expression-based filter" (line 157). What does this mean? Why are these lowly expressed transcripts likely false positives?

Benchmarking - The SIRV benchmarking is useful, but important details are missing. The use of "Insufficient" and "Over" complete annotations needs to be better defined in the main text and figure 2. What proportion was missing, over how many random samplings, etc? Figure 2 should show actual data points in the correlations, not bar charts. There is no reason one should plot both pearson and spearman correlations on the same data - either the data are normally distributed or not…

TPM normalized expression data is almost always log-transformed when analyzed using linear models, which does not appear to be the case here (e.g. Fig 8). This should be justified

While there is evidence used to prioritize the MHD risk genes, these still should be called "candidate" risk genes. For example, although CLU is selected as the candidate risk gene for SCZ here, there is also compelling evidence that the "causal" gene at this locus is CHRNA2


Methods
- Why were there two different DNA polymerases used?
-


Minor comments:

p8-L230-231: (in Figure 4) "17.69% N = 80 FSM" … should be N = 78 (p6-L196)
p9-L239: (in Figure 4 legend) Expasy [55] was used … should be [44]
5. p20-L543: "in the human brain and demonstrates how … " should be "demonstrate … "
p21-L562-563: "mutations in which have previously been found in SZ patients and which resulted in a SZ-like phenotype in mice [65]. " English incorrect
p21-L579-581: "Twenty-two novel isoforms were identified for calcium channel gene CACNA1C and supporting previous findings [36] the top ten novel isoforms, by TPM, were classified as frame retaining, supporting their potential to generate functional proteins. " This is confusing
8. Supplementary Table 1: Individual A and B has never been mentioned in text, should be excluded.
9. Supplementary Table 6: Primers for validation of 10 genes. How these 10 genes are selected, randomly or purposely?

**Reviewer 2**

**Were you able to assess all statistics in the manuscript, including the appropriateness of statistical tests used?**

Yes, and I have assessed the statistics in my report.

**Comments to author:**

Paoli-Iseppi et al present a methodology IsoLamp, for analysis of amplicon lrRNA-seq data and apply this to the study isoform expression of neuropsychiatry risk genes in post-mortem brain samples.
Overall, I enjoyed Reading the manuscript and I believe a specific method methods for amplicon lrRNA-seq is a valuable contribution. The work is comprehensive and includes valuable validation approaches. However, I do have some serious concerns on the quality of the analysis strategy that need to be addressed, as well as the significance of the results in terms of the aspects listed below.

Overall approach
The study uses healthy donor post-mortem samples and analyzes specifically mental health disorders (MHD) risk genes, trying to make conclusions about the relationship of novel isoforms with MHD. However, it might be that the results reflect simply processes associated to death (e.g. lack of brain oxygen) and are not specific of MHD. As no other genes have been evaluated, there is no way to make the association of the observed isoform variation to this type of diseases.
Isoform calling
I worry about the fact that all isoform detection analyses have been done on the pooled reads. This impairs the identification of consistently expressed isoforms across samples and basically eliminates the possibility to extrapolate their conclusions to the Alzheimer disease. The LRGASP benchmarking effort demonstrated the existence of a large number of "novel" transcript variants that are sample-specific (they do not replicate) and which biological significance is unclear. This check is particularly important in this amplicon-based analyses as it is hard to evaluate otherwise if any of the novel isoforms is just a spurious isoform found in one sample, which has been preferentially amplified, to novel isoforms that are consistently present across samples. Therefore, the analyses should be repeated by calling isoforms in individual samples and then retaining only isoforms consistently expressed in multiple samples.
This type of separated analysis should also be done with the SIRV data (great validation strategy, by the way) and inspect the sources of false positives: are these a PCR amplification problem, is this a sequencing problem or is this a pipeline reconstruction algorithm problem? While this may not affect conclusions on performance comparison among tools, it can highlight which are the aspects of the technology for further improvement. Additionally, I found figure 2A hard to read. Maybe splicing this in 2 panels (one for precision, one for recall) an making it a barplot, as Figure 2B, would help. Moreover, since conclusions for Pearson and Spearman correlations are similar, one of the two could be sent to supplementary.
Novel exon validation
In the same line, it is unclear if the novel exons were validated in how many samples. Was the RT-PCR performed on a pooled RNA sample or on each RNA sample independently. Again, this is important to understand the significance and overall applicability of the results
Proteomics validation
The manuscript indicates validation of one novel exon. However, it would be good to have a broader overview of the Proteomics results. How many of the novel proteins could be validated?
Minor
Please update the SQANTI v4.2 does not exist. This should be SQANTI3, which is also the version that included the subcategories used in this manuscript. Please include the correct reference to SQANTI3: PMID: 38509328.

**Authors Response**

**Point-by-point responses to the reviewers’ comments:**

We thank the reviewers for their thoughtful comments on our manuscript. We have made a number of revisions to the manuscript and performed additional analyses in response. We believe we have successfully addressed all reviewer concerns and hope our manuscript will now be suitable for publication in Genome Biology.

Changes to our manuscript are shown in track changes

In addition, we have also made the following minor changes:

- In-text citations and reference to our IsoVis tool has been updated to the published reference. See reference #44. All reference numbers have been updated.
- Sentences have been rewritten for clarity.
- A reference our long-read amplicon user guide on protocols.io has been added to Methods section: Primer design, cDNA synthesis and long-range PCR
- Minor grammar and spelling corrections.
- Table 1 has been included in the Word document as a table object.
- Figure placement and figure legend placement now accords with standard journal style.

In our response, editor and reviewer comments are in black, our responses are in dark blue.

Reviewer reports:

**Reviewer #1**: Genome Biology

This manuscript by De Paoli-Iseppi et al. builds a pipeline for gene-level targeted ONT long read sequencing to identify unannotated transcripts for disease relevant genes. The authors apply this pipeline to identify 363 novel isoforms and 28 novel exons of 31 genes, selected based on their association with various mental health disorders (MHDs). Thirty-one primers were designed (Supplementary Table 3) based on sequences in 5'- and 3'- UTRs of each canonical isoform. mRNA isolated from 7 regions (BA9, BA46, BA10, BA24, caudate, cerebellum, and temporal cortex) of 5 normal human brains (age range: 51-72 yrs.) used as template. Hundreds of isoforms were generated after RT-PCR with conditions optimized for individual gene. Resultant amplicons were processed by ONT barcoding kit, followed by long-read sequencing.

A pipeline "IsoLamp" was created for isoform identification and analysis and found to outperform other isoform discovery tools (FLAIR, FLAMES, and Stringtie2, Figure 2)
using a spike in dataset of SIRVs. The pipeline appears to be mostly just an extension of Bambu with slightly different filtering and parameters.

We have addressed this in the ‘major comments’ section below.

Results are visualized by IsoVis, another published tool recently developed by the authors, which shows CDS of a canonical isoform, stacks of isoforms, and expression levels (heatmaps) (Figure 4-7, Additional_File_2). Because of the PCR based amplification, a limitation is that isoforms identified here represent variations in intronic splicing (e.g., exon skipping, novel exon inclusion, etc), but do not account for alternative transcript start sites / alternative promoter usage, alternative termination sites, or alternative polyadenylation which are thought to contribute more substantially transcript-isoform differences (PMID: 29202200).

We acknowledge the importance of alternative transcript start and termination sites in shaping transcript isoform diversity. We specifically address this limitation of amplicon sequencing in the **Discussion**:

*“Long-read amplicon sequencing, while providing an extremely sensitive isoform quantification method, is limited by the set(s) of primers used to amplify each risk gene. Our method aimed to locate primers within the 5’ and 3’ UTRs in proximity to the canonical translational start and stop codons. This approach generally amplified the entire coding sequence but does not capture full-length isoforms or variation in the UTRs and additional primers must be made to capture alternative unique start and termination sites.”*

However, to capture as much transcript variation as reasonably possible (including alternative first and last exons) we specifically assessed each candidate gene for well-supported alternative start and end sites and designed primer pairs accordingly. Therefore, 13/31 of our putative target risk genes had multiple start and/or termination site primers. For example, in *CLCN3*, additional forward primers enabled the identification of a short isoform (Tx9) with moderate expression levels, as shown in Additional File 4: Fig. E. We have revised the **Experimental overview** section and noted the additional primers designed in the **Primer design, cDNA synthesis and long-range PCR** sections to make our overall design clearer:

*“Amplicons were designed cover the full coding region of target genes and, where possible, run from the first to the last exon. Multiple set of primers were used for genes with alternative transcriptional initiation and termination exons and/or alternative coding sequence initiation and termination sites to profile as many potential alternative isoforms as possible.”*

We are familiar with studies such as Reyes et al (2018) which was nominated by the reviewer. However, we believe one key outcome of our work is that previous studies underestimated internal isoform variability. The additional sensitivity of gene-targeted sequencing, plus the greater ability of long-read approaches to characterise internal splicing changes, allow us to overcome this technical limitation. This is supported by the fact that 82% of the risk gene isoforms we discovered were novel despite quite conservative filters for transcript isoform discovery.

Altogether, this manuscript provides a blueprint for uncovering and beginning to characterize new isoforms for disease relevant genes. It applies this blueprint to 31 MHD relevant genes, finding many new isoforms. Future work will be necessary to establish the disease relevance, and function, of these newly identified isoforms.  

**Major comments:**

Method Development - The beginning of the paper is focused on the "IsoLamp" method development and its comparison with other tools using spike in (SIRV) data. However, I found the description of the method itself to be lacking in detail in the main results. What exactly is the advance here over Bambu with "optimized parameters"? What are the optimized parameters, besides NDR which is discussed? What is the significance of these parameters and filtering?

IsoLamp employs an "optimized expression-based filter" (line 157). What does this mean? Why are these lowly expressed transcripts likely false positives?

We thank the reviewer for their feedback. We have revise and expanded on the description of IsoLamp in the Results section (**IsoLamp: a tool for RNA isoform discovery from long-read amplicon sequencing**), Methods section: **Isoform discovery from long-read amplicon sequencing with IsoLamp**) and Figure 1 & 2 legends.

While our IsoLamp pipeline builds upon Bambu as the foundation for isoform identification, it introduces several key enhancements that improve functionality and relevance of isoform output for use with Nanopore amplicon sequencing.

1. Optimised (and tuneable) isoform discovery parameters including the *novel discovery rate (NDR)*; *samples_minimum filter (proportion of samples an isoform needs to be detected in); and TPM_minimum filter (minimum expression level for an isoform).* These parameters were optimised by extensive testing and benchmarking on the SIRV controls. Together these parameters maximise isoform precision and recall.
2. Easy to use for non-bioinformaticians, requiring only FASTQ files as data input and a parameters file which can be easily edited from the provided template on GitHub.
3. Incorporates tuneable down-sampling of reads to normalise read depth between samples.
4. Primer position filtering to remove any off-target or incomplete isoforms.
5. Automated generation of initial plots and reports, allowing for immediate analysis and visualisation – including with our free webtool IsoVis (<https://isomix.org/isovis/>).

Together these make our pipeline easy for researchers to analyse Nanopore amplicon sequencing of candidate gene(s).

Lastly, our SIRV benchmarking, as well as investigating different expression cutoffs in real data, show many isoforms with very low reported levels of expression are likely false positives. While an isoform that is under the expression cutoff is not necessarily a false positive, it is more likely to be so. We also emphasise that the expression filters are all tuneable parameters and can be changed or switched off by a user if they so choose. We have the **IsoLamp** results section to clarify. Ie:

*“IsoLamp employs an expression-based filter to remove isoforms that do not meet a user-defined expression threshold in a specified proportion of samples (Fig. 1B, see Methods). The default values of this filter were optimised based on the removal of known false positives from the SIRV benchmarking data (SIRV_O) described above and enable IsoLamp to remove possible false positive isoforms in amplicon data”.*

Benchmarking - The SIRV benchmarking is useful, but important details are missing. The use of "Insufficient" and "Over" complete annotations needs to be better defined in the main text and figure 2. What proportion was missing, over how many random samplings, etc? Figure 2 should show actual data points in the correlations, not bar charts. There is no reason one should plot both pearson and spearman correlations on the same data - either the data are normally distributed or not…

We apologise for any confusion regarding our use of the SIRVs. We have updated both the Methods and Results to better explain the different SIRV annotations. These are annotation sets provided by the supplier (Lexogen) that allow researchers to test the sensitivity and specificity of their isoform discovery.

To address this comment, we have edited Figure 2. As requested, Figure 2B now shows the Spearman correlation data as individual points. Figure 2A has been remade to match the style of the new Figure 2B and points made larger. The figure legend has also been edited for clarity and to add further SIRV reference annotation details.

Updated text includes:

Results **IsoLamp** section:

*“We assessed the precision, recall and quantitative accuracy of the five tools using three different SIRV reference annotations provided by Lexogen (Fig. 2, Additional file 1: Table S2, Methods):* ***1.*** *Complete - contains all SIRV isoforms (SIRV_C, N=69),* ***2.*** *Insufficient – missing 26 SIRV isoforms that are present in the mixes (SIRV_I, N=43), and* ***3.*** *Over – contains an additional 31 isoforms that are not present in the SIRV mixes (SIRV_O, N=100).*”

Methods **Isoform discovery from long-read amplicon sequencing with IsoLamp** section:

*“...the programs were compared using three SIRV reference annotations: Complete (C), Incomplete (I) (missing isoforms, to test ability to recover unannotated true positive isoforms) and Over (O) (annotation contains extra isoforms not present in mixes, to test ability to minimise false positive annotated isoforms). These references were downloaded from Lexogen under ‘Additional annotations’ (https://www.lexogen.com/sirvs/download/). Specific information on SIRV isoform annotation for each gene can be found in the SIRV Set 1 user guide (pp. 25-28). Briefly there are 69 SIRV isoforms in the Complete reference (SIRV_C), 25 (36.2%) of these are removed from the Insufficient reference (N=44, SIRV_I) and 31 (44.9%) are added to the Over-annotated reference (N=100, SIRV_O). Additionally, we removed one extra SIRV (502) from the insufficient annotation to ensure multiple amplified SIRV5 isoforms were missing from the annotation and increase the stringency of our benchmarking.”*

TPM normalized expression data is almost always log-transformed when analyzed using linear models, which does not appear to be the case here (e.g. Fig 8). This should be justified

We have repeated our brain region enriched isoform analysis as suggested by the reviewer using log-transformed TPM data. A one-way ANOVA was run on the log-transformed data to determine if the tissue enrichment of the target isoform was statistically significant (corrected for multiple comparisons with Tukey). Figures 8A, B and C have been updated to represent this updated analysis. Our interpretation of the results with regards to the brain region enrichment of these specific isoforms remains unchanged.

The proportion of overall expression for each isoform has now been moved to Additional file 2: Fig S15AC. Subsequent references to supplementary figures have been altered.

While there is evidence used to prioritize the MHD risk genes, these still should be called "candidate" risk genes. For example, although CLU is selected as the candidate risk gene for SCZ here, there is also compelling evidence that the "causal" gene at this locus is CHRNA2

We selected our candidate risk genes based on the strength of evidence they cause risk for a mental health disorders. However, we acknowledge that identifying causal genes is not straightforward. We have made changes throughout all sections of the manuscript to make clear that the genes selected for RNA isoform study are candidate risk genes. We have also avoided making an assertion that these genes/isoforms *cause* disease.

**Method**
-       Why were there two different DNA polymerases used?

During PCR amplicon optimisation and testing we found that some taq polymerases produced better results (more efficient amplification in few PCR cycles, no/fewer off-target amplicons) for different genes. NEBLongAmp Taq was used as the default, however for some longer amplicons it performed poorly and so we moved to SuperFi Taq and/or PrimeSTAR GXL to generate the PCR products.

To make this clearer the following has been added to the Methods section **Primer design, cDNA synthesis and long-range PCR:**

“*LongAmp® Taq was tested first by default, however if it performed poorly an alternative polymerase was tested and optimised to keep the number of PCR cycles to a minimum*.”

**Minor comments**
1. p8-L230-231: (in Figure 4) "17.69% N = 80 FSM" …      should be N = 78 (p6-L196)

This has been corrected and a new Figure 4A uploaded with resubmission.

2. p9-L239: (in Figure 4 legend) Expasy [55] was used … should be [44]

This citation has been corrected in the text

3. p20-L543: "in the human brain and demonstrates how  … "      should be "demonstrate … "

This has been corrected in text.

4. p21-L562-563: "mutations in which have previously been found in SZ patients and which resulted in a SZ-like phenotype in mice [65]. "      English incorrect

We have revised this sentence: “Mutations in *KLC1* have previously been found in SZ patients, while a mouse heterozygous mutant resulted in a SZ-like phenotype”

5. p21-L579-581: "Twenty-two novel isoforms were identified for calcium channel gene CACNA1C and supporting previous findings [36] the top ten novel isoforms, by TPM, were classified as frame retaining, supporting their potential to generate functional proteins." This is confusing

This sentence has been revised: “Twenty-two novel isoforms were identified for calcium channel *CACNA1C.* Consistent with previous findings [36], the top ten novel isoforms, ranked by TPM, were classified as frame retaining, supporting their potential to generate functional proteins.”

6. Supplementary Table 1: Individual A and B has never been mentioned in text, should be excluded.

Supplementary Table 1 (now Additional File 1: Table S1) has been edited to remove Individuals A and B. Please see resubmitted table.

7. Supplementary Table 6: Primers for validation of 10 genes. How these 10 genes are selected, randomly or purposely?

Due to the time involved in lab validations we selected all novel exons identified as of March 2023 for validation. We validated 100% of these novel exons. By manuscript submission in April 2024 updates to the results and analysis had identified an extra 7 novel exons. Since there was no selection for exons to validate based on expression or any other parameter (and the validation rate was 100%), we conclude that all novel exons are likely to be real.

**Reviewer #2**: Paoli-Iseppi et al present a methodology IsoLamp, for analysis of amplicon lrRNA-seq data and apply this to the study isoform expression of neuropsychiatry risk genes in post-mortem brain samples.
Overall, I enjoyed Reading the manuscript and I believe a specific method methods for amplicon lrRNA-seq is a valuable contribution. The work is comprehensive and includes valuable validation approaches. However, I do have some serious concerns on the quality of the analysis strategy that need to be addressed, as well as the significance of the results in terms of the aspects listed below.

Overall approach
The study uses healthy donor post-mortem samples and analyzes specifically mental health disorders (MHD) risk genes, trying to make conclusions about the relationship of novel isoforms with MHD. However, it might be that the results reflect simply processes associated to death (e.g. lack of brain oxygen) and are not specific of MHD.  As no other genes have been evaluated, there is no way to make the association of the observed isoform variation to this type of diseases.

We would like to clarify that we are not trying to suggest that the novel isoforms we identify are causative in MHDs, as demonstrating this would require a population genetics study. Our aim was to define the isoform profiles of risk genes because we believe these are poorly annotated. By doing so we will enable research to associate genetic risk with the expression and/or presence of specific isoforms. We have edited the language in the Discussion to ensure that we are not suggesting the isoforms found in our study have a relationship with brain function / MHD onset or outcome except where cited with evidence from the literature, including previous risk or functional studies.

To address the reviewers’ second point, we agree that agonal state and/or hypoxia could affect isoform discovery and quantification and for this reason we carefully selected which samples to utilise in our study. Previous evidence has shown that brain pH is a key marker of hypoxia and other deleterious pre-death factors (Kingsbury et al. (1995), *Mol. Brain Res.*). Lower pH correlates with the severity of hypoxia and with decreasing RNA quality. We included both brain pH and RNA quality (measured by RIN) in selecting which samples to utilise. The post-mortem brain samples used for our study had RINs ranging from 6 - 8.1 (mean = 7.4), with a pH range indicative of little hypoxic insult (Fig. S4D). In contrast one individual we obtained post-mortem samples for had low RIN values and much lower pH, indicative of severe hypoxia and was therefore excluded from our study (Fig. S4D). Lastly, we did not observe instances of isoform expression (known or novel) unique to individuals, which further demonstrates our results are not simply reflecting processes associated with death.

To address the reviewer’s final point and demonstrate our expectation that novel isoforms are not unique to MHD risk genes, we sequenced two additional genes, *PACS2* and *PREX1*, that did not appear in our risk gene database (i.e. little to no evidence of risk for MHDs). Together these results demonstrate novel isoforms are not unique to risk genes and moderate to highly expressed novel isoforms are common in brain. IsoVis figures and a short explanation of some of these isoforms have been added to Additional File 4: Figs. AF-AG. The text now reads:

*“To confirm that novel isoforms were not specific to putative risk genes, we also sequenced two genes, PACS2 and PREX1, with little evidence for neuropsychiatric disorder risk (e.g. not found in our candidate risk gene list). In total, we identified 16 RNA isoforms, with 10 (~30% of overall TPM) and 2 (~44% overall TPM) novel isoforms for PACS2 and PREX1, respectively (Additional file 4,: Fig. 2AF – AG). These results demonstrate that identification of unannotated RNA isoforms is not limited to risk genes.”*

**Isoform calling**
I worry about the fact that all isoform detection analyses have been done on the pooled reads. This impairs the identification of consistently expressed isoforms across samples and basically eliminates the possibility to extrapolate their conclusions to the Alzheimer disease. The LRGASP benchmarking effort demonstrated the existence of a large number of "novel" transcript variants that are sample-specific (they do not replicate) and which biological significance is unclear. This check is particularly important in this amplicon-based analyses as it is hard to evaluate otherwise if any of the novel isoforms is just a spurious isoform found in one sample, which has been preferentially amplified, to novel isoforms that are consistently present across samples. Therefore, the analyses should be repeated by calling isoforms in individual samples and then retaining only isoforms consistently expressed in multiple samples.

The reviewer raises a very important point about the replicability of detected isoforms. Reporting only replicable detected isoforms was also a key concern for us. Therefore, the default behaviour of IsoLamp is to only report isoforms identified in ≥25% of samples. This is the data that we reported in the original version of our manuscript. We believe this is the optimal way to perform the analysis as it allows us to gain the computational efficiency and discovery potential of using pooled reads, while ensuring we only report replicable isoforms (and not have results driven by sample or individual donor specific expression).

IsoLamp does this using the ‘*samples_minimum*’ parameter. This can be modified by the user to increase discovery sensitivity or replicability as per their needs. We have improved our description of the default IsoLamp behaviour and provided more information about this parameter in the Methods section. The text now reads:

*“Reads from each barcode were then quantified with salmon in alignment-based mode (v0.14.1) [88]. An expression filter can be applied at this stage by setting two optional, user definable, parameters: TPM_minimum (minimum expression level for an isoform) and samples_minimum (proportion of samples an isoform must meet the TPM minimum threshold in). The default values based on SIRV optimisation are 5000 TPM and 25% of samples, respectively. The application of this filter to our brain samples required isoforms to meet a threshold of 5000 TPM in a minimum of 8 brain samples.”*

To directly address the reviewer’s concern about the identification of isoforms present in only 1 sample, and to demonstrate the ‘*samples_minimum*’ parameter works correctly, we investigated the isoforms identified for several putative risk genes when *samples_minimum* was set to 1, 2, 8 and 35 samples. Our results demonstrated this parameter works as expected filtering out isoforms found in less than the specified number of samples. We have added the following to the results section:

*“The IsoLamp expression filter required identified isoforms to be present in 25% of samples. We confirmed IsoLamp was only identifying replicable isoforms by running it with the samples_minimum parameter set to: 1 (3% samples), 2 (6%) and 35 (100%) for selected genes. For DCC, we identified 3 isoforms that were only expressed in 1 sample, all of which were removed by our default filtering (Additional file 2: Fig. S7A). Similar outcomes were shown for ATG13, GRIA1 and CLCN3 demonstrating that isoforms arising from a single sample, or with low sample support, were being effectively removed (Additional file 2: Fig. S7B-D).”*

This type of separated analysis should also be done with the SIRV data (great validation strategy, by the way) and inspect the sources of false positives: are these a PCR amplification problem, is this a sequencing problem or is this a pipeline reconstruction algorithm problem?  While this may not affect conclusions on performance comparison among tools, it can highlight which are the aspects of the technology for further improvement.

We thank the reviewer for this interesting suggestion. Our optimised SIRV pipeline used the same “*samples_minimum”* parameter and expression filter (TPM=5000) as our risk isoform work, therefore we can be confident we are not identifying true positives or false positives based on single sample detections.

Prior to initial submission we spent significant time looking at false positives and optimising IsoLamp to have excellent recall, while maintaining high precision. False positives largely had very low expression, which is partially why IsoLamp has multiple filters, including an expression filter. Our efforts showed determining the cause of false positives can be very difficult. For example, IsoLamp identities 1 false positive SIRV (sirv 517 in the Over-annotated set). Our investigation of this showed raw reads matching SIRV517 were present in many samples. Thus, it didn’t appear to be a pipeline/IsoLamp issue, while we wouldn’t expect rare PCR issues or sequencing errors to identify SIRV517 in many samples. Overall, we don’t know why this false positive is detected and cannot rule out the possibility it is present in the sample (even though it shouldn’t be). We can say however, that IsoLamp is performing as expected and reporting a consistently detected isoform.

As for technology development. In general, we have recommended that users use as few cycles of PCR for a gene to reduce the chance of negative impacts from over-amplification. We also mention in the discussion that the implementation of unique molecular identifiers (UMI) would be a particularly beneficial upgrade to amplicon sequencing of this nature.

*“To help overcome such issues, future long-read amplicon sequencing could incorporate unique molecular identifiers to tag molecules prior to PCR to ensure an accurate representation of the original RNA isoforms [78].”*

Additionally, I found figure 2A hard to read. Maybe splicing this in 2 panels (one for precision, one for recall) an making it a barplot, as Figure 2B, would help. Moreover, since conclusions for Pearson and Spearman correlations are similar, one of the two could be sent to supplementary.

In response to feedback from both reviewers, Figure 2 has been remade. Figure 2A has been replicated with a cleaner style and larger data points. Figure 2B (Spearman correlation) has been retained and the Pearson correlation removed.

**Novel exon validation**
In the same line, it is unclear if the novel exons were validated in how many samples. Was the RT-PCR performed on a pooled RNA sample or on each RNA sample independently. Again, this is important to understand the significance and overall applicability of the results

As all novel isoforms and thus novel exons had to be present in at least 25% of samples in our sequencing results we considered validation in a single sample as sufficient.

Novel exon validation was a two-step process as confirming many novel exons required substantial PCR optimisation. Initially we tested the PCR on a single sample that the novel exon containing transcript showed robust expression in. Across all 21 exons we utilised 13 samples across 6 brain regions. If this PCR provided a single on-target band, we proceeded straight to confirmatory sequencing. If an exon required PCR optimisation we performed these on a pool of 4 tissues (to help preserve individual tissues samples and also improve detection probability). Once detected we proceed to confirmatory sequencing from the sample pool.

Overall, we validated three genes using cDNA from a single sample and seven genes using pooled cDNA.

We have included these details in the revised manuscript to ensure greater clarity.

We have added the following to the Methods:

“*Nanopore long-read supported novel exons were validated by RT-PCR. Amplification was initially tested in a single sample shown by nanopore sequencing to express the novel exon (13 samples across 6 brain regions were used). Successful amplification was confirmed by Sanger sequencing. For exons which required PCR optimisation, a pool of 4 post-mortem brain samples were utilised, followed by Sanger sequencing.*"

**Proteomics validation**
The manuscript indicates validation of one novel exon. However, it would be good to have a broader overview of the Proteomics results. How many of the novel proteins could be validated?

Using the mass spectrophotometry methods originally described in the manuscript we were able to validate the RNA isoform change to the ITIH4 protein. As part of our revisions, we have examined a separate human PM brain mass spectrometry dataset using untargeted MS with fractionation for deeper sampling. This confirmed translation of a novel isoform with an exon skipping event in *GABBR2*. Therefore, we have now confirmed translation of two novel proteoforms. The additional text reads:

*“Using untargeted MS, we also confirmed a novel skipping event of cassette exon 5 (244 – 266) in gamma-aminobutyric acid type B receptor subunit 2 (GABBR2), which was identified in novel Tx29 (Additional file 4: Fig. M). The Tx29 protein is missing multiple features including an alpha helix, beta strand and disulphide bond forming cysteine (position 266) (Additional file 2: Fig. S13A – C). The correct formation of GABBR2 subunits is critical for assembly of the human gamma-aminobutyric acid type B receptor (GABABR), which plays an important role in inhibitory neurotransmission in the brain [49]. Disruption of the GABBR2 structure in Tx29 may negatively impact this receptor, however further functional testing is required.”*

We have also revised the method section: **Protein isolation and novel sequence detection using mass spectrophotometry (MS)** to describe the samples used for this MS and untargeted analysis with fractionation.

To date we have been able to validate these two novel proteoforms. This degree of proteomic validation is not unexpected given: **1.** Amplicon RNA-seq is much more sensitive than mass-spectrometry for isoform identification due to its targeted approach; **2.** Brain has an incredibly complex proteome; **3.** Many of the novel isoforms only have small segments of novel amino acid sequence to identify them by, making identification at the proteoform-level challenging; 4. A much more exhaustive search for novel isoform translation would be required to comprehensively determine which novel proteoforms are present, but this was not the primary aim of this project.

**Minor**
Please update the SQANTI v4.2 does not exist. This should be SQANTI3, which is also the version that included the subcategories used in this manuscript. Please include the correct reference to SQANTI3: PMID: 38509328.

The original citation has been edited to the PMID provided by the reviewer: “Pardo-Palacios FJ, Arzalluz-Luque A, Kondratova L, P. Salguero, J. Mestre-Tomás, R. Amorín, et al. SQANTI3: curation of long-read transcriptomes for accurate identification of known and novel isoforms. *Nature Methods* 2024, **21**:793-797.”

**Second round of review**

**Reviewer 1**

The authors have addressed all my comments
